# Comparative proximity biotinylation implicates the small GTPase RAB18 in sterol mobilization and biosynthesis

**DOI:** 10.1016/j.jbc.2023.105295

**Published:** 2023-09-28

**Authors:** Robert S. Kiss, Jarred Chicoine, Youssef Khalil, Robert Sladek, He Chen, Alessandro Pisaturo, Cyril Martin, Jessica D. Dale, Tegan A. Brudenell, Archith Kamath, Jeffrey Kyei-Boahen, Anouar Hafiane, Girija Daliah, Célia Alecki, Tayah S. Hopes, Martin Heier, Irene A. Aligianis, Jean-Jacques Lebrun, Julie Aspden, Emanuele Paci, Anja Kerksiek, Dieter Lütjohann, Peter Clayton, Jimi C. Wills, Alex von Kriegsheim, Tommy Nilsson, Eamonn Sheridan, Mark T. Handley

**Affiliations:** 1 Cardiovascular Health Across the Lifespan (CHAL) Program, Research Institute of the McGill University Health Centre, Montreal, Quebec, Canada; 2 Metabolic Disorders and Complications (MEDIC) Program, Research Institute of the McGill University Health Centre, Montreal, Quebec, Canada; 3 Genetics and Genomic Medicine, Great Ormond Street Institute of Child Health, University College London, London, United Kingdom; 4 Leeds Institute of Medical Research, St James's University Hospital, Leeds, United Kingdom; 5 MRC Human Genetics Unit, Institute of Genetics and Cancer, University of Edinburgh, Edinburgh, United Kingdom; 6 Division of Medical Sciences, University of Oxford, Oxford, United Kingdom; 7 Department of Medicine, McGill University Health Centre, CHAL Research Program, Montreal, Canada; 8 Department of Medicine, McGill University Health Centre, Cancer Research Program, Montreal, Canada; 9 Department of Biochemistry, McGill University, Montreal, Quebec, Canada; 10 Faculty of Biological Sciences, University of Leeds, Leeds, United Kingdom; 11 Department of Clinical Neuroscience for Children, Oslo University Hospital, Oslo, Norway; 12 Medical and Developmental Genetics, Medical Research Council Human Genetics Unit, Edinburgh, United Kingdom; 13 Astbury Centre for Structural Molecular Biology, University of Leeds, Leeds, United Kingdom; 14 Institute of Clinical Chemistry and Clinical Pharmacology, University Hospital Bonn, Bonn, Germany; 15 Cancer Research United Kingdom Edinburgh Centre, Institute of Genetics and Cancer, University of Edinburgh, Edinburgh, United Kingdom; 16 Firefinch Software Ltd, Edinburgh, United Kingdom; 17 Cancer Research Program (CRP), Research Institute of the McGill University Health Centre, Montreal, Quebec, Canada

**Keywords:** Rab, protein–protein interaction, lipid transport, cholesterol metabolism, SNARE proteins, BioID, RAB18, EBP, ORP2, lathosterol

## Abstract

Loss of functional RAB18 causes the autosomal recessive condition Warburg Micro syndrome. To better understand this disease, we used proximity biotinylation to generate an inventory of potential RAB18 effectors. A restricted set of 28 RAB18 interactions were dependent on the binary RAB3GAP1–RAB3GAP2 RAB18–guanine nucleotide exchange factor complex. Twelve of these 28 interactions are supported by prior reports, and we have directly validated novel interactions with SEC22A, TMCO4, and INPP5B. Consistent with a role for RAB18 in regulating membrane contact sites, interactors included groups of microtubule/membrane-remodeling proteins, membrane-tethering and docking proteins, and lipid-modifying/transporting proteins. Two of the putative interactors, EBP and OSBPL2/ORP2, have sterol substrates. EBP is a Δ8-Δ7 sterol isomerase, and ORP2 is a lipid transport protein. This prompted us to investigate a role for RAB18 in cholesterol biosynthesis. We found that the cholesterol precursor and EBP-product lathosterol accumulates in both RAB18-null HeLa cells and RAB3GAP1-null fibroblasts derived from an affected individual. Furthermore, *de novo* cholesterol biosynthesis is impaired in cells in which RAB18 is absent or dysregulated or in which ORP2 expression is disrupted. Our data demonstrate that guanine nucleotide exchange factor–dependent Rab interactions are highly amenable to interrogation by proximity biotinylation and may suggest that Micro syndrome is a cholesterol biosynthesis disorder.

Rab proteins are a large subfamily of small GTPases with discrete roles in coordinating membrane trafficking (1). Like other small GTPases, they adopt different conformations and enter into different protein–protein interactions according to whether they are GDP bound or GTP bound. Although they possess some intrinsic GTP-hydrolysis activity, their nucleotide-bound state in cells is tightly governed by two classes of regulatory proteins. Guanine-nucleotide exchange factors (GEFs) catalyze the exchange of bound GDP for GTP, whereas GTPase-activating proteins (GAPs) promote the hydrolysis of bound GTP to GDP (2, 3).

Biallelic loss-of-function variants in *RAB18*, *RAB3GAP1*, *RAB3GAP2*, or *TBC1D20* cause the autosomal recessive condition Warburg Micro syndrome (4, 5, 6, 7, 8) (Mendelian Inheritance in Man IDs: 600118, 614222, 614225, 615663, and 212720). *RAB3GAP1* and *RAB3GAP2* encode subunits of the binary RAB18–GEF complex “RAB3GAP,” whereas *TBC1D20* encodes an RAB18–GAP (9, 10). Thus, the same pathology is produced when functional RAB18 is absent or when its normal regulation is disrupted. However, it is unclear how RAB18 dysfunction contributes to disease pathology at a molecular level.

Rab proteins fulfil their roles by way of protein–protein interactions with interacting partners termed “effectors.” The identification of these proteins can therefore provide insight into these roles. However, biochemical identification of Rab effectors is challenging; Rab-effector interactions are usually GTP dependent and are often highly transient. Immunoprecipitation, affinity purification (AP), and yeast-2-hybrid approaches have each been used but may be more or less effective depending on the Rab isoform studied (11, 12).

One newer approach is “BioID” proximity biotinylation utilizing Rab proteins fused to mutant forms of the biotin ligase BirA. The Rab fusion protein biotinylates proximal proteins that are then purified on streptavidin and identified through mass spectrometry (MS) (13, 14, 15, 16). Biotin labeling occurs in a relatively physiological context, and prospective effectors can be purified under high stringency conditions. However, a drawback of the technique is that it does not distinguish between close associations resulting from functional protein–protein interactions and those resulting from overlapping localizations.

To discriminate functional RAB18 interactions, we compared BirA∗-RAB18 labeling of protein in WT HeLa cells with that in cells in which RAB18–GEF activity was disrupted with CRISPR. Known and novel effectors were more strongly labeled in the WT cells. Twenty eight RAB18 interactions were categorized as RAB3GAP dependent. These proteins comprised several groups. Proteins within each group were clearly interrelated through involvement in connected biological processes. Moreover, gene-disease associations within the set included multiple overlapping phenotypes.

The most studied groups of RAB18 effector proteins to date are the tethering factors that comprise the NRZ–Dsl complex (ZW10, NBAS, and RINT1) and the endoplasmic reticulum (ER) SNARE proteins that comprise the Syntaxin18 complex (STX18, BNIP1, USE1, and SEC22B) (17, 18, 19, 20). Although SNARE complexes typically mediate membrane fusion, it has been proposed that RAB18 interacts with these proteins, *via* ZW10, in an RAB3GAP-dependent manner, to mediate the close apposition of membranes to facilitate lipid transfer (17). It has also been suggested that SEC22B is dispensable for this function (17). In lipid-loaded cells, active RAB18 becomes enriched on lipid droplets (LDs) and recruits the NRZ and SNARE proteins. This is thought to regulate membrane contacts between LDs and the ER and to mediate LD maturation and possibly biogenesis (17, 19, 21). RAB18-null or depleted cells exhibit normal fatty acid uptake but reduced triacylglycerol synthesis as well as reduced basal and stimulated lipolysis (17). This is in agreement with prior studies suggesting it functions in both lipogenesis and lipolysis (22, 23, 24).

Our data elaborate the existing model suggesting that RAB18 effectors act collectively in lipid transfer at membrane contact sites (MCSs) (17). We identify multiple proteins already implicated in the establishment and maintenance of membrane contacts including NRZ and SNARE components. We verify novel interactions with SEC22A, TMCO4, and INPP5B using immunoprecipitation of exogenously expressed fusion proteins. We also identify putative RAB18 interactors involved in sterol biosynthesis and mobilization, the Δ8-Δ7 sterol isomerase EBP, and the lipid transport protein OSBPL2/ORP2. The putative interaction with EBP led us to examine sterol profiles and cholesterol biosynthesis in several cell lines. We find that a sterol product of EBP catalysis—lathosterol—accumulates in RAB18-null HeLa cells and RAB3GAP1-null human primary fibroblasts. Furthermore, that cholesterol biosynthesis is reduced in cells in which RAB18 is absent or dysregulated and altered in cells with altered constitutive RAB18 activity. Interestingly, disruption of ORP2 expression in cells stably expressing WT or constitutively active RAB18 reduces cholesterol biosynthesis to a similar baseline level, suggesting that it is required for this aspect of RAB18 function. Because Micro syndrome shares a number of features with known cholesterol biosynthesis disorders, these data provide a tentative indication that this deficit might partly underlie disease pathology.

## Results

### An inventory of RAB18–GEF-dependent RAB18-associated proteins in HeLa cells

**Figure 1:**
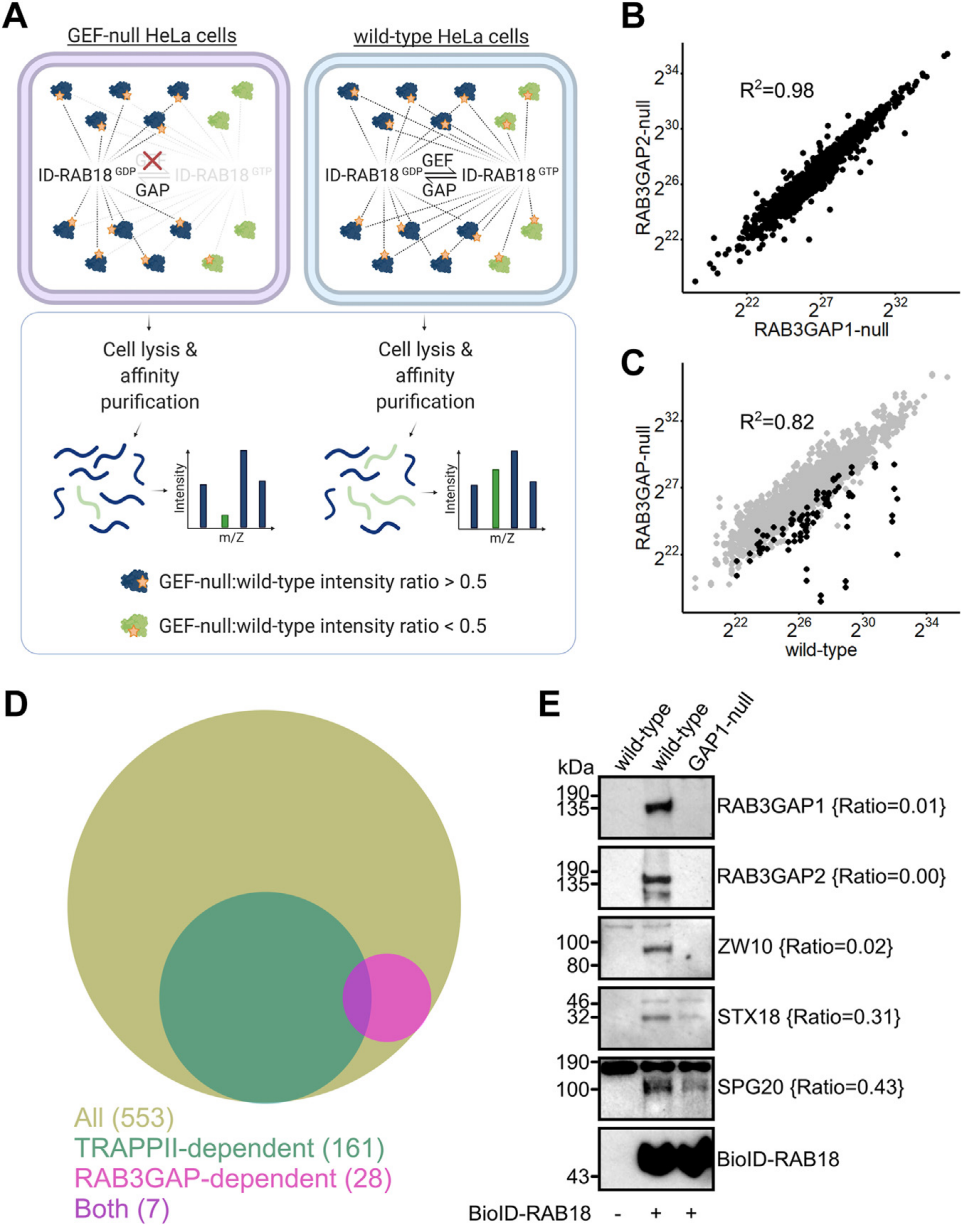
**RAB3GAP-dependent RAB18 interactions in HeLa cells.***A*, schematic to show experimental approach. Proximity biotinylation of guanine nucleotide exchange factor (GEF)-dependent interactors by BirA∗-RAB18 (ID-RAB18) is disrupted in GEF-null cells. GEF-independent interactors are biotinylated in both GEF-null and WT cells. Following affinity purification, GEF-dependent interactions are determined by label-free quantitative (LFQ) intensity ratios. *B*, plot to show correlation between Log_2_ LFQ intensities of individual proteins identified in samples purified from RAB3GAP1- and RAB3GAP2-null cells. *C*, plot to show correlation between Log_2_ LFQ intensities of individual proteins identified in samples purified from WT and RAB3GAP-null cells. Highlighted datapoints correspond to proteins later found to have RAB3GAP-null:WT intensity ratios ≤0.5. *D,* Venn diagram to show overlap between all RAB18 associations, TRAPPII-dependent interactions (TRAPPC9-null:WT intensity ratios <0.5), and RAB3GAP-dependent associations (RAB3GAP-null:WT intensity ratios <0.5). *E,* Western blotting of samples purified from WT and RAB3GAP1-null cells in an independent BioID experiment. Levels of selected proteins are consistent with RAB3GAP-null:WT intensity ratios {*braces*}.

We first used CRISPR to generate a panel of clonal, otherwise isogenic, HeLa cell lines null for RAB18 and a number of its regulators (Fig. S1). We then carried out proximity labeling using transient expression of the same exogenous BirA∗-RAB18 construct in RAB3GAP1-, RAB3GAP2-, and TRAPPC9-null cell lines and in WT cells (Fig. 1*A*). RAB3GAP1 and RAB3GAP2 are each essential subunits of a binary RAB18–GEF complex (9). TRAPPC9 is reported to be essential for the RAB18–GEF activity of a different GEF, the multisubunit TRAPPII complex (25).

Proximity labeling, AP, and MS of biotinylated proteins were carried out essentially as previously described (15, 26). Prior to MS analysis, samples from each of the streptavidin pull-downs were subjected to Western blotting to ensure comparable BirA∗-RAB18 expression (Fig. S2*A*). Label-free quantitative proteomics (LFQP) analyses were used to calculate “LFQ intensities” for each RAB18-associated protein (27). These were then normalized in each experiment according to the quantity of RAB18 found in each sample. Samples from three independent experiments were analyzed. Pull-downs from untransfected biotin-treated cells were used as controls. After filtering the data to remove known MS contaminants and any protein identified at a high level in control samples, a total of 902, 635, and 661 RAB18-associated proteins were identified in each experiment. A total of 553 proteins were present in two or more of the replicate experiments (Table S1).

Different Rab–GEF complexes may operate in distinct subcellular localizations and coordinate associations with different effectors (28). Therefore, we assessed whether nonzero intensities for each RAB18-associated protein correlated between samples (Figs. 1*B* and S2*B*). Very strong correlations between protein intensities from RAB3GAP1- and RAB3GAP2-null cells indicated that loss of either protein had a functionally equivalent effect (*R*^2^ = 0.98, Fig. 1*B*). In contrast, intensities from RAB3GAP1/2- and TRAPPC9-null cells were much more poorly correlated (*R*^2^ = 0.74, Fig. S2*B*). We therefore considered RAB3GAP- and TRAPPC9-dependent RAB18 interactions separately. Intensities from WT and RAB3GAP-null samples correlated with an *R*^2^ = 0.82, but a number of proteins showed reduced intensities in the RAB3GAP-null samples (Fig. 1*C*).

GEF activity promotes Rab GTP binding, and this is usually necessary for effector interactions. We therefore reasoned that levels of true effector proteins would be reduced in samples from GEF-null cells as compared with those from WT cells (Fig. 1*A*). We calculated GEF-null:WT intensity ratios for each RAB18-associated protein (Table S1). Only 28 proteins showed a RAB3GAP-null:WT ratio ≤0.5 (Figs. 1*D*, Tables 1 and Table S1). One hundred sixty-one proteins showed a TRAPPII-null:WT intensity ratio ≤0.5 (Fig. 1*D* and Table S1). There was only limited overlap between RAB3GAP- and TRAPPC9-dependent associations (Fig. 1*D*).

**Table 1:** RAB3GAP-dependent RAB18 interactions in HeLa cells

Protein	n	Ratio (see Fig. 1*A*)	Ortholog protein–protein interaction (18)	Additional evidence	Functional group
CAMSAP1	3	0.26			Microtubule/membrane remodeling
REEP4	3	0.38		Tinti *et al.*, 2012 (29)	
BICD2	2	0.28	BicD	Gillingham *et al.*, 2019 (13)	
SPG20	2	0.43	CG12001	This study (Fig. 2)^a^	
ZW10	3	0.02	mit(1)15	Xu *et al.*, 2018 (17); Gillingham *et al.*, 2019 (13)	Membrane tethering/docking
RINT1	3	0.16	CG8605	Xu *et al.*, 2018 (17)	
NBAS	3	0.16	rod	Xu *et al.*, 2018 (17); Gillingham *et al.*, 2019 (13)	
SCFD2	3	0.34	Slh	Gillingham *et al.*, 2019 (13)	
SEC22A	3	0.45		This study (Fig. 3)	
JPH1	3	0.50			
STX18	2	0.31	Syx18	Xu *et al.*, 2018 (17)	
BNIP1	2	0.37		Xu *et al.*, 2018 (17)	
TMCO4	3	0.06		This study (Fig. 4)	Lipid modifying/mobilizing
OSBPL2	3	0.49		This study (Fig. 5)^a^	
EBP	3	0.50		This study (Fig. 5)^a^	
INPP5B	2	0.00		This study (Fig. 5)	
C2CD2L	2	0.18			
C2CD2	2	0.34			
RAB3GAP2	3	0.00	CG7061/Rab3-GAP	Gerondopoulos *et al.*, 2014 (9)	RAB18–GEF complex
RAB3GAP1	3	0.01	CG31935	Gerondopoulos *et al.*, 2014 (9)	
MFHAS1	3	0.00	Lrrk		Other
TRIM13	3	0.35			
TMEM109	3	0.36			
PBXIP1	3	0.44			
SSR3	3	0.46			
ERGIC3	2	0.29			
SCARA3	2	0.42			
TMEM245	2	0.43			

Twenty eight proteins with mean RAB3GAP-null:WT intensity ratios ≤0.5, identified in two or more independent proximity biotinylation experiments. Ratios for each protein are calculated from normalized LFQ intensities in samples purified from RAB3GAP-null cells divided by those purified from WT cells. The mean of ratios from each experiment in which each protein was identified is shown. Orthologous proteins identified by Gillingham *et al.*, 2014, and other studies providing supporting evidence for interactions, are shown. Proteins are grouped according to their reported functions. The full dataset is provided in Table S1.

a Indirect additional evidence for this interaction is presented in this study.

The most comprehensive annotation of candidate RAB18 effectors thus far was made in the 2014 article by Gillingham *et al*. (18), which utilized an AP–MS approach and the *Drosophila* RAB18 ortholog. In that study, a total of 456 proteins were identified as interacting with RAB18. However, only 14 of these were well represented in terms of spectral counts, exhibited low nonspecific binding to GST/Sepharose and showed low binding to other Rab protein isoforms. We took these 14 proteins as the most plausible physiological RAB18 interactors and searched for these in our datasets.

Orthologs or paralogs of 11 of the 14 putative RAB18-interacting proteins identified by Gillingham *et al*. were identified as GEF dependent in our combined dataset. Ten of 14 proteins were among the 28 RAB3GAP-dependent associations (listed in Table 1). Four of 14 proteins were among the TRAPPII-dependent associations (Table S1). Nine of 14 RAB18 interactors from the study by Gillingham *et al*. and 12 of 28 of the RAB3GAP-dependent associations from our study have been described in other previously published works (9, 13, 17, 29).

For initial validation of our dataset and the reproducibility of our results, we carried out an additional independent BioID experiment with WT and RAB3GAP1-null cells and subjected the resulting samples to Western blotting for selected RAB18-associated proteins (Fig. 1*E*). As with the MS, these proteins showed either complete (RAB3GAP2, ZW10) or partial (SPG20, STX18) dependence on RAB3GAP for their RAB18 association.

We further validated our approach with additional proximity biotinylation experiments in human embryonic kidney 293 (HEK293) cells. We used cells stably expressing BirA∗-tagged RAB18 fusions incorporating WT RAB18, GTP hydrolysis–deficient RAB18(Gln67Leu) or nucleotide-binding deficient RAB18(Ser22Asn) mutants (Fig. S3, *A* and *B*). A total of 96 proteins were identified as associating with RAB18 across all samples (Table S2). Gln67Leu:WT intensity ratios for known RAB18 interactors ranged from 0.1 to 1.49 indicating that RAB18 associations were altered by the Gln67Leu variant but not predictably so. In contrast, Ser22Asn:WT intensity ratios were <0.5 for the majority of these proteins. About 28 nucleotide binding–dependent RAB18 associations included five of the RAB3GAP-dependent associations and seven of the TRAPPII-dependent associations seen in the HeLa cells (Fig. S3*C*). These data confirm that the loss of GEFs has similar effects on RAB18 interactions to direct loss of nucleotide binding. In addition, they support the differing regulation of specific RAB18 interactions by different GEFs.

### Validation screening of RAB3GAP-dependent RAB18 associations reveals reduced levels of SPG20 in RAB18-null and TBC1D20-null cells

Our continued study focused on the 28 RAB3GAP-dependent RAB18 associations identified in HeLa cells. Encouragingly, these appeared to share interconnected functions and fell into discrete groups (Table 1). Furthermore, genes encoding seven of the 28 proteins or their homologs are associated with inherited diseases that share features with Micro syndrome (Table 2).

**Table 2:** Genes encoding putative RAB18 effectors or their homologs are associated with diseases that share overlapping features with Warburg Micro syndrome

Gene(s)	Homolog(s)	Syndrome(s)	Inheritance	OMIM	Overlapping features
*RAB3GAP1*, *RAB3GAP2*, *RAB18*, and *TBC1D20*	-	Warburg Micro syndrome; Martsolf syndrome	AR	600118; 614222; 614225; 615663; 212720	Intellectual disability (ID), microcephaly (M), ascending spastic paraplegia (ASP), cataract (C), microphthalmia (Mo), microcornea (Mc), optic atrophy (OA), seizures (S), corpus callosum hypogenesis (CCH), cerebellar vermis hypoplasia (CVH), genital abnormalities (GA)
*EBP*	—	CDPX2; MEND syndrome	XLD; XLR	302960, 300960	ID, M, C, Mo, Mc, S, CCH, CVH, GA
*INPP5B*	*OCRL*; *INPP5K*	Lowe syndrome; MDCCAID	XLR; AR	309000; 607875	ID, M, C, S
*SSR3*	—	Congenital disorder of glycosylation	AR	Ng *et al.*, 2019 (97)	ID, M, CCH, GA
*SPG20*	—	Troyer syndrome (SPG20)	AR	275900	ID, M, ASP
*BICD2*	—	Spinal muscular atrophy	AD	615290; 615291	ASP
*REEP4*	*REEP1*; *REEP2*	SPG31; SPG72	AD	610250; 615625	ASP
*NBAS*	—	SOPH syndrome	AR	614800	OA

AD, autosomal dominant; AR, autosomal recessive; XLD, X-linked dominant; XLR, X-linked recessive.

**Figure 2:**
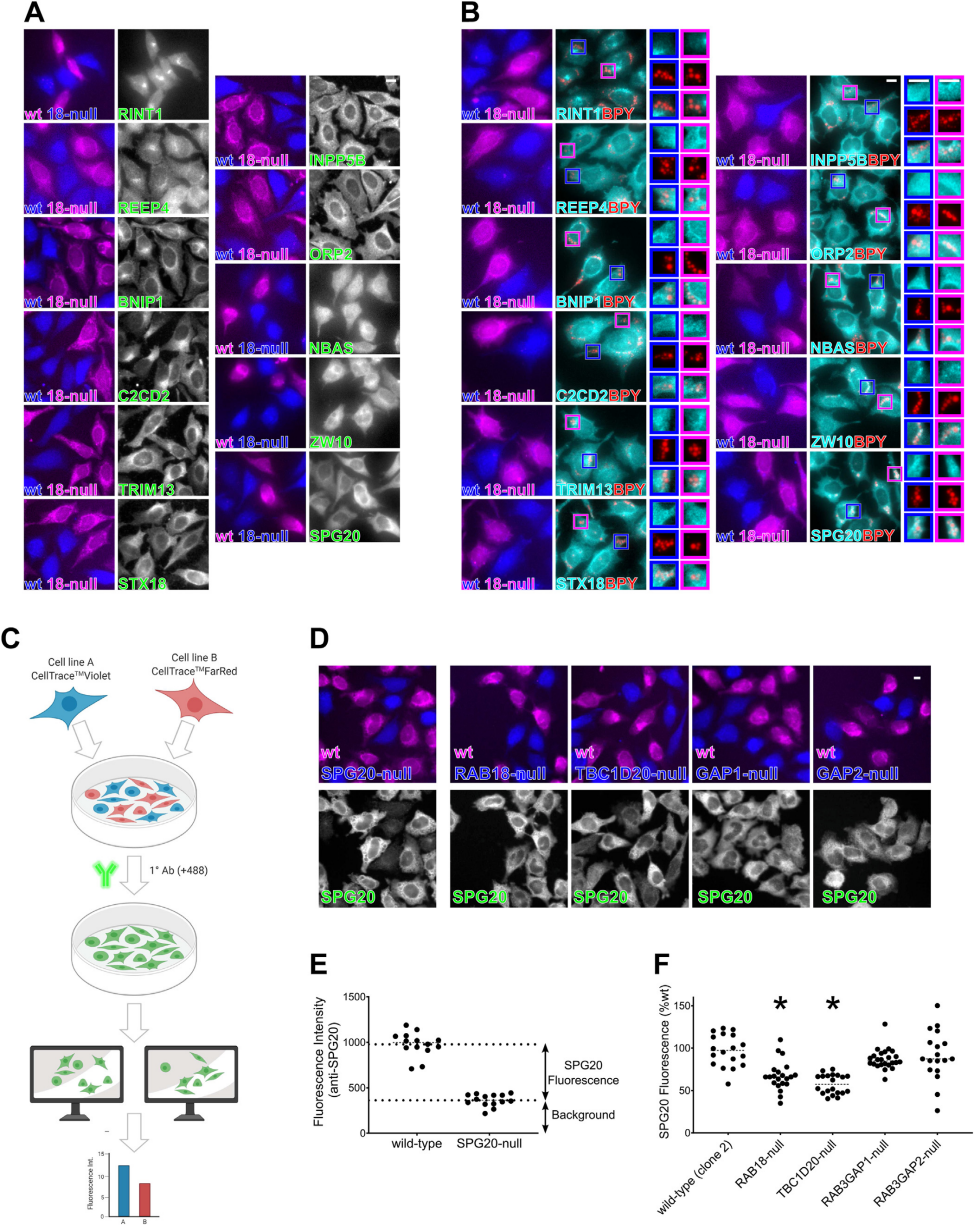
**Initial screening of putative RAB18 effectors reveals that levels of SPG20 are significantly reduced in RAB18-null and TBC1D20-null cells.***A*, comparative fluorescence microscopy of selected RAB18-associated proteins in WT and RAB18-null HeLa cells. Cells of different genotypes were labeled with CellTrace-Violet and CellTrace-Far Red reagents, corresponding to *blue* and *magenta channels*, respectively. Cells were stained with antibodies against indicated proteins in *green channel* panels (shown in *grayscale*). *B*, comparative fluorescence microscopy of selected RAB18-associated proteins in lipid-loaded WT and RAB18-null HeLa cells. Cells were stained as aforementioned but were treated for 15 h with 200 μM oleic acid, 1 μg/ml BODIPY-558/568-C12 (*red channel*; BPY) prior to fixation. *C*, schematic to show method for quantification of protein levels by fluorescence intensity. In each frame, cell areas for each genotype are generated by thresholding CellTrace channels, intensity of antibody staining is measured for each cell in multiple frames. *D*, example frames showing WT and mutant cells of the indicated genotypes, labeled with CellTrace-Far Red and CellTrace-Violet reagents, respectively, and then stained for SPG20. *E,* quantification of SPG20-specific fluorescence in WT cells by direct comparison with SPG20-null cells. *F,* quantification of SPG20 fluorescence (%wt) in cells of different genotypes. Data were derived from analysis of at least 18 frames—each containing >5 WT and >5 mutant cells—per genotype. Two-tailed unpaired Welch’s *t* test ∗*p* < 0.001. Bars represent 10 μm.

Given the suggestive convergences in protein function and gene disease associations, we examined the subcellular localizations of 11 putative effectors for which antibodies were available (Fig. 2, *A* and *B*). For a rapid qualitative assessment of localizations, we employed automated epifluorescence microscopy. The majority of antibodies used were validated in prior studies or produced bands of the expected sizes when used in Western blotting (Table S7). However, antibodies for RINT1, C2CD2, and TRIM13 had only previously been manufacturer validated. To determine whether the localizations of the putative effectors were appreciably altered in cells lacking RAB18, we analyzed WT and RAB18-null lines in each case. In order to directly compare cells of different genotypes under otherwise identical conditions, we labeled them with CellTrace-Violet and CellTrace-Far Red reagents before seeding, immunostaining, and imaging them together. Since RAB18 can localize to LDs, we analyzed both untreated cells (Fig. 2*A*) and cells loaded with oleic acid and labeled with the LD marker BODIPY-558/568-C12 (Fig. 2*B*).

The putative effector proteins showed various staining patterns. These ranged from staining that was enriched at the perinuclear region of cells, to staining that appeared reticular, to staining that appeared more diffuse. Staining patterns were similar in the HeLa cells and also in RPE1 cells generated to provide biological replicates (Fig. S4*A*). Each pattern was compatible with the known localization of RAB18, which is distributed between *cis*-Golgi, ER, and cytosolic compartments (10). In lipid-loaded cells, localizations of proteins with reticular staining patterns overlapped with LDs, but they did not obviously shift to adopt a predominantly LD localization. Two proteins that showed diffuse staining patterns in untreated cells—ZW10 and SPG20—appeared enriched in the vicinity of LDs (Fig. 2*B*, *bottom right panels*).

We saw no evidence for dramatic changes in protein localizations in RAB18-null cells as compared with their WT counterparts. Fluorescence intensities in RAB18-null and WT cells were also generally similar, except in the case of staining for SPG20, which appeared lower in RAB18-null HeLa cells than in WT cells (Fig. 2*A*, *bottom right panels*).

To confirm the reduction in SPG20 fluorescence we observed in the RAB18-null HeLa cells, and to determine the effects of other genotypes, we used quantitative fluorescence microscopy (Fig. 2*C*). To establish SPG20 antibody specificity, we first analyzed SPG20-null cells (Fig. 2*D*, *left panels*). Measured background fluorescence intensity of these SPG20-null cells also provided a baseline level, above which fluorescence levels reflect the presence of SPG20 protein (Fig. 2*E*). In RAB18-null cells, SPG20 fluorescence was reduced to 67.16 ± 17.3% SD (*p* < 0.001) of that in WT cells (Fig. 2*F*). Loss of the RAB18–GEF subunits RAB3GAP1 or RAB3GAP2 had no significant effect, whereas loss of the RAB18–GAP TBC1D20 led to a reduction comparable to that seen in RAB18-null cells (57.48 ± 11.48% SD, *p* < 0.001) (Fig. 2*F*).

We analyzed levels of SPG20 in the corresponding panel of RPE1 cell lines using LFQP analysis of whole-cell lysates (Fig. S4*B* and Table S3). Levels of SPG20 were significantly reduced in RAB18- and TBC1D20-null RPE1 cells compared with WT controls (*p* < 0.05 following false discovery rate correction) but not in the other genotypes tested. SPG20 was one of only 8 of 2017 proteins with significantly altered levels in the RAB18-null cells and 15 of 2017 with significantly altered levels in the TBC1D20-null cells. These data suggest that these genotypes cause reduced SPG20 levels and that this is not the result of clonal variation. A comparison between LFQP data from WT and TBC1D20-null RPE1 and HeLa cells (Tables S3 and S4) showed limited overlap between differentially expressed proteins. This indicates that reduced SPG20 levels are unlikely to have resulted from widespread dysregulation of proteostasis. The RAB18–SPG20 interaction has been previously reported and validated (18), and our findings (aforementioned) provide further support for a physiological relationship between these proteins.

### SEC22A associates with RAB18, and its knockdown causes altered LD morphology

Our screen for RAB3GAP-dependent RAB18 interactors identified all the NRZ complex components as well as the SNARE proteins STX18 and BNIP1 (Table 1). Interestingly, we did not identify SEC22B but did identify SEC22A among these proteins. Although part of the canonical STX18 SNARE complex, SEC22B has been reported to be dispensable for the function of RAB18 at ER–LD contacts (17). SEC22A is one of the two SEC22B homologs in humans that lack the central coiled-coil SNARE domain through which SEC22B mediates membrane fusion (30). Since it had not been previously described as a RAB18-interacting protein, we investigated this further.

**Figure 3:**
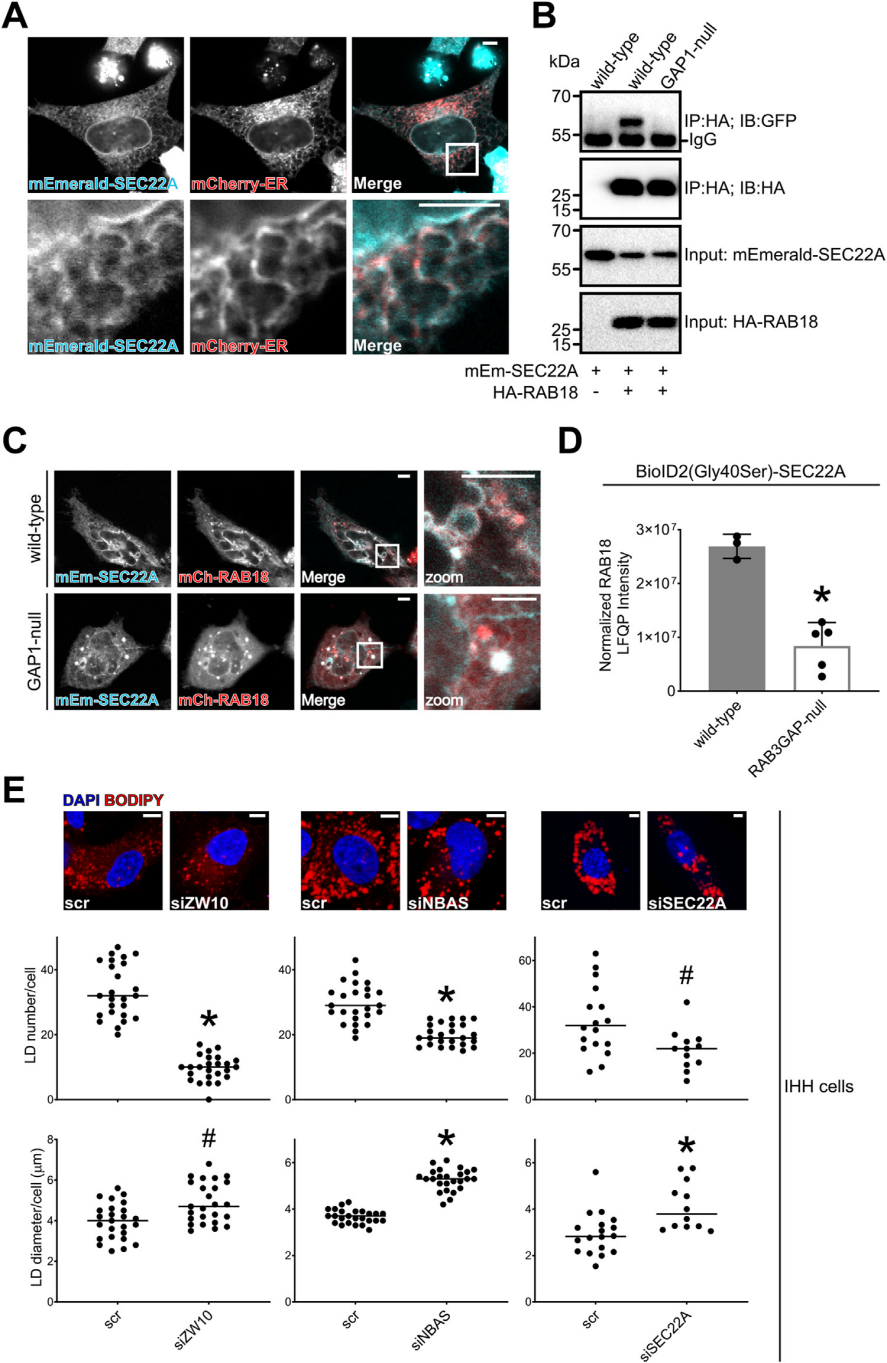
**SEC22A associates with RAB18 and influences lipid droplet (LD) morphology.***A*, confocal micrograph to show overlapping localization of exogenous mEmerald-SEC22A (*cyan*) and mCherry-ER (*red*) in HeLa cells. Images are representative of at least 40 cells in three independent experiments. *B*, immunoprecipitation of exogenous HA-RAB18 from WT and RAB3GAP1-null HeLa cells. Cells were transfected with HA-RAB18 and/or mEmerald-SEC22A and lysed 24 h post-transfection. Anti-HA immunoprecipitates and input samples were subjected to SDS-PAGE and immunostaining for HA and GFP (mEmerald). *C*, confocal micrographs showing altered morphology in WT and RAB3GAP1-null HeLa cells coexpressing mEmerald-SEC22A and mCherry-RAB18; zoom shows colabeled vesicular structures. Images are representative of at least 10 cells in two independent experiments. *D,* RAB18 LFQ intensities from a reciprocal BioID experiment showing a reduced association between BioID2(Gly40Ser)-SEC22A and endogenous RAB18 in RAB3GAP-null compared with WT HeLa cells. Data were adjusted to account for nonspecific binding of RAB18 to beads and normalized by SEC22A LFQ intensities in each replicate experiment. Error bars represent SD. Data for other BioID2(Gly40Ser)-SEC22A-associated proteins are provided in Table S5. *E,* example of confocal micrographs and scatter plots to show effects of ZW10, NBAS, and SEC22A knockdowns on LD number and diameter. siRNA-treated IHH cells were loaded with 200 nM BSA-conjugated oleate, fixed and stained with BODIPY and DAPI, and imaged. Images were analyzed using ImageJ. Data are representative of three independent experiments. Two-tailed unpaired Welch’s *t* test ^#^*p* < 0.05 and ∗*p* < 0.005. Bars represent 5 μm. BSA, bovine serum albumin; DAPI, 4′,6-diamidino-2-phenylindole; ER, endoplasmic reticulum; HA, hemagglutinin; IHH, immortalized human hepatocyte; LFQ, label-free quantitation.

In the absence of appropriate commercially available antibodies for SEC22A, we examined its localization through expression of an mEmerald-SEC22A fusion protein (Fig. 3*A*). mEmerald-SEC22A produced a characteristic reticular staining pattern and colocalized with an exogenous ER marker suggesting that SEC22A localizes to the ER. To verify the RAB18–SEC22A interaction, we carried out immunoprecipitation experiments after exogenous expression of mEmerald-SEC22A and/or hemagglutinin (HA)-RAB18 fusion proteins (Fig. 3*B*). mEmerald-SEC22A copurified together with HA-RAB18 in precipitates from WT but not RAB3GAP1-null cells. These data are consistent with an RAB3GAP-dependent interaction between RAB18 and SEC22A. However, we found that coexpression of mEmerald-SEC22A and mCherry-RAB18 disrupted normal ER morphology and produced vesicular structures and/or inclusions positive for both proteins in both WT and RAB3GAP-null cells (Fig. 3*C*). Although not inconsistent with a functional protein–protein interaction, this precluded the use of coexpressed exogenous proteins in continued testing.

As another means of assessing SEC22A interactions, we used proximity biotinylation with a BirA∗-SEC22A fusion protein in the HeLa cell panel. To minimize potential toxicity while increasing biotin–ligase activity, we used BioID2 (31) with a p.Gly40Ser active site modification (32) and reduced biotin incubation time. Despite a low level of BioID2(Gly40Ser)-SEC22A expression, the construct appeared to label RAB18 in an RAB3GAP-dependent manner (the labeling was reduced in RAB3GAP-null cells) (Fig. 3*D*). About 55 SEC22A-associated proteins were present in samples from WT cells in more than two replicate experiments and represented by more than three unique peptides (Table S5). Furthermore, a subset of nine SEC22A associations were attenuated (intensity ratios <0.5) in samples from both RAB18-null and RAB3GAP-null cells.

A phenotype of altered LD morphology in lipid-loaded cells has been widely reported in cells deficient in RAB18 (8, 9, 17, 25, 33, 34). Similar observations have been made in cells deficient in some components of the NRZ or Syntaxin18 complexes but not in cells deficient in SEC22B (17). To test whether SEC22A expression influences LD morphology, we examined the effects of its silencing in oleic acid–loaded immortalized human hepatocyte (IHH) cells (Fig. 3*E*). ZW10 and NBAS silencing provided positive controls in our experiments. ZW10 and NBAS silencing each led to a significant reduction in LD number (*p* < 0.005) compared with controls and a significant increase in LD size (*p* < 0.05 and *p* < 0.005, respectively). The effects of SEC22A silencing mirrored these findings, producing a significant reduction in LD number (*p* < 0.05) and a significant increase in LD size (*p* < 0.005). Together, these data implicate SEC22A as involved in the same RAB18-mediated process(es) as the NRZ and SNARE proteins.

### RAB18 recruits the orphan lipase TMCO4 to the ER membrane in an RAB3GAP-dependent manner

**Figure 4:**
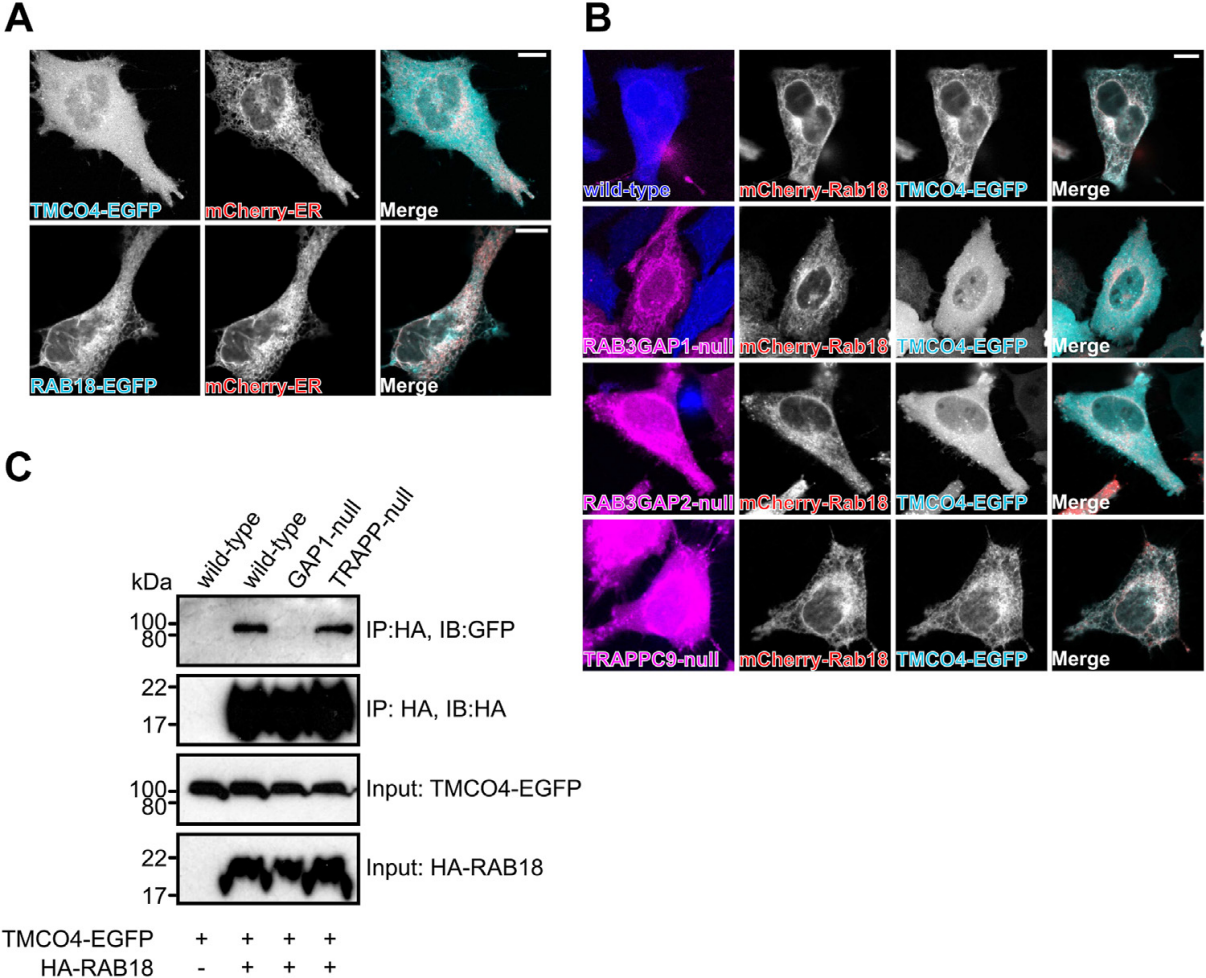
**mCherry-RAB18 recruits TMCO4-EGFP to the ER membrane in an RAB3GAP-dependent manner.***A*, confocal micrographs to show diffuse localization of exogenous TMCO4-EGFP (*green*) compared with mCherry-ER (*red*) and overlapping localization of exogenous EGFP-RAB18 (*green*) and mCherry-ER in HeLa cells. Images are representative of at least 10 cells in two independent experiments. *B*, confocal micrographs to show localization of exogenous mCherry-RAB18 and TMCO4-EGFP in WT cells and in mutant cells of different genotypes. WT and mutant cells of the indicated genotypes were labeled with CellTrace-Violet and CellTrace-Far Red reagents, respectively (*magenta* and *blue channels*). Images are representative of at least 30 cells in two independent experiments. Clear colocalization between mCherry-RAB18 and TMCO4-EGFP was observed in all WT and TRAPPC9-null cells and in no RAB3GAP1- or RAB3GAP2-null cells. *C*, immunoprecipitation of exogenous HA-RAB18 from HeLa cells of different genotypes. Cells were transfected with the indicated constructs and lysed 24 h post-transfection. Anti-HA immunoprecipitates and input samples were subjected to SDS-PAGE and immunostaining for HA and GFP. Bars represent 10 μm. EGFP, enhanced GFP; ER, endoplasmic reticulum; HA, hemagglutinin.

The most novel group of putative RAB18 effectors identified in our study were the lipid-modifying/mobilizing proteins, none of which had been reported to associate with RAB18 previously. Among these, TMCO4 was identified in all three replicate experiments, and its association with RAB18 was highly RAB3GAP dependent (intensity ratio of 0.06). Interestingly, *in silico* analysis suggests that it may be a component of the KICSTOR complex, which is involved in amino acid sensing (35, 36). Although annotated as containing transmembrane and coiled-coil domains, it is orthologous to the yeast protein Mil1/Yfl034w and likely to be a partly soluble lipase (37). Consistently, TMCO4-enhanced GFP (EGFP) expressed in HeLa cells showed a diffuse localization. In contrast, EGFP–RAB18 partly localizes to the ER, as shown by its colocalization with an ER marker (Fig. 4*A*).

To assess the potential RAB18–TMCO4 interaction, we coexpressed mCherry-RAB18 and TMCO4-EGFP (Fig. 4*B*). As in our previous experiments, CellTrace reagents were used to distinguish cells of WT and mutant genotypes. In WT HeLa cells, coexpression of mCherry-RAB18 led to a dramatic redistribution of TMCO4-EGFP to the ER membrane suggesting that RAB18 mediates recruitment of TMCO4 to this compartment. Redistribution was completely absent in RAB3GAP1- and RAB3GAP2-null cells but unaffected in TRAPPC9-null cells, consistent with the BioID data.

As a means of verifying the RAB18–TMCO4 interaction, we carried out immunoprecipitation experiments using exogenous HA-RAB18 and TMCO4-EGFP (Fig. 4*C*). As expected, TMCO4-EGFP copurified with HA-RAB18 when expressed in WT or TRAPPC9-null cells but not when expressed in RAB3GAP1-null cells. These data indicate that RAB18 and TMCO4 interact directly or indirectly as part of a protein complex in an RAB3GAP-dependent manner. Furthermore, both the microscopy and the immunoprecipitation data support the suggestion that different GEFs can promote different RAB18 interactions.

### RAB18 is involved in cholesterol mobilization and biosynthesis

Other putative RAB18 effectors with lipid-related functions included ORP2/OSBPL2, INPP5B, and EBP. Of these, ORP2 (38) has been implicated in many of the same cellular processes as RAB18. Both are enriched at ER–LD MCSs upon lipid loading (17, 23, 24, 39, 40, 41). Both regulate lipolysis (22, 24, 39, 40). Both are linked to COPI subunits and ATGL/PNPLA2 (25, 40, 42). Indeed, both in addition have proposed roles in regulating focal adhesions and cell adhesion (43, 44, 45). Moreover, ORP2 is suggested to regulate LXR signaling whilst RAB18 expression is suggested to be responsive to it (46, 47). At a molecular level, ORP2 and INPP5B are robustly linked to a role in cholesterol mobilization. ORP2 is thought to function as a lipid transfer protein that exchanges cholesterol and PI(4,5)P_2_ (48). INPP5B is implicated in the hydrolysis of PI(4,5)P_2_, presumably driving the exchange process (48). Consistently, several prior studies showed that ORP2 overexpression enhances cholesterol efflux in Chinese hamster ovary (CHO) and HeLa cells. In the HeLa cells, cholesterol esterification is also enhanced, whilst in the CHO cells, it is reduced (49, 50). On the basis of these findings, we investigated the potential role of RAB18 in cholesterol uptake and efflux.

**Figure 5:**
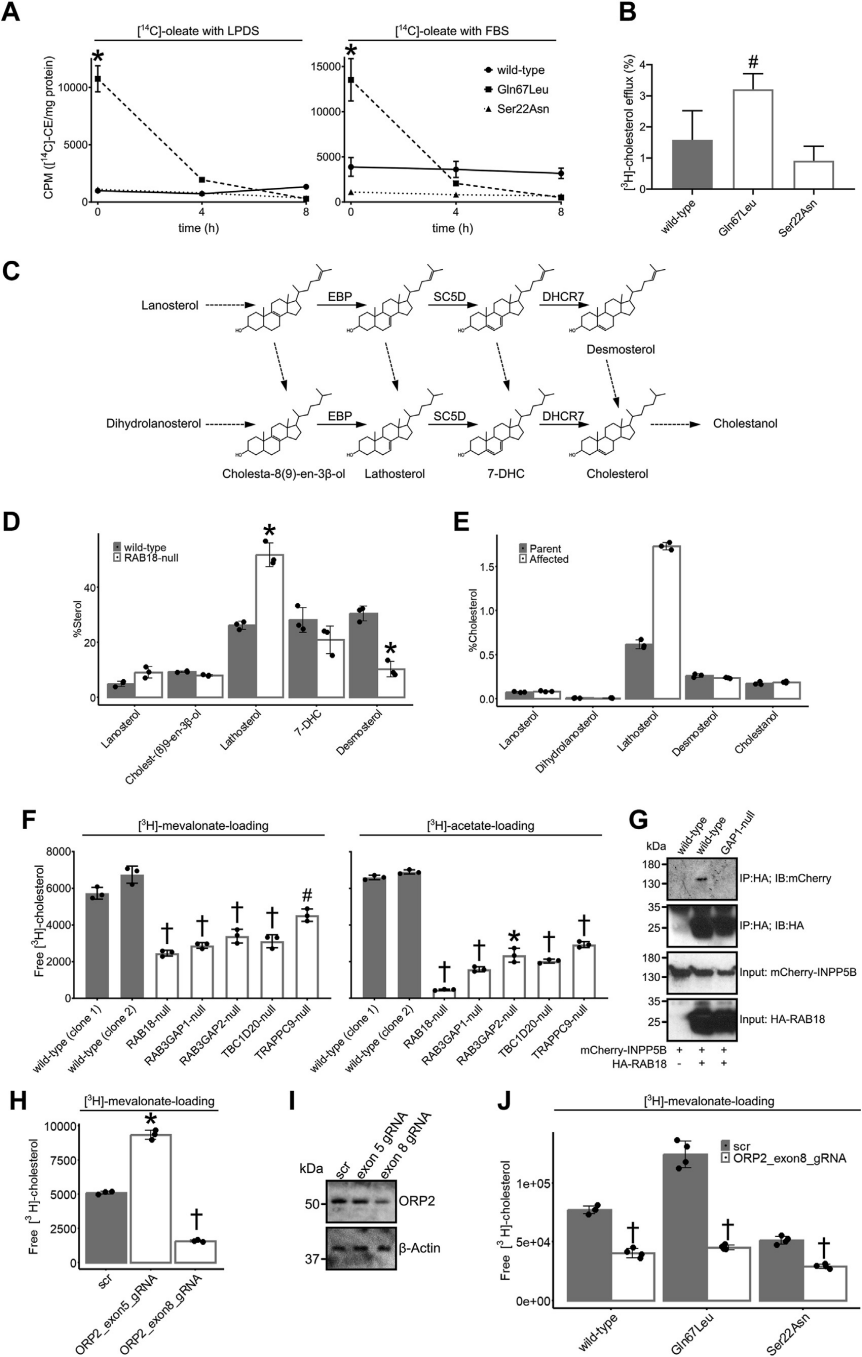
**RAB18 is involved in the mobilization and biosynthesis of cholesterol.***A*, plots to show cholesteryl ester (CE) loading and efflux. CHO cells, stably expressing RAB18(WT), RAB18(Gln67Leu), and RAB18(Ser22Asn), were incubated with [^14^C]-oleate, for 24 h, in the presence of lipoprotein-depleted serum (LPDS) (*left panel*) or FBS (*right panel*). Following lipid extraction, TLC was used to separate CE, and radioactivity was measured by scintillation counting. Measurements were made at *t* = 0 and at 4 and 8 h following the addition of 50 μg/ml high-density lipoprotein (HDL) to the cells. *B*, bar graph to show cholesterol efflux. CHO cells were incubated with [^3^H]-cholesterol, for 24 h, in the presence of FBS. After washing, they were incubated with 25 μg/ml apolipoprotein A-I for 5 h. The quantity of [^3^H]-sterol in the media is shown as a percentage of the total cellular radioactivity (mean ± SD). *C*, schematic of postsqualene cholesterol biosynthesis pathway with the sterols quantified by GC–MS–selected ion monitoring (GC–MS–SIM) named. *Solid arrows* indicate biosynthetic steps catalyzed by EBP, SC5D, and DHCR7. *D,* bar graph of sterol profile in WT and RAB18-null HeLa cells. Cells were grown in media supplemented with LPDS for 48 h. Extracted sterols were analyzed by GC–M–SIM. Percent of sterol was calculated as a proportion of total quantified sterols, excluding cholesterol, following normalization to a 5α-cholestane internal standard. n = 3; ±SD. *E,* bar graph of sterol profile in parental control fibroblasts and RAB3GAP1-deficient fibroblasts from an individual with Micro syndrome. Cells were grown in media supplemented with LPDS for 48 h. Extracted sterols were analyzed by GC–MS–SIM. Percent of cholesterol was calculated to express each quantified sterol as a proportion of total quantified cholesterol. *F,* bar graphs to show incorporation of [^3^H]-mevalonate and [^3^H]-acetate into cholesterol in a panel of HeLa cell lines. Cells were grown in media supplemented with LPDS for 24 h and then incubated with 5 μCi/well [^3^H]-mevalonate or 10 μCi/well [^3^H]-acetate for 24 h. TLC was used to separate free cholesterol, and radioactivity was quantified by scintillation counting (n = 3; mean ± SD). *G,* immunoprecipitation of HA-RAB18 from HeLa cells of different genotypes. Cells were transfected with the indicated constructs and lysed 24 h post-transfection. Anti-HA immunoprecipitates and input samples were subjected to SDS-PAGE and immunostaining for HA and mCherry. *H,* bar graph to show incorporation of [^3^H]-mevalonate into cholesterol in HEK293 cells transduced with lentivirus constructs. Cells transduced with the indicated constructs were selected with puromycin for at least 7 days, grown in media supplemented with LPDS for 24 h, and then incubated with 5 μCi/well [^3^H]-mevalonate for 24 h. TLC was used to separate free cholesterol, and radioactivity was quantified by scintillation counting (n = 3; mean ± SD). *I,* Western blotting to show levels of full-length OSBPL2 expression in cells transduced with the indicated lentivirus constructs. Prior to sampling, cells were selected with puromycin for at least 7 days. *J,* bar graph to show incorporation of [^3^H]-mevalonate into cholesterol in HEK293 cells stably expressing RAB18(WT), RAB18(Gln67Leu), and RAB18(Ser22Asn) and transduced with lentivirus constructs. Cells transduced with nontargeting (scr) or ORP2 exon 8-targeting CRISPR constructs were selected with puromycin for at least 7 days, grown in media supplemented with LPDS for 24 h, and then incubated with 5 μCi/well [^3^H]-mevalonate for 24 h. TLC was used to separate free cholesterol, and radioactivity was quantified by scintillation counting (n = 4; mean ± SD). Two-tailed unpaired Welch’s *t* test ^#^*p* < 0.05, ∗*p* < 0.01, and ^†^*p* < 0.001. HA, hemagglutinin; HEK293, human embryonic kidney 293 cell line.

We performed loading and efflux experiments to measure the flux of cholesterol/cholesteryl ester (CE) while modifying the activity of RAB18. CHO cells were generated to stably express RAB18(WT), RAB18(Gln67Leu), or RAB18(Ser22Asn) (Fig. S5*A*). Labeled sterols were separated by TLC, with reference to Rf values and cold standards (Fig. S6), and quantified by scintillation counting. In cells labeled with [^14^C]-oleate, but cholesterol depleted with lipoprotein-depleted serum (LPDS), levels of CE were comparable in RAB18(Ser22Asn) and RAB18(WT) cells, whereas RAB18(Gln67Leu) cells stored significantly more (Fig. 5*A*, *left panel*). In cells labeled with [^14^C]-oleate and cholesterol loaded with fetal bovine serum (FBS), levels of CE in RAB18(Ser22Asn) remained unchanged, whereas its storage was elevated in RAB18(WT) cells and RAB18(Gln67Leu) cells (Fig. 5*A*, *right panel*). Interestingly, in both [^14^C]-oleate/LPDS and [^14^C]-oleate/FBS cells, the addition of high-density lipoprotein (a vehicle mediating removal of cellular cholesterol) led to rapid depletion of CE in RAB18(Gln67Leu) cells but not in RAB18(Ser22Asn) or RAB18(WT) cells (Fig. 5*A*). Consistently, RAB18(Gln67Leu) cells also effluxed significantly more [^3^H]-sterol upon their incubation with apolipoprotein (apo) A-I than the other cell types (Fig. 5*B*). These observations were not explained by altered expression of ABCA1, the transporter responsible for the rate-limiting step of cholesterol efflux (Fig. S5*B*). These data suggest that “activated” GTP-bound RAB18 strongly promotes the storage, turnover, and mobilization of CE stored in LDs. A plausible explanation for this is that active RAB18 promotes cholesterol mobilization *via* ORP2 and INPP5B. The effects of active RAB18 on sterol efflux are similar to those of ORP2 overexpression. Furthermore, although ORP2 overexpression in CHO cells reduces cholesterol esterification rather than increasing it, the fact that it can enhance both cholesterol efflux and esterification in HeLa cells suggests its involvement in bidirectional transport (49, 50).

EBP is involved in *de novo* cholesterol biosynthesis (51). In the Bloch pathway, it catalyzes the conversion of 5α-cholesta-8, 24-dien-3β-ol (zymosterol) to 5α-cholesta-7, 24-dien-3β-ol (24-dehydrolathosterol). In the Kandutsch–Russel pathway, it catalyzes the conversion of 5α-cholest-8(9)-en-3β-ol to 5α-cholest-7-en-3β-ol (lathosterol) (52). Given this role, we next explored whether the absence of putative RAB18 reglulation of EBP might produce abnormal sterol profiles. A schematic of the cholesterol biosynthetic pathway is shown in Figure 5*C*. We incubated WT and RAB18-null HeLa cells for 48 h in media supplemented with LPDS and then subjected samples to analysis by GC–MS–SIM (selected ion monitoring) (Fig. 5*D*). Representative labeled chromatograms are shown in Fig. S7, *A* and *B*. In RAB18-null cells, we found that levels of the EBP substrate cholest-8(9)-en-3β-ol were not significantly different from those in WT cells. In contrast, levels of EBP-product lathosterol were significantly higher (*p* < 0.01). Moreover, levels of desmosterol—downstream of 24-dehydrolathosterol in the Bloch pathway—were significantly lower in RAB18-null cells (*p* < 0.01). These differences remain significant when the data are calibrated to reflect absolute levels of sterols rather than detected ions (Fig. S7, *C* and *D*).

We extended our sterol profiling with additional experiments using RAB3GAP1-deficient primary fibroblasts from an individual with Micro syndrome together with control cells derived from a parent (Fig. 5*E*). Representative labeled chromatograms are shown in Fig. S8. Following culturing with LPDS, as in the HeLa cells, we found that lathosterol levels were higher in the RAB3GAP1-deficient fibroblasts than in the control cells. More testing of fibroblasts from additional control and Micro syndrome individuals will be required to exclude the possibility that altered lathosterol levels result from interindividual differences other than disease status. Nevertheless, the finding that levels of the same specific sterol are elevated in a second cell type, deficient in a second Micro syndrome gene, provide good evidence that the RAB18–EBP interaction identified in our screening is meaningful.

### ORP2 is required for RAB18-mediated cholesterol biosynthesis

We next reasoned that the elevated levels of a cholesterol precursor we observed in the RAB18-null and RAB3GAP1-deficient cells might be reflected in altered cholesterol biosynthesis. In particular, that the elevated lathosterol levels might reflect its accumulation because of perturbed transit through the biosynthetic pathway. To explore this possibility, we cultured the panel of HeLa cell lines for 24 h in media supplemented with LPDS, treated them for 24 h with [^3^H]-mevalonate or [^3^H]-acetate, and then quantified labeled cholesterol (Fig. 5*F*). Under both conditions, cholesterol synthesis in two clonal WT controls was comparable, but it was reduced in RAB18-, RAB3GAP1-, RAB3GAP2-, TBC1D20-, and TRAPPC9-null cells. Levels of newly produced cholesterol were lowest in the RAB18-null cells (39.5 ± 2.5% SD and 6.8 ± 0.5% SD of controls for [^3^H]-mevalonate and [^3^H]-acetate, respectively). Levels in the cells of other genotypes were between 46 ± 2.5% to 73 ± 5% SD for [^3^H]-mevalonate and 23 ± 2% to 43 ± 2% SD for [^3^H]-acetate. These data strongly suggest that RAB18 and its regulators are required for normal cholesterol biosynthesis.

We attempted more direct verification of the putative interactions between RAB18 and ORP2, EBP and INPP5B using exogenously expressed fusion proteins and immunoprecipitation and GFP-Trap experiments. We were able to verify an RAB3GAP-dependent interaction between HA-RAB18 and mCherry-INPP5B using immunoprecipitation (Fig. 5*G*). However, we did not detect the interactions with EBP or ORP2, perhaps indicating that these are transient, weak, disrupted by the lysis conditions or tags used, or involve alternative protein isoforms.

Several lines of evidence support the existence of different protein isoforms of ORP2. At a functional level, these include differing findings from various knockout and knockdown models (Table S6). Most strikingly, knockout mice generated through deletion of sequence encoding exons 3 and 4 of the canonical transcript exhibit hearing loss whilst mice generated through the introduction of frameshift variants into exon 3 do not (53, 54). More obliquely, HeLa and Hep2G cells in which exon 2 is edited with small deletions have increased cholesterol (40, 55). In contrast, HEK293 cells in which the exon 2 splice-donor site is probably excised show evidence for cholesterol insufficiency, including reduced DH4-accessible plasma membrane cholesterol, sensitivity to lipoprotein depletion in media, and impaired proliferation improved by cholesterol supplementation (48). Broadly, the findings of increased cholesterol are consistent with one set of models (47, 56, 57, 58), whilst the findings of reduced accessible cholesterol are consistent with another (45, 59).

Various human ORP2 transcripts are annotated on Ensembl, including four with unique CCDS (consensus CDS protein set) entries (Ensembl release 109, CCDS release 24). Northern blotting supports the existence of two major transcripts and three additional transcripts (60), but publicly available long-read sequencing data are not yet of sufficient depth to accurately ascertain their precise identity and the extent of their contribution to gene expression. In human and mouse, cap analysis of gene expression data indicates that transcription begins at a single site or closely adjacent sites within a CpG island (Fig. S9), and there is little indication of prominent transcription start sites elsewhere at the locus.

Different protein isoforms can result from regulation at the level of translation as well as regulation at the level of alternative splicing (61, 62, 63). Interestingly, ribosome profiling data for ORP2 provide evidence for such translational control. In human, mouse, and zebrafish, substantial translation occurs upstream of the Met1 codon in a region of the 5′-UTR corresponding to short upstream ORFs (Fig. S10). These may regulate translation initiation at the main ORP2 ORF. In human and mouse, profiling data employing chemical treatments to identify translation initiation sites identify potential initiation downstream of the annotated canonical start codon (Fig. S11). In humans, one site is at Met33, with a corresponding site observed nearby in mouse. An ORP2 protein translated from these sites would lack the VAP-interacting FFAT motif and have an approximate molecular weight of 52 kDa. Consistent with its being expressed physiologically, a 51 kDa ORP2 band is observed upon Western blotting of cells transfected with human ORP2 complementary DNA and also upon blotting of a panel of mouse tissues (49).

Consistent with possible translation initiation further downstream, human ORP2 transcripts containing exon 3 frameshift variants are unusually not subject to nonsense-mediated decay. Levels of ORP2 mRNA are unchanged in individuals harboring these variants, and the expression of truncated protein products is proposed to contribute to autosomal dominant pathophysiology (54). Furthermore, one ORP2 splice variant, NM_001278649.3 (CCDS63323), has an annotated translation-initiation site at an AUG that is in exon 5 of the canonical transcript. NM_001278649.3 contains a unique exon for which low-level ribosome profiling signal provides evidence of both physiological expression and ribosome engagement (Fig. S12, *A* and *B*).

Clearly, if nonsense-mediated decay does not necessarily degrade frameshift-containing ORP2 transcripts, then the effects of frameshift-inducing CRISPR gene editing may not be straightforward. One explanation for the different phenotypes of different knockout animals (53, 54), and exon 2-targeted CRISPR cell lines (40, 48, 55), could be differential effects on the regulation of alternative ORP2 isoforms initiating from alternative start codons.

To independently determine the effects of ORP2 disruption on cholesterol biosynthesis, we generated lentivirus particles for the expression of Cas9 together with guide RNAs that were nontargeting (scr) or targeted exon 5 or exon 8 of the *OSBPL2* gene. HEK293 cells were transduced with these particles, and then transduced cells were selected with puromycin. The cells were cultured in media supplemented with LPDS and treated with [^3^H]-mevalonate for 24 h, and then labeled cholesterol was quantified by TLC. Surprisingly, we found that when exon 5 was targeted, cholesterol synthesis was significantly increased (to 184 ± 7% SD scr), whereas when exon 8 was targeted, cholesterol synthesis was significantly reduced (to 31 ± 1% SD scr) (Fig. 5*H*). We cannot fully account for these data but note that apart from the exon 2-targeted cell lines discussed previously, all other CRISPR-generated ORP2 models associated with increased cholesterol also target exon 5 (Table S6 and Fig. S12*C*).

To establish which targeting construct was most likely to reflect the effects of introduction of null alleles, we carried out additional experiments in which cells were transduced with the exon 5- and exon 8-targeting constructs simultaneously (Fig. S13). The phenotypic effects of null alleles would be expected to predominate under dual transduction since further disruption of a functional allele might produce a null, whereas further disruption of a null allele would be unlikely to restore function. We found that cholesterol synthesis was significantly reduced in the dual-transduced cells and to a comparable extent to that seen with the exon 8-targeting construct alone (Fig. S13*A*). Therefore, we conclude that the exon 8-targeting construct is most likely to cause absent or reduced gene expression, whereas the exon 5-targeting construct is most likely to produce altered gene expression. Consistently, the exon 8-targeting construct also produces a greater apparent reduction in full-length protein expression than the exon 5 construct (Figs. 5*I* and S13*B*).

To determine whether ORP2 is involved in the RAB18-mediated regulation of cholesterol biosynthesis, we generated HEK293 cell lines stably expressing WT RAB18, RAB18 (Gln67Leu), and RAB18 (Ser22Asn). We then transduced these with either nontargeting or OSBPL2 exon 8-targeting lentivirus and analyzed cholesterol biosynthesis as before (Fig. 5*J*). We found that compared with cells expressing WT RAB18, cholesterol biosynthesis was increased in the RAB18(Gln67Leu) cells in which RAB18 is constitutively active (to 162 ± 15% SD WT), whilst it was reduced in the RAB18(Ser22Asn) cells in which it is constitutively inactive (to 67 ± 4% SD WT). These data further support the involvement of RAB18 in the cholesterol biosynthesis. We found that transduction with the OSBPL2 exon 8-targeting lentivirus significantly reduced cholesterol biosynthesis in each cell line, with synthesis in both WT RAB18- and RAB18(Gln67Leu)-expressing cells reduced to a comparable baseline level. These data suggest that RAB18-mediated cholesterol biosynthesis requires ORP2.

## Discussion

In this study, we have complemented previous work showing that proximity biotinylation is a powerful means of identifying candidate Rab effectors (13). Furthermore, at least in the case of RAB18, we have found that comparing biotin labeling produced by a BirA∗-Rab in WT and GEF-deficient cells can be particularly informative. We found that marked reductions in RAB18 association in RAB3GAP-null cells were restricted to only 28 proteins and that these comprised known and/or plausible effectors. We were able to exclude ∼94% of RAB18 associations from consideration as more likely to represent “noise” from bystander proteins.

We anticipate that our approach could be readily applied in the study of other GTPases with known GEFs. In support of its specificity and sensitivity, prior evidence identified 12 of the 28 interactions we detected. Independent experiments with a mutant RAB18 fusion protein confirmed nucleotide binding dependence of several interactors, and immunofluorescence confirmed compatible localizations of several more. The known functions of the proteins were consistent with previous work implicating RAB18 in coordination of lipid exchange between apposed membranes (17). Furthermore, gene-disease associations showed substantial overlap with RAB18 deficiency/Warburg Micro syndrome. We have presented additional validation by immunoprecipitation of novel interactions with SEC22A, TMCO4, and INPP5B (Figs. 3*B*, 4*C*, and 5*G*). Our more indirect data are consistent with functional interactions between RAB18 and its known interactor SPG20 (Figs. 2, *D*–*F* and S4*B*) and novel putative interactors ORP2 and EBP (Fig. 5, *A*–*F*, *H*, and *J*).

Together, our protein-interaction data implicate RAB18 in regulation of a stepwise process in which membrane/cytoskeletal remodeling precedes the engagement of tethering proteins and then SNAREs to establish MCSs. The possible substitution of SEC22B for SEC22A in an RAB18-regulated Syntaxin18 SNARE complex, and a possible role for this complex in promoting membrane contacts rather than membrane fusion, is consistent with previous data (17). Furthermore, it would be compatible with roles for the NRZ–Dsl1 complex and SCFD2/Sly1 in dynamically orchestrating SNARE complex assembly (64, 65, 66). More ambiguously, the RAB18-interacting microtubule-binding proteins have not previously been reported to work together but do function in compatible locations. SPG20 and CAMSAP1 each associate with mitotic spindle poles, REEP4 participates in spindle-dependent ER clearance from metaphase chromatin, and BICD2 is a component of the minus-end-directed dynein–dynactin motor complex (67, 68, 69, 70, 71, 72, 73). Our TRAPPII-dependent RAB18 interaction data indicate that different GEF complexes affect largely distinct subsets of interactions. However, more work will be required to determine whether these regulators mediate independent or interdependent functions.

Proteins implicated in lipid biology—particularly sterol biology—were prominent in our dataset. Consistent with the putative interaction between RAB18 and novel effectors ORP2 and INPP5B, which are reported to function in cholesterol mobilization (48, 49, 50), altered RAB18 activity was associated with altered cholesterol/CE mobilization in our experiments (Fig. 5, *A* and *B*). Other research implicates interactions between other Rab and ORP/OSBP isoforms in cholesterol mobilization at discrete sites (74, 75), and several INPPs including INPP5B have a broad Rab-binding specificity (12, 13, 76). Thus, there may be a conserved relationship between these protein families, functioning in an analogous manner to the ARF1 GTPase, OSBP, and the phosphatase SACM1L (SAC1), in mediating sterol exchange (77).

Consistent with a functional interaction between RAB18 and EBP, levels of the EBP-product lathosterol were elevated in RAB18-null HeLa cells and in RAB3GAP1-deficient human primary fibroblasts (Fig. 5, *D* and *E*). Cholesterol biosynthesis in HeLa cells was impaired when RAB18 was absent or dysregulated (Fig. 5*F*). Moreover, it was also impaired when ORP2 expression was disrupted (Fig. 5, *H*–*J*). Given that ORP2 and INPP5B function in sterol mobilization, whereas EBP functions in sterol biosynthesis, an attractive hypothesis is that RAB18 might coordinate their activities; that ORP2 might act as an exchanger for the products of EBP catalysis as well as for cholesterol (Fig. S14). In this case, defective mobilization of lathosterol would explain its accumulation in RAB18-null cells. Impaired delivery of substrates to downstream biosynthetic enzymes would explain the reduced cholesterol biosynthesis observed in these and the other model cell lines. The mobilization of cholesterol precursors by ORP proteins would not be unprecedented since the mobilization of cholesterol and its metabolites by these proteins is well established. Nevertheless, future work should aim to test this hypothesis more definitively. Important preliminary questions are whether there are different ORP2 protein isoforms and whether these have divergent functions. The varied findings from ORP2 CRISPR models (Fig. 5, *H* and *I* and Table S6) should be explained, and the potential splice and translational regulation of ORP2 transcripts (Figs. S9–S13) should be addressed.

Among the other lipid-related proteins, TMCO4 may potentially be directly or indirectly associated with sterol metabolism. Although its substrate(s) are unknown, its expression is found to be upregulated in hypercholesterolemia (78), and it is present on lipid rafts (79). C2CD2L/TMEM24 and C2CD2 might potentially function in concert with ORP2 and/or INPP5B, since C2CD2L is found to mediate phosphatidylinositol transport and to facilitate generation of PI(4,5)P_2_ (80).

Our objective in studying RAB18 was to better understand the molecular pathology of Warburg Micro syndrome. Though our protein-interaction data are relatively preliminary, our functional findings represent good progress toward this goal. One key finding is that levels of lathosterol are significantly elevated in primary fibroblasts from an affected individual when these cells are cultured under LPDS (Fig. 5*E*). In future work, we aim to determine whether this is reproducible in fibroblasts of other genotypes from other Micro syndrome individuals. If so, this could form the basis for a biochemical test for Micro syndrome, which would complement genetic testing.

Another key finding is that disrupted *de novo* cholesterol biosynthesis may contribute to disease pathogenesis. Strongly supporting this suggestion, genes encoding multiple cholesterol biosynthesis enzymes are linked to similar disorders (52). For example, pathogenic variants in the lathosterol oxidase gene, *SC5D*, cause lathosterolosis, which is associated with microcephaly, intellectual disability, micrognathia, high arched palate, and cataract (81, 82, 83, 84, 85). Pathogenic variants in the 7-dehydrocholesterol reductase gene, *DHCR7*, cause Smith–Lemli–Opitz syndrome, which has a similar spectrum of features and is among the top differential diagnoses for Micro syndrome (7, 86). Indeed, the similarities with Smith–Lemli–Opitz syndrome were noted in the report first identifying RAB18 as a disease-associated gene more than a decade ago (5).

## Experimental procedures

### Plasmids

The EGFP-RAB18 construct has been described previously (9). The RAB18 sequence was excised from this construct using BamHI and HindIII restriction enzymes (New England Biolabs) and used to generate constructs encoding mEmerald-RAB18 and mCherry-RAB18 by ligation into mEmerald-C1 and mCherry-C1 vectors (Addgene) using HC T4 Ligase and rapid ligation buffer (Promega). Constructs encoding BirA∗-RAB18, BioID2(Gly40Ser)-SEC22A, and mEmerald-SEC22A were generated following PCR amplification from template and subcloning into an intermediate pCR-Blunt II-TOPO vector using a Zero Blunt TOPO PCR Cloning Kit (ThermoFisher Scientific) according to the manufacturer’s instructions. Fragments were excised from intermediate vectors and then subcloned into target vectors using restriction ligation, as aforementioned. A construct encoding mCherry-ER was obtained from Addgene, and a construct encoding TMCO4-EGFP was synthesized and cloned by GeneWiz. Generation of recombinant pX461 and pX462 plasmids for CRISPR gene editing and recombinant pCMV vectors for preparation of stable CHO cell lines are described later. Generation of recombinant pcDNA5 FRT/TO FLAG-BirA(Arg118Gly) and pcDNA5 FRT/TO vectors for preparation of stable T-Rex-293 cell lines is described later. Details of other plasmids, PCR templates, primers, and target vectors are listed in Table S7.

### Antibodies and reagents

A custom polyclonal antibody to RAB18 generated by Eurogentec has been described previously (10). An antibody to RAB3GAP1 was obtained from Bethyl Labs, an antibody to GFP was obtained from Takara Bio, an antibody to β-tubulin was obtained from Abcam, and an antibody to β-actin was obtained from ThermoFisher. Antibodies to HA, RAB3GAP2, and TBC1D20 were obtained from Merck. Antibodies to ZW10, STX18, SPG20, RINT1, REEP4, BNIP1, C2CD2, TRIM13, WFS1, INPP5B, OSBPL2, and NBAS were obtained from Proteintech. Antibody catalog numbers and the dilutions used in this study are listed in Table S7.

### Cell culture

HeLa, T-REx-293, HEK293FT, IHH cells, and human fibroblasts were maintained in Dulbecco's modified Eagle's medium (DMEM), RPE1 cells in DMEM/F12 media, and CHO cells in alpha-MEM media (ThermoFisher). In each case, media were supplemented with 10% fetal calf serum and 1% penicillin–streptomycin. Cells were maintained at 37 °C and 5% CO_2_. Human fibroblasts were originally derived from biopsies taken from an unaffected mother and her affected infant daughter. These cells were imported into the United Kingdom as mature cultures. Cell lines were routinely tested for mycoplasma and always found negative. The sources of the cell lines and evidence for their authenticity are given in Table S7.

### Generation of clonal “knockout” HeLa and RPE1 cell lines

CRISPR–Cas9 gene editing was carried out essentially as described in the study by Ran *et al*. (87). Guide RNA (gRNA) sequences are shown in Table S7. A list of the clonal cell lines generated for this study, together with the loss-of-function variants they carry, is shown in Fig. S1*A*. Western blot validation is shown in Fig. S1, *B*–*E*. Briefly, for each targeted exon, pairs of gRNA sequences were selected using the online CRISPR design tool (http://crispr.mit.edu/). Oligonucleotide pairs incorporating these sequences (Sigma) were annealed (at 50 mM ea.) in 10 mM Tris (pH 8), 50 mM NaCl, and 1 mM EDTA by incubation at 95 °C for 10 min followed by cooling to room temperature. Annealed oligonucleotides were diluted and ligated into BbsI-digested pX461 and pX462 plasmids (Addgene) using HC T4 Ligase and rapid ligation buffer (Promega). Sequences of all recombinant plasmids were verified by direct sequencing. Pairs of plasmids were cotransfected into cells using Lipofectamine 2000 reagent according to the manufacturer’s instructions. Cells were selected for puromycin resistance (conferred by pX462) using 24 h puromycin treatment. Following 12 h recovery, they were selected for GFP fluorescence (conferred by pX461) and cloned using FACSAria2 SORP, Influx, or FACSMelody Instruments (BD). After sufficient growth, clones were analyzed by PCR of the targeted exons (primers are listed in Table S7). In order to sequence individual gene-edited alleles, PCR products from each clone were first cloned into ZeroBlunt TOPO vector (ThermoFisher) and then subjected to colony PCR. These PCR products were then analyzed by direct sequencing. Sequencing data were assessed using BioEdit software (http://www.mbio.ncsu.edu/BioEdit/bioedit.html).

### BirA∗/BioID proximity labeling (HeLa cells)

Proximity labeling in HeLa cells was carried out largely as described by Roux *et al*. (15) but with minor modifications. HeLa cells were grown to 80% confluence in T75 flasks, and then each flask was transfected with 1 to 1.5 μg of the BirA∗-RAB18 construct or 1 μg of the BioID2(Gly40Ser)-SEC22A construct using Lipofectamine 2000 reagent in Opti-MEM serum-free medium (ThermoFisher) for 4 h, according to the manufacturer’s instructions. About 24 h post-transfection, media were replaced with fresh media containing 50 μM biotin (Merck), and the cells were incubated for a further 24 or 6 h (for BirA∗-RAB18 and BioID2(Gly40Ser)-SEC22A experiments, respectively). Cells were then trypsinized and washed twice in PBS before pellets were transferred to 2 ml microcentrifuge tubes and snap frozen. For each pellet, lysis was carried out in 420 μl of a buffer containing 0.2% SDS, 6% Triton X-100, 500 mM NaCl, 1 mM DTT, EDTA-free protease-inhibitor solution (Expedeon), and 50 mM Tris (pH 7.4). Lysates were sonicated for 10 min using a Bioruptor device together with protein extraction beads (Diagenode). Each lysate was diluted with 1080 μl 50 mM Tris (pH 7.4), and they were then clarified by centrifugation at 20,000*g* for 30 min at 4 °C. AP of biotinylated proteins was carried out by incubation of clarified lysates with streptavidin-coated magnetic Dynabeads (ThermoFisher) for 24 h at 4 °C. Note that a mixture of Dynabeads—MyOne C1, MyOne T1, M270, and M280—was used to overcome a problem with bead clumping observed when MyOne C1 beads were used alone. Successive washes were carried out at room temperature with 2% SDS, a buffer containing 1% Triton X-100, 1 mM EDTA, 500 mM NaCl, 50 mM Hepes (pH 7.5), a buffer containing 0.5% NP-40, 1 mM EDTA, 250 mM LiCl, 10 mM Tris (pH 7.4), 50 mM Tris (pH 7.4), and 50 mM ammonium bicarbonate.

### MS

Washed beads from BioID experiments with HeLa cells were subjected to limited proteolysis by trypsin (0.3 μg) at 27 °C for 6.5 h in 2 mM urea, 1 mM DTT, 75 mM Tris, pH = 8.5, and then supernatants were incubated overnight at 37 °C. Samples were alkylated with 50 mM iodoacetamide in the dark for 20 min and then acidified by addition of 8 μl 10% TFA. Peptides were generated using trypsin. Trypsin cleaves on the C-terminal side of lysine and arginine residues unless the C-terminal residue is proline. Hydrolysis is slower where the C-terminal residue is acidic. Peptides were loaded on to activated (methanol), equilibrated (0.1% TFA) C18 stage tips before being washed with 0.1% TFA, and eluted with 0.1% TFA/80 acetonitrile. The organic was dried off, 0.1% TFA added to 15 μl, and 5 μl injected onto LC–MS. Peptides were separated on an Ultimate nano HPLC instrument (ThermoFisher) and analyzed on either an Orbitrap Lumos or a Q Exactive Plus instrument (ThermoFisher).

Three sets of replicate samples were used for the BioID-RAB18 experiment with HeLa cells (Fig. 1 and Table S1). Two different WT clones and two different null genotypes of each of the RAB3GAP1-, RAB3GAP2-, and TRAPPC9-null cells were used (Fig. S1). Three sets of replicate samples were used in the BioID2(Gly40Ser)-SEC22A experiment (Fig. 3*D* and Table S5), though preparation of one “RAB3GAP1-null” replicate failed. In each experiment, each set of samples was prepared independently, and so these can be considered biological replicates.

### Analysis

After data-dependent acquisition of higher-energy collisional dissociation fragmentation spectra, data were analyzed using MaxQuant (version 2.2.0.0 for the BioID-RAB18 experiment and version 1.6.7.0 for the BioID2(Gly40Ser)-SEC22A experiment). For the BioID-RAB18 experiment, the UniProt Human 2022_05 database with 20,594 entries was searched. For the BioID2(Gly40Ser)-SEC22A experiment, the UniProt Human 2019_07 database with 20,667 entries was searched. Two missed/nonspecific cleavages were permitted. Fixed modification by carbamidomethylation of cysteine residues was considered. Variable modification by oxidation of methionine residues and N-terminal acetylation were considered. Mass error was set at 20 ppm for the first search tolerance and 4.5 ppm main search tolerance. Thresholds for accepting individual spectra were set at *p* < 0.05. Single-peptide identifications of proteins were used in analysis of the BioID-RAB18 experiment with single peptide identifications made “by modification site only” excluded. Percent of false discovery rate for these single-peptide identifications, and that for the combined dataset, was estimated at <5% using the decoy search method. Additional parameters and gradients used for separation are provided in Table S7. Annotated spectra for single-peptide identifications are provided in the “single_peptide_identifications” document in the supporting information section.

Quantification data were produced with MaxLFQ (27). For the BioID-RAB18 experiment, data were first processed to remove any protein identified in samples from control (untransfected, biotin treated) samples at high levels (>25% WT LFQ value) in any replicate from all replicates. Next, proteins identified in only one replicate sample set were removed. For each sample set, LFQ values were normalized according to the quantity of RAB18 detected in each sample. GEF-null:WT ratios for each protein were calculated for each replicate sample set, and then their means were calculated for the experiment (Table S1, columns “mean RAB3GAP ratio” and “mean TRAPII ratio”). The GEF-null:WT <0.5 criterion for selection of putative effectors (Fig. 1, *A* and *D* and Table 1) is an arbitrary cutoff rather than a measure of statistical validity. A similar approach was taken to analyze BioID2-SEC22A data (Table S5), except that “mean RAB3GAP ratio” and “mean RAB18 ratio” were calculated for each protein.

### Cell labeling

In order to distinguish cells of different genotypes within the same well/on the same coverslip, CellTrace-Violet and CellTrace-Far Red reagents (ThermoFisher) were used to label cells before they were seeded. Cells of different genotypes were first trypsinized and washed with PBS separately. They were then stained in suspension by incubation with either 1 μM CellTrace-Violet reagent or 200 nM CellTrace-Far Red reagent for 20 min at 37 °C. Remaining dye was removed by addition of a 10-fold excess of full media, incubation for a further 5 min, and then by centrifugation and resuspension of the resulting pellets in fresh media. Differently labeled cells were combined prior to seeding.

### Immunofluorescence microscopy

Cells were seeded in 96-well glass-bottom plates (PerkinElmer) coated with Matrigel (Corning) according to the manufacturer’s instructions and allowed to adhere for 48 h prior to fixation. In lipid-loading experiments, cells were treated with 200 μM oleic acid complexed to albumin (Merck) and 1 μg/ml BODIPY-558/568-C12 (ThermoFisher) for 15 h prior to fixation. Cells were fixed using a solution of 3% deionized glyoxal, 20% EtOH, 0.75% acetic acid, pH = 5 (88), for 20 min at room temperature. They were then washed with PBS containing 0.9 mM CaCl_2_ and 0.5 mM MgCl_2_ and blocked with a sterile-filtered buffer containing 1% milk, 2% donkey serum (Merck), 0.05% Triton X-100 (Merck), 0.9 mM CaCl_2_, and 0.5 mM MgCl_2_ in PBS (pH = 7.4) for at least 1 h prior to incubation with primary antibody. Primary antibodies were added in blocking buffer without Triton X-100, and plates were incubated overnight at 4 °C. Antibody dilutions are listed in Table S7. Following washing in PBS, cells were incubated with 1:2000 Alexa 488-conjugated secondary antibody (ThermoFisher) in blocking buffer at room temperature for 1 to 2 h. Following further washing in PBS, cells were imaged using an Operetta High Content Imaging System (PerkinElmer) equipped with Harmony software (PerkinElmer). In comparative fluorescence quantitation experiments, at least 18 frames—each containing >5 WT and >5 mutant cells—were analyzed per genotype. ImageJ software (National Institutes of Health) was used to produce regions of interest corresponding to each cell using thresholding tools and images from the 405 nm and 645 nm channels. Median 490 nm fluorescence intensity was measured for each cell, and mutant fluorescence intensity (as %WT) was calculated for each frame and combined for each genotype.

### Confocal microscopy—live cell imaging

HeLa or RPE1 cells were seeded on glass-bottom dishes (World Precision Instruments) coated with Matrigel (Corning) and allowed to adhere for 24 h prior to transfection. Transfections and cotransfections were carried out with 0.5 μg of each of the indicated constructs using Lipofectamine 2000 reagent in Opti-MEM serum-free medium for 4 h, according to the manufacturer’s instructions. Media were replaced, and cells were allowed to recover for at least 18 h prior to imaging. Imaging was carried out on a Nikon A1R confocal microscope equipped with the Nikon Perfect Focus System using a 60× oil immersion objective with a 1.4 numerical aperture. The pinhole was set to airy1. CellTrace-Violet reagent was excited using a 403.5 nm laser, and emitted light was collected at 425 to 475 nm. EGFP and mEmerald were excited using a 488 nm laser, and emitted light was collected at 500 to 550 nm. mCherry was excited using a 561.3 nm laser, and emitted light was collected at 570 to 620 nm. CellTrace-Far Red reagent was excited using a 638 nm laser, and emitted light was collected at 663 to 738 nm. Gain was adjusted to minimize collection of background fluorescence whilst selecting cells expressing relatively low levels of recombinant protein for imaging. Numbers of cells imaged are indicated in the figure legends.

### Immunoprecipitation

HeLa cells were seeded onto 10 cm dishes or 6-well plates and allowed to adhere for 24 h prior to transfection. Transfections and cotransfections were carried out with 0.5 μg of each of the indicated constructs using Lipofectamine 2000 reagent in Opti-MEM serum-free medium for 4 h, according to the manufacturer’s instructions. About 24 h post-transfection, cells were trypsinised, washed with PBS, then lysed in a buffer containing 150 mM NaCl, 0.5% Triton X-100 and EDTA-free protease-inhibitor solution, 10 mM Tris, pH = 7.4. Lysates were clarified by centrifugation, input samples taken, and the remaining supernatants then added to 4 μg rabbit anti-HA antibody (Merck). After 30 min incubation at 4 °C on a rotator, 100 μl washed protein G-coupled Dynabeads (ThermoFisher) were added and samples were incubated for a further 1 h. The Dynabeads were washed three times with buffer containing 150 mM NaCl, 0.1% Triton X-100, 10 mM Tris, pH = 7.4, then combined with a reducing loading buffer and subjected to SDS-PAGE.

### Generation of stable CHO cell lines

A PCR product encoding mouse RAB18 was subcloned into an intermediate TOPO vector using a TOPO PCR Cloning Kit (ThermoFisher) according to the manufacturer’s instructions. The RAB18 fragment was then excised and subcloned into the pCMV vector. PCR-based site-directed mutagenesis using a GeneArt kit (ThermoFisher) was then used to generate pCMV-RAB18(Gln67Leu) and pCMV-RAB18(Ser22Asn) constructs. CHO cells were transfected using Lipofectamine 2000 reagent (ThermoFisher), and cells stably expressing each construct were selected for with blasticidin. Under continued selection, clonal cell lines were grown from single cells, and then RAB18 protein expression was assessed. Cell lines comparably expressing RAB18 constructs at levels 2.5 to 5 times higher than those WT cells were used in subsequent experiments.

### Generation of stable T-Rex-293 cell lines

PCR products encoding mouse RAB18, RAB18(Gln67Leu), and RAB18(Ser22Asn) were subcloned into NotI-linearized pcDNA5 FRT/TO FLAG-BirA(Arg118Gly) or pcDNA5 FRT/TO vectors using the In-Fusion HD EcoDry Cloning Plus kit (Takara Bio) according to the manufacturer’s instructions. Details of PCR templates, primers, and target vectors are listed in Table S7. About 1.5 μg of each recombinant vector together with 13.5 μg of pOG44 plasmid (ThermoFisher) were used in cotransfections of T-REx-293 cells, in 10 cm dishes, with TransIT-LT1 Transfection Reagent (Mirus Bio). About 16 h following transfection, media were replaced and cells were allowed to recover for 24 h. Each dish was then split to four times 10 cm dishes in selection media containing 10 μg/ml blasticidin and 50 μg/ml hygromycin B. Resistant clones were pooled and passaged once prior to use.

### Lipid-loading experiments

For LD number and diameter measurements, IHH cells were seeded onto glass coverslips. siRNA transfections were carried out using FuGene reagent (Promega) according to the manufacturer’s instructions. siRNAs targeting ZW10 and NBAS were obtained from IDT; siRNA targeting SEC22A was obtained from Horizon Discovery. About 48 h following transfection, cells were treated with 200 nM bovine serum albumin–conjugated oleate for 24 h. Coverslips were washed, fixed with 3% paraformaldehyde, and stained with 1 μg/ml BODIPY and 300 nM 4′,6-diamidino-2-phenylindole. Fluorescence images were captured on a Zeiss LSM 780 confocal microscope equipped with a 100× objective. Images were analyzed using ImageJ software. Data are representative of three independent experiments.

For cholesterol storage and efflux experiments with [^14^C]-oleate, CHO cell lines (described previously) were seeded onto 12-well plates and then grown to 60 to 75% confluence in Alpha media supplemented with 10% LPDS. Cells were grown in the presence of 10% LPDS for at least 24 h prior to the addition of oleate.About 1 μCi/ml [^14^C]-oleate (PerkinElmer) was added in the presence of 10% LPDS or 10% FBS for 24 h. Cells were then washed and incubated with 50 μg/ml high-density lipoprotein for 0, 4, or 8 h. Cellular lipids were extracted with hexane. Lipids were then dried down and separated by TLC. Hexane:diethyl ether:acetic acid (80:20:2) and heptane:diethyl ether:methanol:acetic acid; 80:30:3:1.5 solvent systems were used. TLC plates were obtained from Analtech. Bands corresponding to CE were scraped from the TLC plate, and radioactivity was determined by scintillation counting in a Beckman Coulter LS6500 Scintillation Counter using BetaMax ES Liquid Scintillation Cocktail (ThermoFisher). Three independent experiments were carried out, each with four replicates of each condition. Data from a representative experiment are shown.

For cholesterol efflux experiments with [^3^H]-cholesterol, CHO cells were seeded onto 12-well plates and then grown to 60% confluence in Alpha media supplemented with 10% FBS. About 5 μCi/ml [^3^H]-cholesterol (PerkinElmer) was added in the presence of 10% FBS. After 3× PBS washes, cells were incubated with serum-free media containing 25 μg/ml of human apolipoprotein A-I for 5 h. Apolipoprotein A-I was a kind gift of Dr Paul Weers (California State University). Radioactivity in aliquots of media was determined by scintillation counting in a Beckman Coulter LS6500 Scintillation Counter using LSC Cocktail (PerkinElmer). Cell lysates were produced by addition of 0.1 N NaOH for 1 h, and their radioactivity was determined as aforementioned. Cholesterol efflux was calculated as an average (±SD) of the percent cholesterol efflux (as a ratio of the media cpm/[media + cellular cpm] × 100%).

For the cholesterol biosynthesis experiments, HeLa cells or HEK293 cells were seeded onto 12-well plates and then grown to 80% confluence in DMEM supplemented with 10% LPDS for 24 h. Following incubation with 5 μCi/well [^3^H]-mevalonate or 10 μCi/well [^3^H]-acetate for 24 h, TLC was used to separate free cholesterol, and radioactivity was quantified by scintillation counting as aforementioned.

### Sterol analysis (HeLa cells)

HeLa cells were grown to 80% confluence in T75 flasks, washed twice in PBS, and then grown for a further 48 h in DMEM supplemented with 10% LPDS. They were then trypsinised and washed twice in PBS before pellets were transferred to microcentrifuge tubes and snap frozen. Pellets were resuspended in 200 μl deionized water, sonicated for 20 s using an ultrasonic processor (Sonics & Materials, Inc), and then placed on ice. About 750 μl of isopropanol containing 4 μmol/l 5α-cholestane as an internal standard (ISTD) was added to each sample, and then each was sonicated for a further 10 s. Lysates were transferred to 7 ml glass vials and mixed with 250 μl tetramethylammonium hydroxide for alkaline saponification at 80 °C for 15 min and then cooled down for 10 min at room temperature. Sterols were extracted by addition of 500 μl tetrachloroethylene/methyl butyrate (1:3) and 2 ml deionized water, then thorough mixing. Samples were centrifuged for 10 min at 3000 rpm, and the organic phase containing the sterols was transferred to 300 μl GC vials. Extracts were dried under a stream of nitrogen, then sterols were silylated with 50 μl Tri-Sil HTP (HDMS:TMCS:pyridine) reagent (ThermoFisher) at 60 °C for 1 h.

Chromatography separation was performed on an Agilent GC–MS system (6890A GC and 5973 MS) (Agilent Technologies, Inc) with an HP-1MS capillary column (30 m length × 250 μm diameter × 0.25 μm film thickness). The GC temperature gradient was as follows: initial temperature of 120 °C increased to 200 °C at a rate of 20 °C/min and then increased to 300 °C at a rate of 2 °C/min with a 15 min solvent delay. Injection was at 250 °C in splitless mode with ultrapurified helium as the carrier gas, and the transfer line was 280 °C. The mass spectra were acquired by electron impact at 70 eV using SIM as follows: lathosterol-TMS, cholesterol-TMS, and cholest8(9)-enol-TMS: *m/z* 458; 5α-cholestane and desmosterol-TMS: *m/z* 372; lanosterol-TMS: *m/z* 393; and 7-dehydrocholesterol-TMS: *m/z* 325. The data were analyzed using MassHunter Workstation Quantitative Analysis Software (Agilent Technologies, Inc) and OriginPro 2017 (OriginLab Corp).

### GC–flame ionization detector and GC–MS–SIM sterol and stanol analysis (fibroblasts)

Gas chromatographic separation and detection of cholesterol and 5α-cholestane (ISTD) was performed on a DB-XLB 30 m × 0.25 mm i.d. × 0.25 μm film thickness (J&W Scientific Alltech) in an Hewlett–Packard (HP) 6890 Series GC-system (Agilent Technologies), equipped with a flame ionization detector (FID).

Noncholesterol sterols such as the cholesterol precursors lanosterol, 24.25-dihydrolanosterol, desmosterol, lathosterol, and the cholesterol metabolite 5α-cholestanol together with epicoprostanol (ISTD) were separated on another DB-XLB column (30 m × 0.25 mm i.d. × 0.25 μm film thickness; J&W Scientific Alltech) in an HP 6890N Network GC system (Agilent Technologies) connected with a direct capillary inlet system to a quadrupole mass selective detector HP5975B inert MSD (Agilent Technologies). Both GC systems were equipped with HP 7687 series autosamplers and HP 7683 series injectors (Agilent Technologies).

To determine the concentrations of cholesterol, noncholesterol precursor sterols and 5a-cholestanol, 50 μg 5α-cholestane (Serva) (50 μl from a stock solution of 5α-cholestane in cyclohexane [Merck KGaA]; 1 mg/ml), and 1 μg epicoprostanol (Sigma) (10 μl from a stock solution epicoprostanol in cyclohexane; 100 μg/ml) were added as ISTDs to 100 μl of a chloroform/methanol cell extract (5 ml chloroform/methanol, [2:1, v/v] per 10 mg dried cells). The cell pellet was dried for 12 h at room temperature in a Savant DNA120, Speed Vac Concentrator system (Thermo Fisher Scientific).

After saponification with 2 ml 1 M 95% ethanolic sodium hydroxide solution (Merck KGaA) at 60 °C for 1 h, the free sterols were extracted three times with 3 ml cyclohexane each from dried tissues (speedvac). The organic solvent was evaporated by a gentle stream of nitrogen at 60 °C on a heating block. The residue was dissolved in 80 μl *n*-decane (Merck KGaA). An aliquot of 40 μl was incubated (1 h at 70 °C on a heating block) by addition of 20 μl of the trimethylsilylating (TMSi) reagent (chlortrimethylsilane [Merck KGaA]/1.1.1.3.3.3-hexamethyldisilasane [Sigma–Aldrich, Co]/pyridine [Merck KGaA], 9:3:1) in a GC vial for GC–MS–SIM noncholesterol analysis. Another aliquot of 40 μl was incubated by addition of 40 μl of the TMSi reagent and dilution with 300 μl *n*-decane in a GC vial for GC–FID cholesterol analysis.

An aliquot of 2 μl was injected by automated injection in a splitless mode using helium (1 ml/min) as carrier gas for GC–MS–SIM and hydrogen (1 ml/min) for GC–FID analysis at an injection temperature of 280 °C. The temperature program for GC was as follows: 150 °C for 3 min, followed by 20 °C/min up to 290 °C keeping for 34 min. For MSD, electron impact ionization was applied with 70 eV. SIM was performed by cycling the quadrupole mass filter between different *m/z* at a rate of 3.7 cycles/s. Noncholesterol sterols were monitored as their TMSi-, the oxysterols as their di-TMSi derivatives using the following masses: epicoprostanol *m/z* 370, lathosterol at *m/z* 458, desmosterol at *m/z* 441, lanosterol at *m/z* 393, 24.25-dihydrolanosterol at *m/z* 395 (M^+^-90-15, M^+^-OTMSi-CH_3_), and 5α-cholestanol at *m/z* 306. Peak integration was performed manually. Cholesterol was directly quantified by multiplying the ratios of the area under the curve of cholesterol to 5α-cholestane by 50 μg (ISTD amount). Noncholesterol sterols and 5α-cholestanol were quantified from the ratios of the areas under the curve of the respective noncholesterol sterols and 5α-cholestanol after SIM analyses against epicoprostanol using standard curves for the listed sterols/stanol. Identity of all sterols and 5α-cholestanol was proven by comparison with the full-scan mass spectra of authentic compounds. Additional qualifiers (characteristic fragment ions) were used for structural identification (*m/z* values not shown).

### Lentivirus generation and transduction

For generation of lentiviral particles, oligonucleotide pairs (10 μM each) incorporating gRNA sequences (Table S7) were phosphorylated and annealed using T4 PNK enzyme (New England Biolabs) incubating for 30 min at 37 °C, then 5 min at 95 °C, followed by cooling to room temperature. Annealed oligonucleotides were diluted and then ligated into lentiCRISPRv2 transfer vector (Addgene) using Golden Gate cloning with the ESP3I restriction enzyme (ThermoFisher Scientific) and T7 ligase (New England Biolabs) as previously described (89). Recombinant transfer vectors were verified and used together with pMD2.G and psPAX2 vectors (Addgene) encoding envelope and packaging constituents, and polyethylenimine (Sigma–Aldrich), to transfect HEK293FT cells in 10 cm plates. Lentiviral particles were collected from supernatants 48 h post-transfection.

For transduction of the HEK293 cell lines with the lentiviral particles, cells were first treated with 10 μg/ml polybrene (Sigma–Aldrich), then particles were added at 0.3 to 0.5 multiplicity of infection. Following overnight incubation, the medium was replaced and then following overnight recovery, cells were treated with 2 μg/ml puromycin (Invitrogen). Cholesterol biosynthesis experiments were carried out as described previously following at least 7 days of puromycin selection.

### Western blotting

Cell lysates were made with a buffer containing 150 mM NaCl, 0.5% Triton X-100, and EDTA-free protease-inhibitor solution, 50 mM Tris, pH = 7.4. Cell lysates and input samples from BioID and immunoprecipitation experiments were combined 1:1 with a 2× reducing loading buffer; a reducing loading buffer containing 10 mM EDTA was added directly to Dynabead samples. SDS-PAGE and Western blotting were carried out according to standard methods. Transfer onto polyvinylidene difluoride membranes was carried out using either the iBlot system (Invitrogen) or the Trans-Blot Turbo system (Bio-Rad) according to the manufacturer’s instructions. Primary antibody dilutions are given in Table S7. Blots were probed with appropriate horseradish peroxidase–conjugated secondary antibodies and detected using Pierce ECL Plus Western Blotting Substrate (ThermoFisher). Luminescence was detected using either Hyperfilm ECL film (ThermoFisher) and an automatic film processor or directly using a Gel Doc XR+ device (Bio-Rad).

## Data availability

The MS proteomics data have been deposited to the ProteomeXchange Consortium *via* the PRIDE (90) partner repository with the dataset identifiers PXD016631, PXD016336, PXD016326, PXD016233, and PXD016404.

## Supporting information

This article contains supporting information (9, 13, 17, 18, 27, 39, 40, 41, 44, 45, 47, 48, 49, 53, 54, 55, 56, 57, 58, 59, 91, 92, 93, 94, 95, 96).

## Conflict of interest

The authors declare that they have no conflicts of interest with the contents of this article.

## Supporting information

Supporting information

Single_peptide_identification
