# Supplementary material for: Comparative proximity biotinylation implicates the small GTPase RAB18 in sterol mobilization and biosynthesis
Source: J Biol Chem. 2023 Sep 28;299(11):105295. doi: 10.1016/j.jbc.2023.105295 (PMC10641524; doi:10.1016/j.jbc.2023.105295)
Supplement: Supporting information [file mmc1.pdf]

SUPPORTING INFORMATION

TITLE

Comparative proximity biotinylation implicates the small GTPase RAB18 in sterol mobilization and biosynthesis

RUNNING TITLE

GEF-dependent RAB18 interactions

AUTHORS

Robert S. Kiss*[1], Jarred Chicoine[2], Youssef Khalil[3], Robert Sladek[2], He Chen[1], Alessandro Pisaturo[1], Cyril Martin[1], Jessica D. Dale[4], Tegan A. Brudenell[4], Archith Kamath[5,6], Jeffrey Kyei-Boahen[7], Anouar Hafiane[7], Girija Daliah[8], Célia Alecki[9], Tayah S. Hopes[10], Martin Heier[11], Irene A. Aligianis[12], Jean-Jacques Lebrun[8], Julie Aspden[10], Emanuele Paci[13], Anja Kerksiek[14], Dieter Lütjohann[14], Peter Clayton[3], Jimi C. Wills[15,16], Alex von Kriegsheim[15], Tommy Nilsson[17], Eamonn Sheridan[4], Mark T. Handley*[4,10]

AFFILIATIONS

[1]Cardiovascular Health Across the Lifespan (CHAL) program, Research Institute of the McGill University Health Centre, Montreal, Quebec, Canada

[2]Metabolic Disorders and Complications (MEDIC) program, Research Institute of the McGill University Health Centre, Montreal, Quebec, Canada

[3]Genetics and Genomic Medicine, Great Ormond Street Institute of Child Health, University College London, London, United Kingdom

[4]Leeds Institute of Medical Research, St James's University Hospital, Leeds, United Kingdom

[5]MRC Human Genetics Unit, Institute of Genetics and Cancer, University of Edinburgh, Edinburgh, United Kingdom

[6]Division of Medical Sciences, University of Oxford, Oxford, United Kingdom

[7]Department of Medicine, McGill University Health Centre, CHAL Research Program, Montreal, Canada

[8]Department of Medicine, McGill University Health Centre, Cancer Research Program, Montreal, Canada

[9]Department of Biochemistry, McGill University, Montreal, Quebec, Canada

[10]Faculty of Biological Sciences, University of Leeds, Leeds, United Kingdom

[11]Department of Clinical Neuroscience for Children, Oslo University Hospital, Oslo, Norway

[12]Medical and Developmental Genetics, Medical Research Council Human Genetics Unit, Edinburgh, United Kingdom

[13]Astbury Centre for Structural Molecular Biology, University of Leeds, Leeds, United Kingdom

[14]Institute of Clinical Chemistry and Clinical Pharmacology, University Hospital Bonn, Bonn, Germany

[15]Cancer Research United Kingdom Edinburgh Centre, Institute of Genetics and Cancer, University of Edinburgh, Edinburgh, United Kingdom

[16]Firefinch Software Ltd, Edinburgh, United Kingdom

[17]Cancer Research Program (CRP), Research Institute of the McGill University Health Centre, Montreal, Quebec, Canada

CORRESPONDING AUTHORS

Robert S. Kiss: robert.kiss@mcgill.ca

Mark T. Handley: mark.handley@protonmail.com

*authors contributed equally

A

ID#	Clone name	Cell type	Targeted gene	Reference sequence	Targeted exon	gRNA sequences	cDNA alleles	protein alleles	Further validation
1	RAB18_x7c14	HeLa	*RAB18*	NM_021252.5	7	ggcacattgtacaccatcac<br>gcattcagacccctggactgt	c.457_512del<br>c.455_523delins49<br>c.446-12_532delins2	p.Lys153Aspfs*7<br>p.Ala152Glufs*20	Fig. S1B
2	RAB18_x7c21	HeLa	*RAB18*	NM_021252.5	7	ggcacattgtacaccatcac<br>gcattcagacccctggactgt	c.456_523del<br>c.455_527del<br>c.472_490del	p.Lys153Valfs*3<br>p.Ala152Glyfs*44<br>p.Val158Asnfs*56	Fig. S1B
3	RAB18_x7c31	RPE1	*RAB18*	NM_021252.5	7	ggcacattgtacaccatcac<br>gcattcagacccctggactgt	c.477_485delins5<br>c.477_521delins28	p.Cys160Glnfs*59<br>p.Glu164Lysfs*9	Fig. S1B
4	RAB18_x7c32	RPE1	*RAB18*	NM_021252.5	7	ggcacattgtacaccatcac<br>gcattcagacccctggactgt	c.477_485delins5<br>c.477_521delins28	p.Cys160Glnfs*59<br>p.Glu164Lysfs*9	Fig. S1B
5	RAB3GAP1_x15c3	HeLa	*RAB3GAP1*	NM_012233.3	15	gccactcctttcaaccctcca<br>gtcttgaaatgcgtttccgat	c.1417_1423del<br>c.1416_1432del	p.Gly473Lysfs*22<br>p.Gly473Thrfs*8	Fig. S1C
6	RAB3GAP1x15c5	HeLa	*RAB3GAP1*	NM_012233.3	15	gccactcctttcaaccctcca<br>gtcttgaaatgcgtttccgat	c.1471_1472ins148<br>c.1453_1499+16del	p.Arg491Thrfs*8	Fig. S1C
7	RAB3GAP1_x15c23	RPE1	*RAB3GAP1*	NM_012233.3	15	gccactcctttcaaccctcca<br>gtcttgaaatgcgtttccgat	c.1429_1448del<br>c.1444_1445ins79	p.Val477Ilefs*3<br>p.Gln482Leu*6	Fig. S1C
8	RAB3GAP1_x15c24	RPE1	*RAB3GAP1*	NM_012233.3	15	gccactcctttcaaccctcca<br>gtcttgaaatgcgtttccgat	c.1419_1432delins28<br>c.1452_1453ins26	p.Leu474Valfs*28<br>p.Val485Profs*21	
9	RAB3GAP2_x14c2	HeLa	*RAB3GAP2*	NM_012414.4	14	gaggaattgagctactcgact<br>gtgatctatgcgccaagaag	c.1385_1424del<br>c.1382_1397delins3<br>c.1381_1382insC	p.Ala462Glufs*11<br>p.Val461Glufs*10<br>p.Val461Alafs*24	Fig. S1D
10	RAB3GAP2_x14c26	RPE1	*RAB3GAP2*	NM_012414.4	14	gaggaattgagctactcgact<br>gtgatctatgcgccaagaag	c.1409_1410ins75<br>c.1408_1409ins16	p.Arg471*<br>p.Pro470Ilefs*3	Fig. S1D
11	RAB3GAP2_x20c12	HeLa	*RAB3GAP2*	NM_012414.4	20	gtatatttctctagtaatgcc<br>gcgattttctgatgataaaga	c.2056_2116del<br>c.2109_2110ins133<br>c.2058_2059ins6; c.2087_2097del	p.Leu686Valfs*4<br>p.Lys704Tyrfs*1<br>p.Leu686_Glu687insLeuLeu; p.Thr696Ilefs*2	Fig. S1D
12	RAB3GAP2_x20c32	RPE1	*RAB3GAP2*	NM_012414.4	20	gtatatttctctagtaatgcc<br>gcgattttctgatgataaaga	c.2100_2101ins88<br>c.2100_2101ins49	p.Ser701Lysfs*7<br>p.Ser701Ilefs*46	Fig. S1D
13	SPG20_x3c19	HeLa	*SPART*	NM_015087.5	3	gaatgtctgaccttcggctcc<br>gaagagctctctatcatcctc	c.962_977del<br>c.957_1006delins48	p.Glu321Glyfs*1<br>p.Asp319Glufs*27	Fig. 2C
14	TBC1D20_5H2	HeLa	*TBC1D20*	NM_144628.4	5	gtgcttggtgttgtccattgt<br>gtctgatgcccatcattgacc	c.558_586del<br>c.551_594del	p.Lys186Asnfs*1<br>p.Asn184Serfs*6	Fig. S1E
15	TBC1D20_x5c31	RPE1	*TBC1D20*	NM_144628.4	5	gtgcttggtgttgtccattgt<br>gtctgatgcccatcattgacc	c.547_583dup<br>c.567_568ins39	p.Ile195Lysfs*13<br>p.Asn190_Glu197insProThrGluThrSerCysSer*	
16	TBC1D20_x5c33	RPE1	*TBC1D20*	NM_144628.4	5	gtgcttggtgttgtccattgt<br>gtctgatgcccatcattgacc	c.577_578ins85<br>c.583_584ins83	p.Met193Thrfs*6<br>p.Ile195Thrfs*5	Fig. S1E
17	TBC1D20_7H5	HeLa	*TBC1D20*	NM_144628.4	7	gctgctgatcagtgtctcata<br>gcccatccgaacttgctcggg	c.890_929del<br>c.860_923del	p.Asp297Glyfs*49<br>p.Pro287Leufs*51	Fig. S1E
18	TRAPPC9_x13c1	HeLa	*TRAPPC9*	NM_001160372.4	13	ggagttcgagtctctccctg<br>gcgacgcagcatcgtaagcc	c.1872_1897del<br>c.1855-46_1865del	p.Val625Alafs*5	
19	TRAPPC9_x13c3	RPE1	*TRAPPC9*	NM_001160372.4	13	ggagttcgagtctctccctg<br>gcgacgcagcatcgtaagcc	c.1855-25_1880del<br>c.1855-13_1896del		
20	TRAPPC9_x14c6	HeLa	*TRAPPC9*	NM_001160372.4	14	gcgcgggaatgacttccactg<br>gtgcccaggtagtgtgcacg	c.2108_2114+4delins28<br>c.2058_2114+24del		

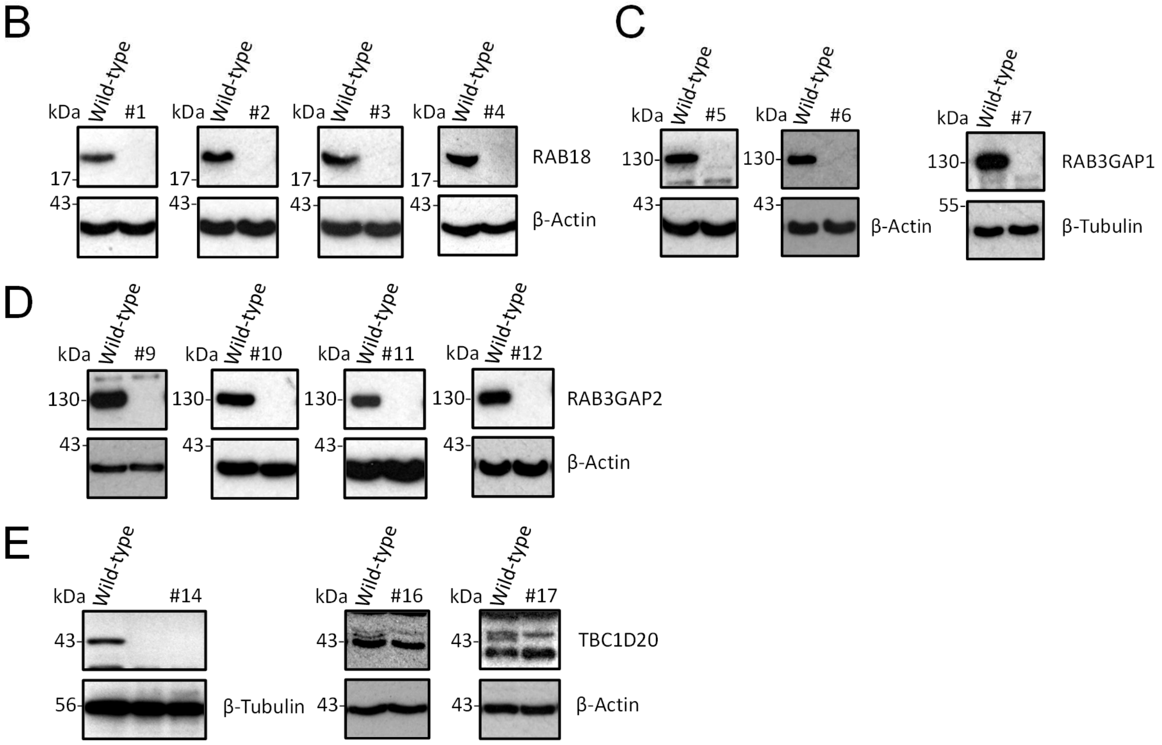

Figure S1. Genetically-validated gene-edited clonal cell lines used in this study. (A) Table to show clonal cell lines generated by transient expression of Cas9n together with the indicated guide RNAs. Mutant alleles of the targeted genes were sequenced following their cloning into ZeroBlunt TOPO vector. (B) Western

blotting for RAB18 shows that RAB18 is undetectable in RAB18-null cell lines. (C) Western blotting for RAB3GAP1 shows that RAB3GAP1 is undetectable in RAB3GAP1-null cell lines. (D) Western blotting for RAB3GAP2 shows that RAB3GAP2 is undetectable in RAB3GAP2-null cell lines. (E) Western blotting for TBC1D20 shows that TBC1D20 is undetectable in TBC1D20-null cell lines. Numbering in S1A corresponds to lane numbering in S1B-E. Loading controls are β-Actin or β-Tubulin. Note that different staining patterns for anti-TBC1D20 antibody in S1E are the result of antibody batch-variability.

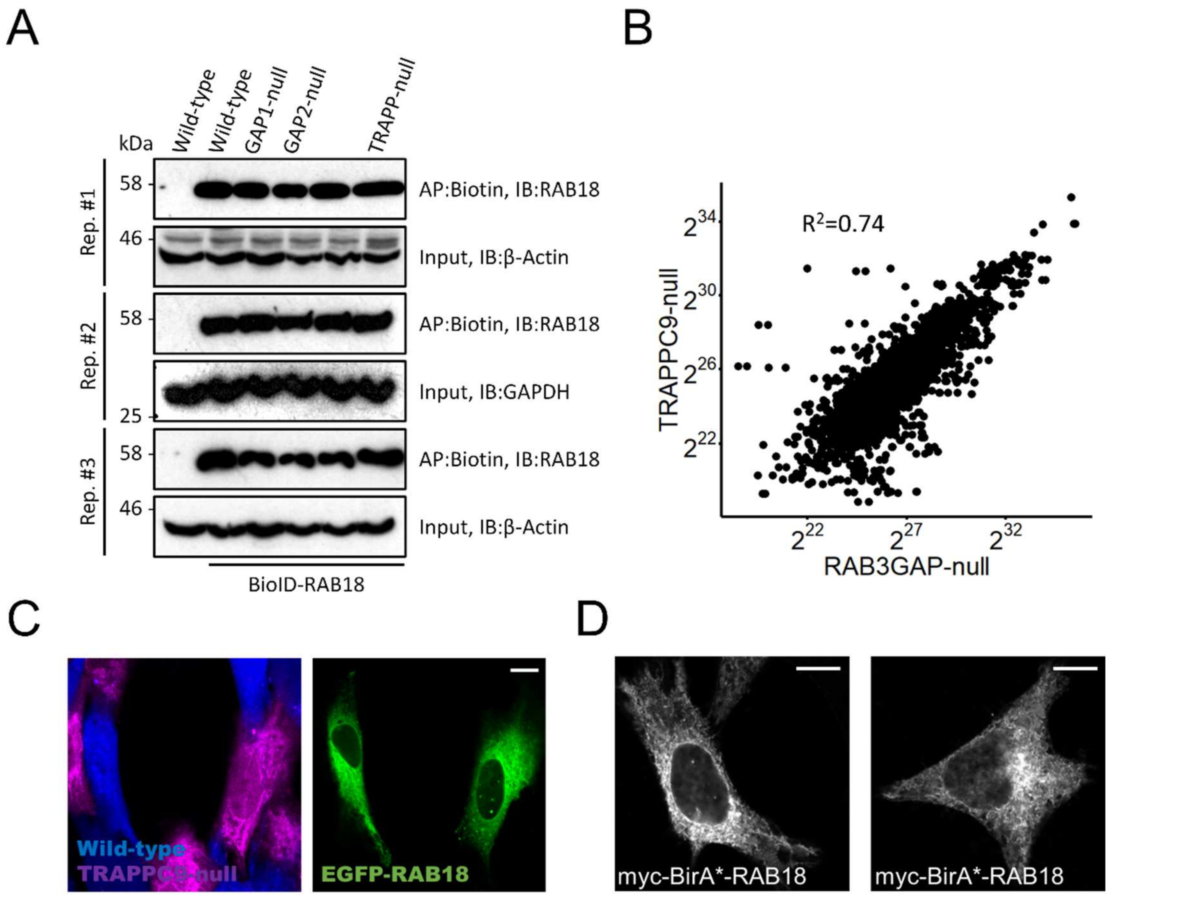

Figure S2. Controls for HeLa BioID experiments. (A) Western blotting to show comparable levels of BirA*-RAB18 in BioID samples from HeLa cells of wild-type and different mutant genotypes. Loading controls are β-Actin or GAPDH. (B) Plots to show correlations between non-zero LFQP intensities of individual proteins identified in samples purified from TRAPPC9-null cells and those purified from RAB3GAP1/2-null cells. (C) Confocal micrograph to show comparable localization of exogenous EGFP-RAB18 (Green) in wild-type and TRAPPC9-null HeLa cells. Cells were labelled with CellTrace-Violet and CellTrace-Far Red reagents respectively (magenta and blue channels). (D) Confocal micrographs showing the localization of BirA*-RAB18 in HeLa cells. Transfected HeLa cells were fixed with 3% deionized glyoxal, then permeabilized and stained with a mouse monoclonal anti-myc antibody and an Alexa-488-conjugated anti-mouse secondary antibody. Bars 10µm.

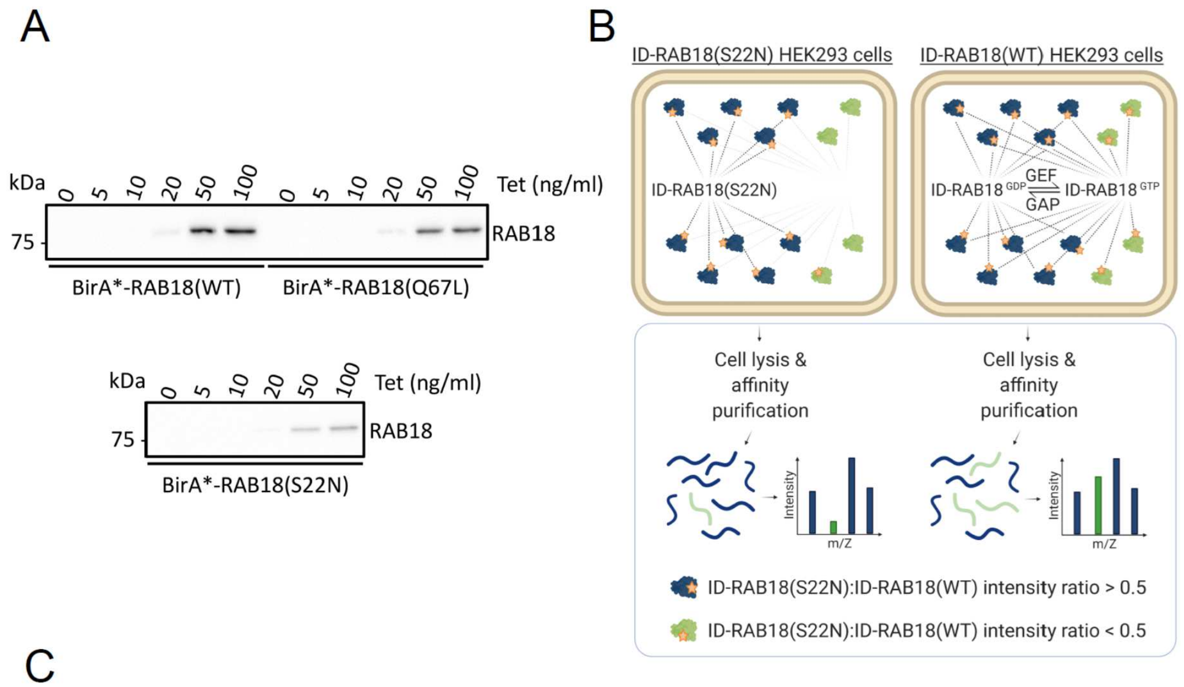

C

Protein	Ratio (see Fig. S3B)	Orthologue PPI (Gillingham et al., 2014)	Additional evidence	
NBAS	0.00	rod	Xu et al., 2018; Gillingham et al., 2019	RAB3GAP-dependent association in HeLa cells
SCFD2	0.00	Slh	Gillingham et al., 2019	
ZW10	0.00	mit(1)15	Xu et al., 2018; Gillingham et al., 2019	
CAMSAP1	0.04			
RINT1	0.41	CG8605	Xu et al., 2018	
BNIP1	0.65		Xu et al., 2018	
RAB3GAP2	1.58	rab3-GAP	Gerondopoulos et al., 2014	
RAB3GAP1	1.59	CG31935	Gerondopoulos et al., 2014	
ARPIN	0.00			TRAPPII-dependent association in HeLa cells
SEC23IP	0.00			
TBC1D5	0.00	CG8449	Gillingham et al., 2019	
ATP6AP2	0.29			
GORASP2	0.36			
GIGYF2	0.41			
EFHD2	0.50			
BET1	0.54			
RABL3	0.58			
CEP170	0.63			
ARFGAP1	0.65			
EPS15L1	0.65			
MKL2	0.77			
GOLGA5	0.79			
CLCC1	0.89			
ABCD3	0.93			
SEC22B	0.96			
AKAP1	1.47			
HDLBP	1.50			
DNML1	1.59			

Figure S3. Nucleotide-binding-dependent RAB18-associations in HEK293 cells. (A) Tetracycline-induced expression of BirA*-RAB18 constructs in stable HEK293 cell lines. PCR products encoding mouse RAB18, RAB18(Gln67Leu) and RAB18(Ser22Asn) were subcloned into a pcDNA5 FRT/TO FLAG-BirA(Arg118Gly) vector. Recombinant vectors were used together with pOG44 in cotrasfections of T-REx-293 cells. Stable cell lines were selected with Blasticidin and Hygromycin B.

Expression of recombinant RAB18 constructs in response to tetracycline was determined by Western blotting and densitometry. (B) Schematic to show experimental approach. Proximity biotinylation of nucleotide-binding-dependent RAB18 interactors is disrupted for the BirA*-RAB18(Ser22Asn) mutant. In contrast, BirA*-RAB18(WT) engages in both GDP-dependent and GTP-dependent interactions. Following affinity purification, nucleotide-binding-dependent interactions are determined by LFQ intensity ratios. (C) Table to show nucleotide-binding-dependent RAB18-associations with BirA*-RAB18(Ser22Asn):BirA*-RAB18(WT) association ratios <0.5. Ratios for each protein are calculated from spectral counts in samples purified from cells stably expressing BirA*-RAB18(Ser22Asn) divided by those purified from cells stably expressing from BirA*-RAB18. Orthologous proteins identified by Gillingham et al., 2014, and other studies providing supporting evidence for interactions are shown. Proteins are grouped according to their attributes in the HeLa cell dataset (Figure 1 and Table S1). Ratios were derived individually following normalization by total spectral counts per condition. The full dataset is provided in Table S2.

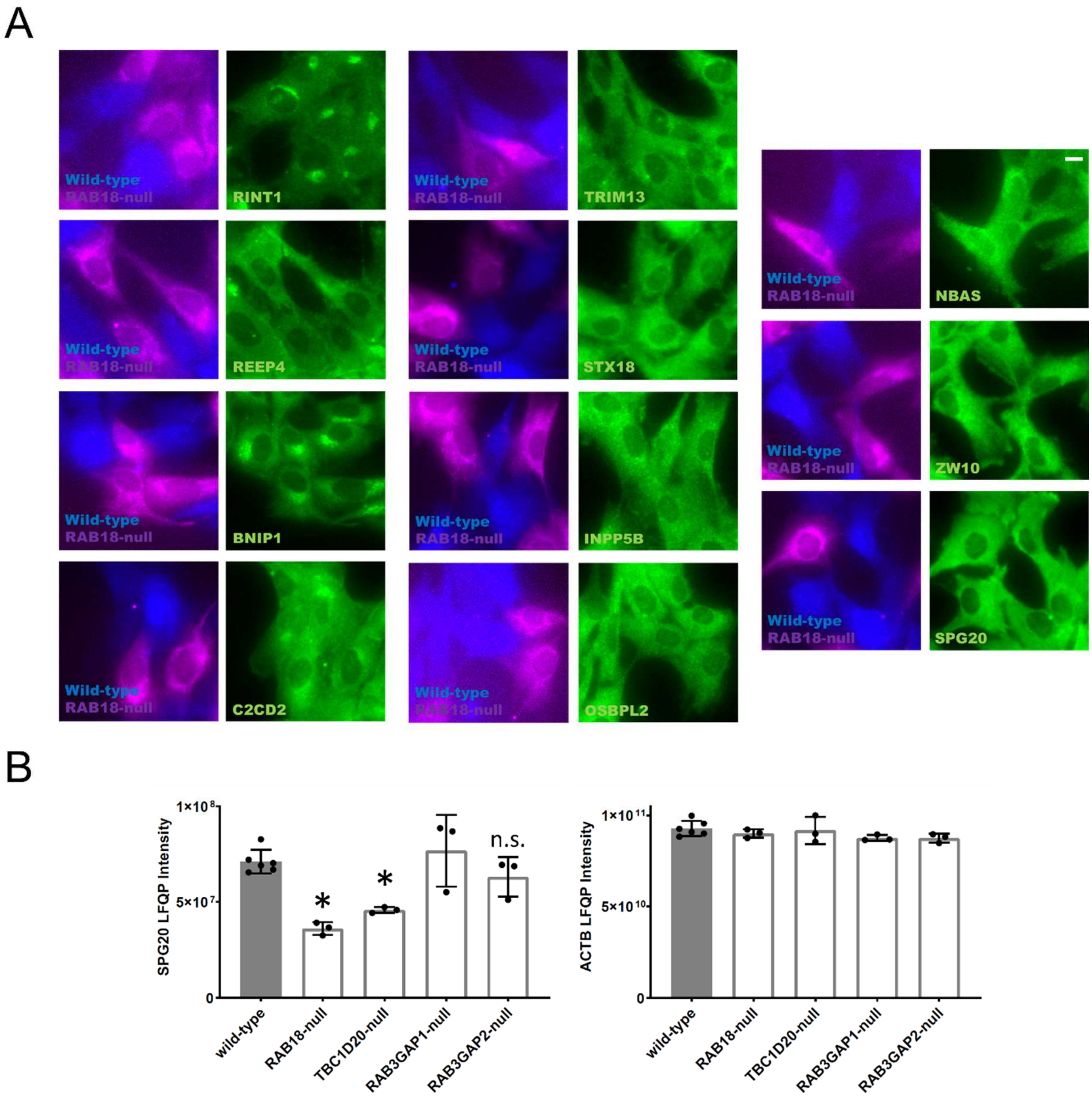

Figure S4. Comparative fluorescence microscopy of selected RAB18-associated proteins in wild-type and RAB18-null RPE1 cells and quantification of SPG20 levels in RPE1 cells of different genotypes. Cells of different genotypes were labelled with CellTrace-Violet and CellTrace-Far Red reagents corresponding to blue and magenta channels respectively. Cells were stained with antibodies against indicated proteins in green channel panels. (B) LFQ intensities for SPG20 (Q8N0X7) and β-Actin (P60709) in whole-cell lysates of RPE1 cells of the indicated genotypes. n=3; *$p<0.05$ following FDR correction. Full dataset provided in Table S3. Error bars represent s.e.m. Bar 10μm.

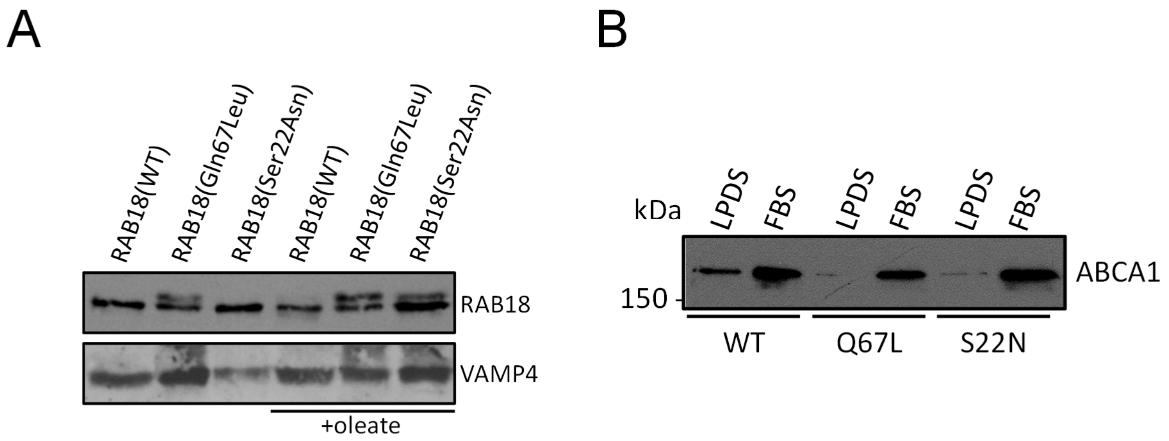

Figure S5. Characterization of stable CHO cell lines expressing RAB18 constructs. (A) Comparable expression of RAB18 constructs in stable CHO cell lines. Lysates were prepared from cells cultured in the absence and presence of oleate. Total protein in cell lysates was quantified by Bradford assay. Western blotting shows that levels of RAB18 and VAMP4 are comparable between cell lines. (B) Comparable levels of ABCA1 in in stable CHO cell lines. Lysates were prepared from cells grown in media supplemented with lipoprotein-deficient serum (LPDS) or FBS. Total protein in cell lysates was quantified by Bradford assay. Western blotting shows that levels of ABCA1 are comparable between cell lines under each condition.

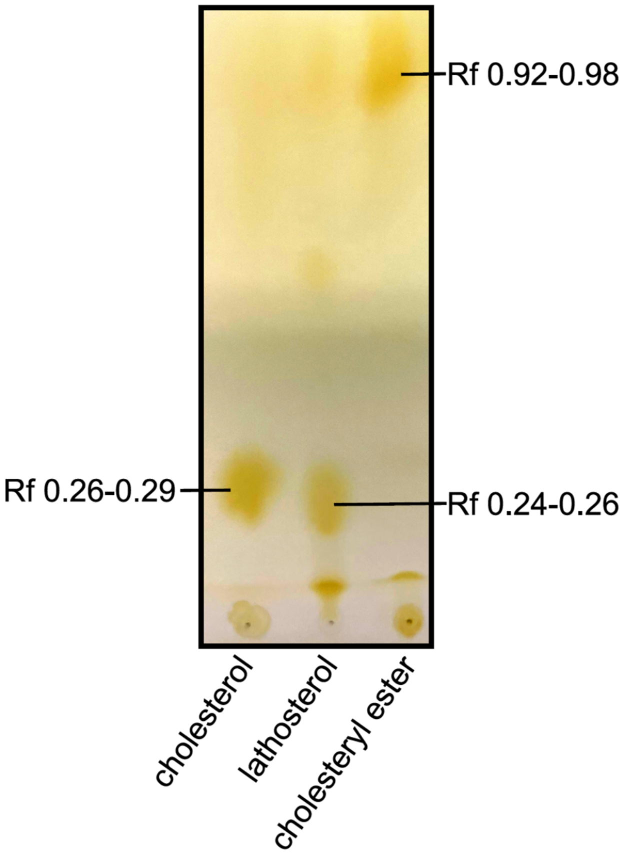

Figure S6. Thin layer chromatography of sterol standards. Migration of the indicated cold standards on an Analtech TLC plate under a heptane:diethyl ether:methanol:acetic acid; 80:30:3:1.5 solvent system is shown. The displayed Rf values were calculated from three replicate experiments.

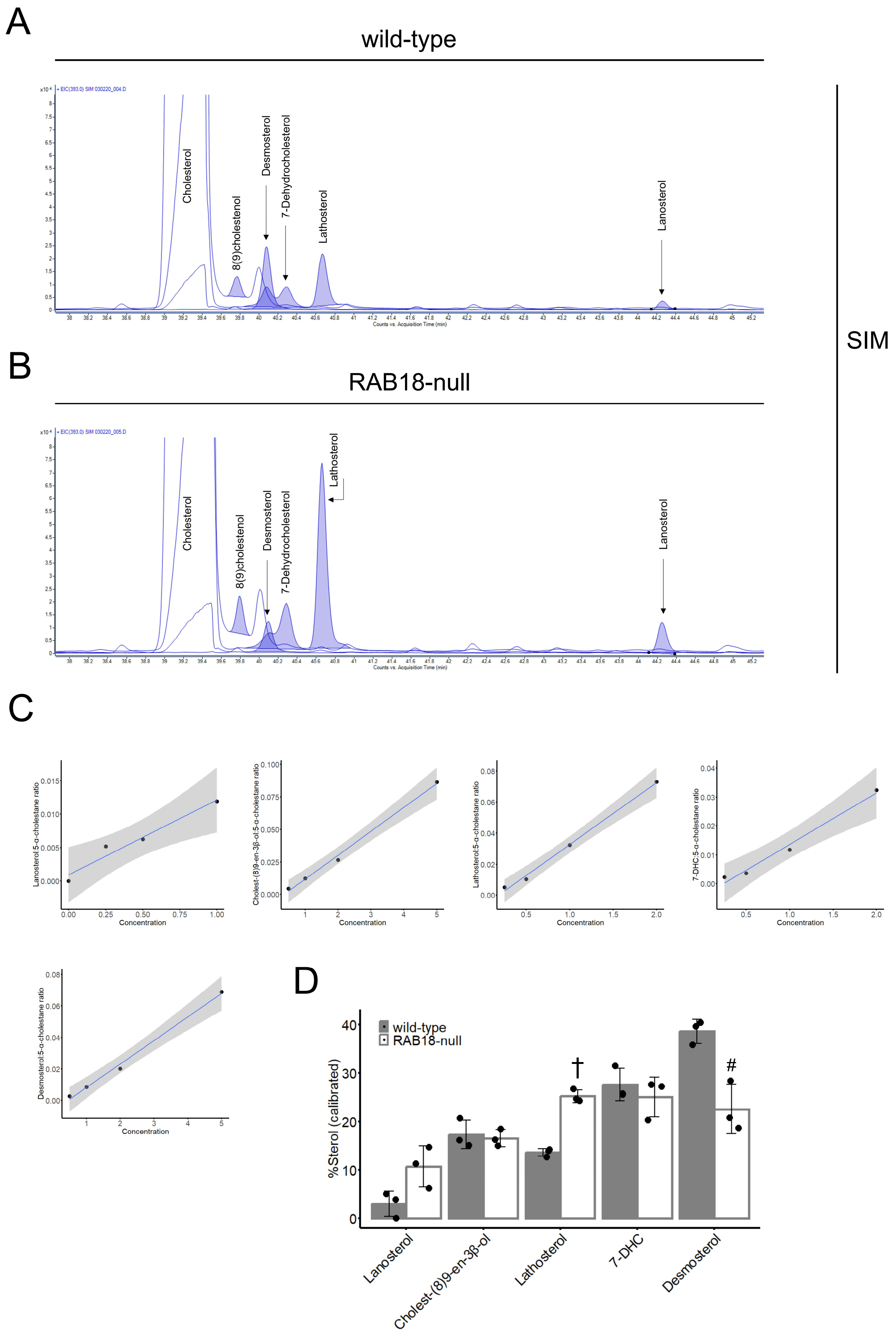

A
wild-type
Cholesterol
8(9)cholestenol
Desmosterol
7-Dehydrocholesterol
Lathosterol
Lanosterol
Counts vs. Acquisition Time (min)
SIM
B
RAB18-null
Cholesterol
8(9)cholestenol
Desmosterol
7-Dehydrocholesterol
Lathosterol
Lanosterol
Counts vs. Acquisition Time (min)
C
Lanosterol:5-α-cholestane ratio
Cholest-(8)9-en-3β-ol:5-α-cholestane ratio
Lathosterol:5-α-cholestane ratio
7-DHC:5-α-cholestane ratio
Desmosterol:5-α-cholestane ratio
Concentration
D
wild-type
RAB18-null
%Sterol (calibrated)
†
#
Lanosterol
Cholest-(8)9-en-3β-ol
Lathosterol
7-DHC
Desmosterol

Figure S7. Sterols profiling of wild-type and RAB18-null HeLa cells. (A-B) Representative chromatograms from profiling. Cells were grown in media supplemented with LPDS for 48 hours then harvested. Extracted sterols were analysed by GC-MS-SIM using Agilent 6890A GC and 5973 MS instruments. Sample processing, gradients and data analysis are described in Experimental Procedures. Wild-type and RAB18-null samples are indicated. (C) Sterols calibration plots. Known concentrations of sterol standards and an internal standard (5-α-cholestane) were analysed and sterol:standard ratios plotted against concentration to give linear equations for calculation of sterol levels from sterol:standard ratios in test samples. (D) Bar graph of calibrated sterols profile in wild-type and RAB18-null HeLa cells. Cells were grown in media supplemented with LPDS for 48 hours. Extracted sterols were analysed by GC-MS-SIM. '%Sterol (calibrated)' was calculated as a proportion of total quantified sterols, excluding cholesterol, . n=3; ±SD. $^{\#}p<0.05$, †$p<0.001$.

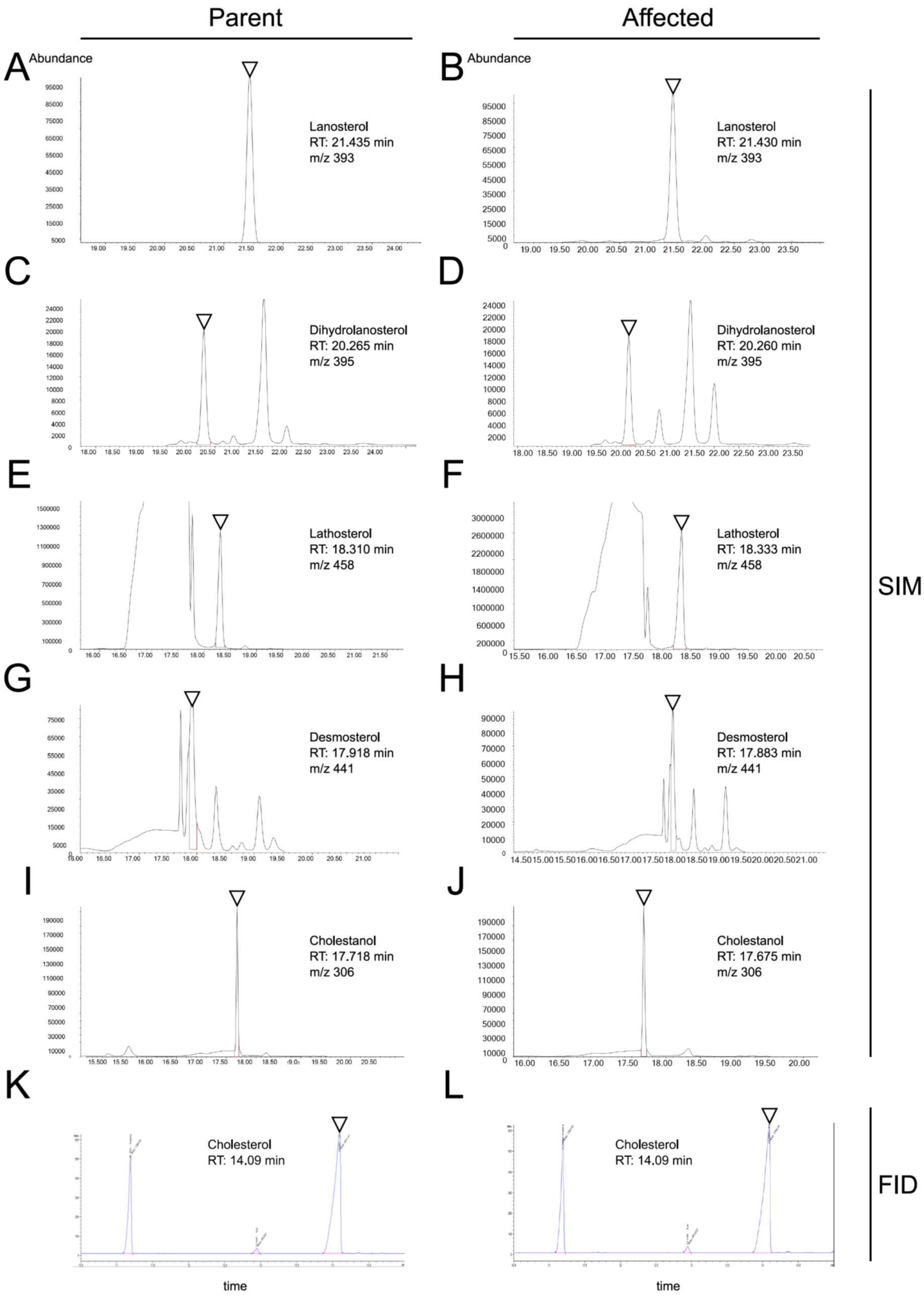
Parent
Affected
A
Abundance
Lanosterol
RT: 21.435 min
m/z 393
B
Abundance
Lanosterol
RT: 21.430 min
m/z 393
C
Dihydrolanosterol
RT: 20.265 min
m/z 395
D
Dihydrolanosterol
RT: 20.260 min
m/z 395
E
Lathosterol
RT: 18.310 min
m/z 458
F
Lathosterol
RT: 18.333 min
m/z 458
G
Desmosterol
RT: 17.918 min
m/z 441
H
Desmosterol
RT: 17.883 min
m/z 441
I
Cholestanol
RT: 17.718 min
m/z 306
J
Cholestanol
RT: 17.675 min
m/z 306
K
Cholesterol
RT: 14.09 min
time
L
Cholesterol
RT: 14.09 min
time
SIM
FID

Figure S8. Representative chromatograms from sterols profiling of human fibroblasts. Cells were grown in media supplemented with LPDS for 48 hours then harvested. Extracted sterols were analysed by GC-MS-SIM using Agilent HP6890N GC and HP5975B MS instruments. Cholesterol was quantified by GC-FID. Sample processing, gradients and data analysis are described in Experimental Procedures. Representative chromatograms are as follows (A, B) lanosterol, (C, D) dihydrolanosterol, (E, F) lathosterol, (G, H) desmosterol, (I, J) cholestanol, (K, L) cholesterol. Samples from parental fibroblasts (A, C, E, G, I, K) and RAB3GAP1-null fibroblasts from a Micro syndrome individual (B, D, F, H, J, L) are shown.

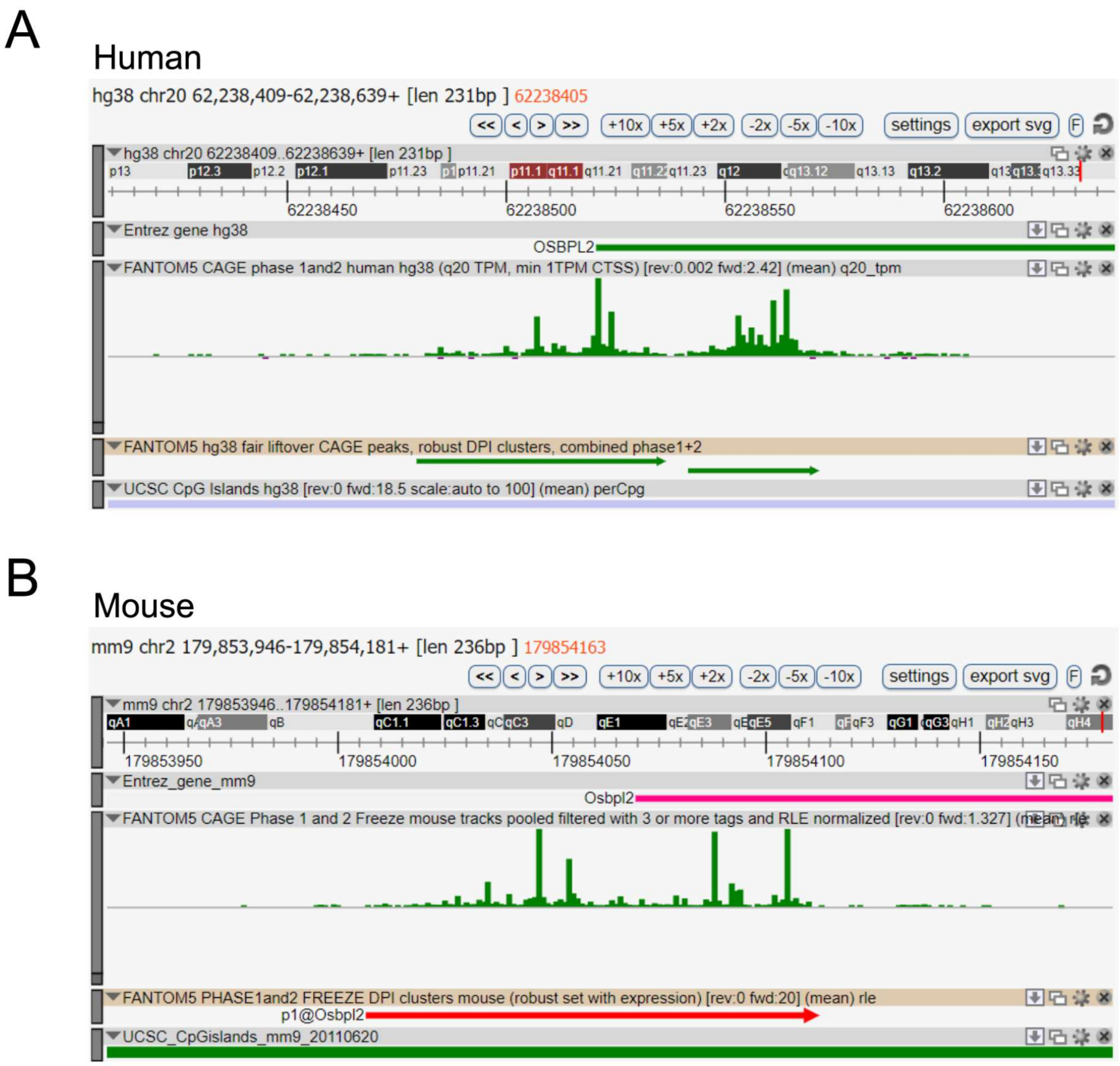

Figure S9. Transcription start sites for human *OSBPL2* and mouse *Osbpl2*. Probable transcription start sites are indicated by cap analysis of gene expression (CAGE) signal (1). Tracks for assembly, gene, CAGE signal, DPI (decomposition-based peak calls), and CpG islands are shown. Screenshots taken from the Zenbu genome browser v3.0, https://fantom.gsc.riken.jp/zenbu/, July 2023.

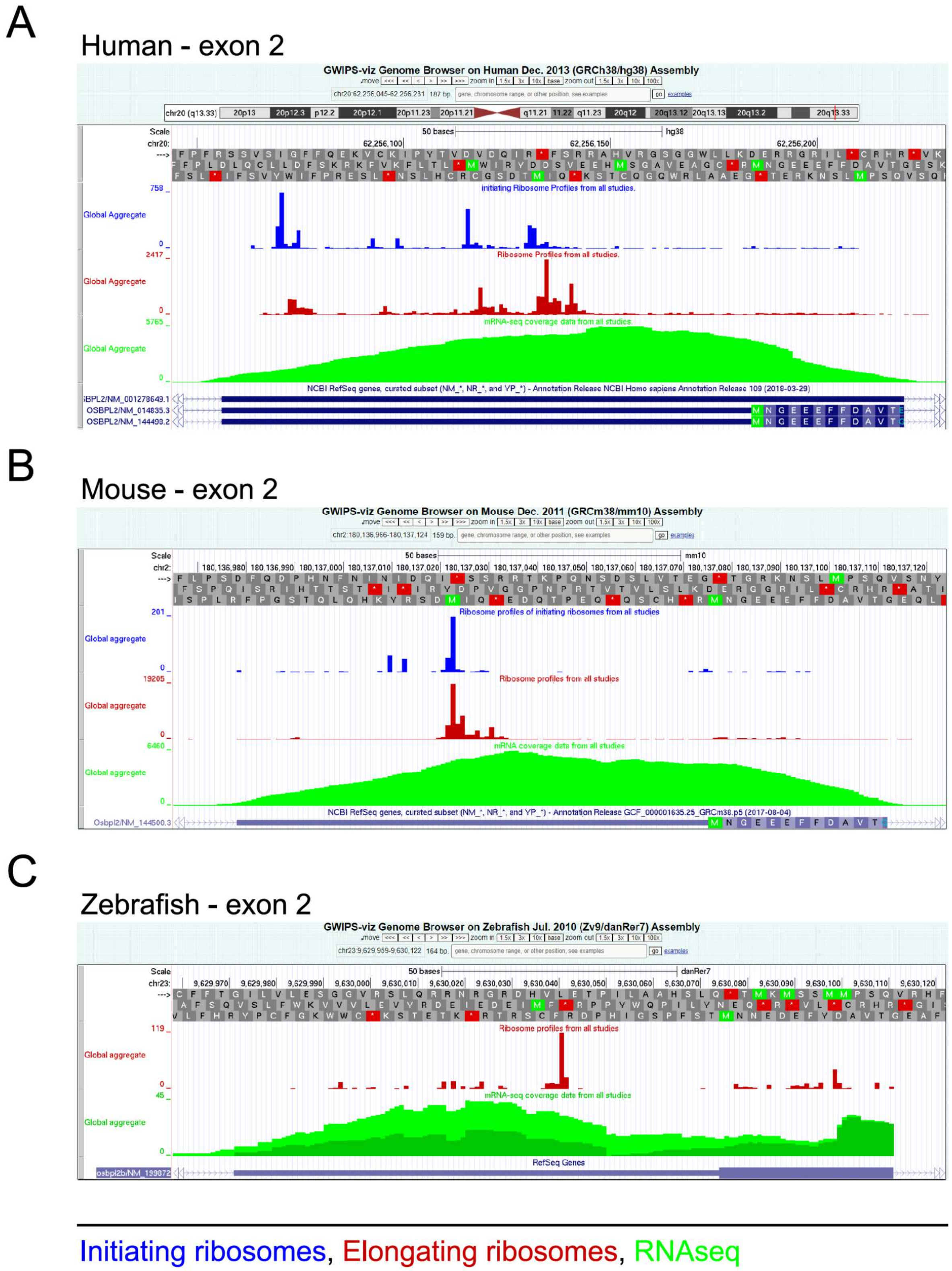

Figure S10. Apparent translation upstream of canonical initiation site of transcripts corresponding to human OSBPL2, mouse Osbpl2, and zebrafish osbpl2b. Tracks for window position, sequence translation, initiating ribosomes (P-sites), elongating ribosomes (A-sites), mRNAseq reads, and reference transcripts

are shown. For each species, strong peaks are present upstream of the canonical translation initiation sites. Apparent translation initiation at sites upstream of stop codons implies translation of short uORFs and potential regulation of the translation of the main ORFs. Data are aggregated from multiple studies. (A) Human OSBPL2 exon 2. (B) Mouse Osbpl2 exon 2. (C) Zebrafish osbpl2b. Screenshots taken from GWIPS-viz (2), https://gwips.ucc.ie/, July 2023.

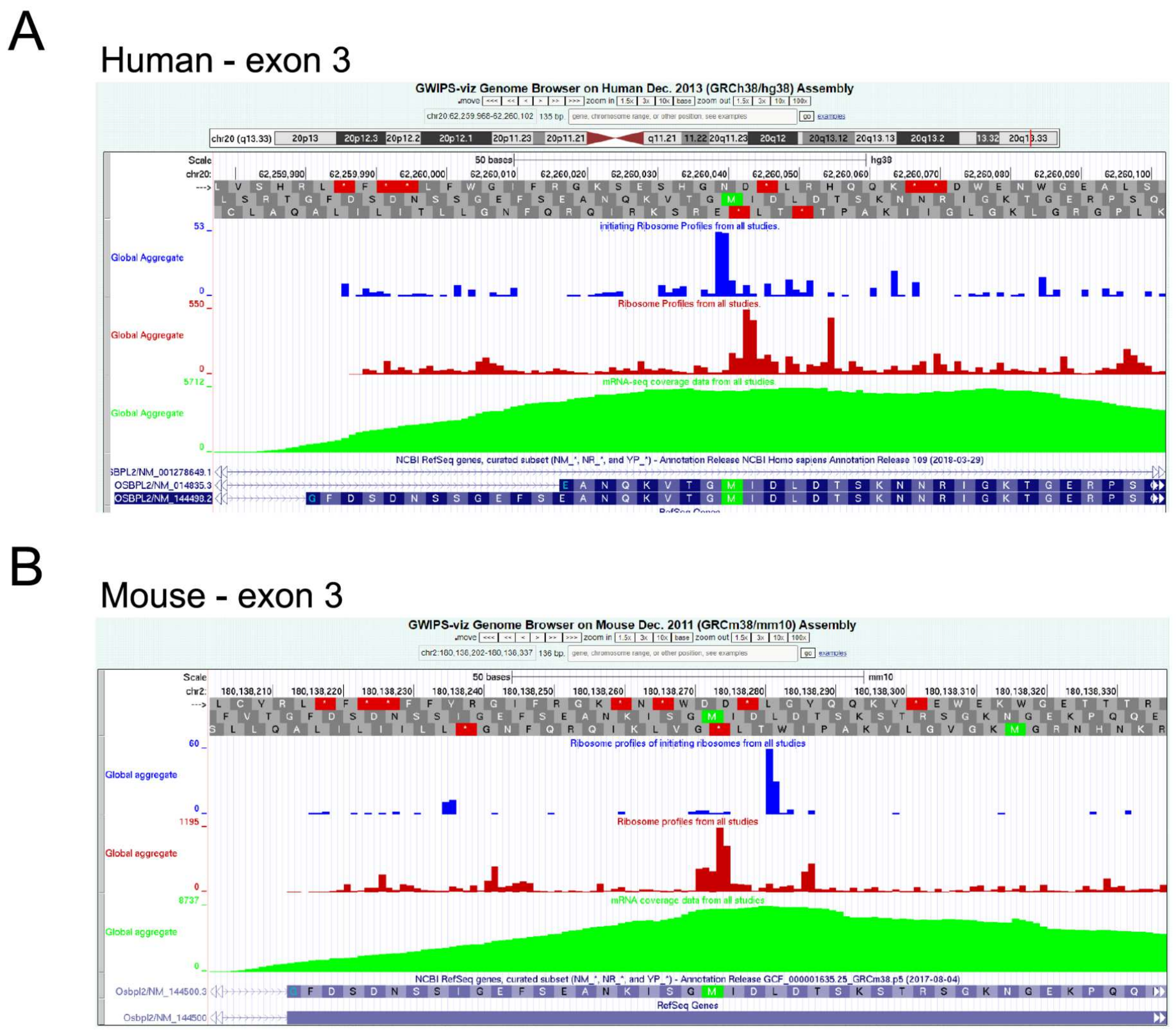

Initiating ribosomes, Elongating ribosomes, RNAseq

Figure S11. Apparent translation-initiation downstream of canonical initiation site in transcripts corresponding to human OSBPL2 and mouse Osbpl2. Tracks for window position, sequence translation, initiating ribosomes (P-sites), elongating ribosomes (A-sites), mRNAseq reads, and reference transcripts are shown. (A) Human OSBPL2 exon 3. Signal from initiating ribosomes is consistent with initiation at a codon corresponding to Met33 of the canonical transcript NM_144498.4. (B) Mouse Osbpl2 exon 3. Signal from initiating ribosomes is consistent with initiation at or near to a codon corresponding to Met33 of the canonical transcript NM_144500.4. Data are aggregated from multiple studies. Screenshots taken from GWIPS-viz (2), https://gwips.ucc.ie/, July 2023. Human or mouse ORP2 protein translated from Met33 would have an approximate molecular weight of 52kDa. Consistent with its being expressed physiologically, a 51kDa ORP2 band is observed upon Western

blotting of cells transfected with human ORP2 cDNA, and also upon blotting of a panel of mouse tissues (3).

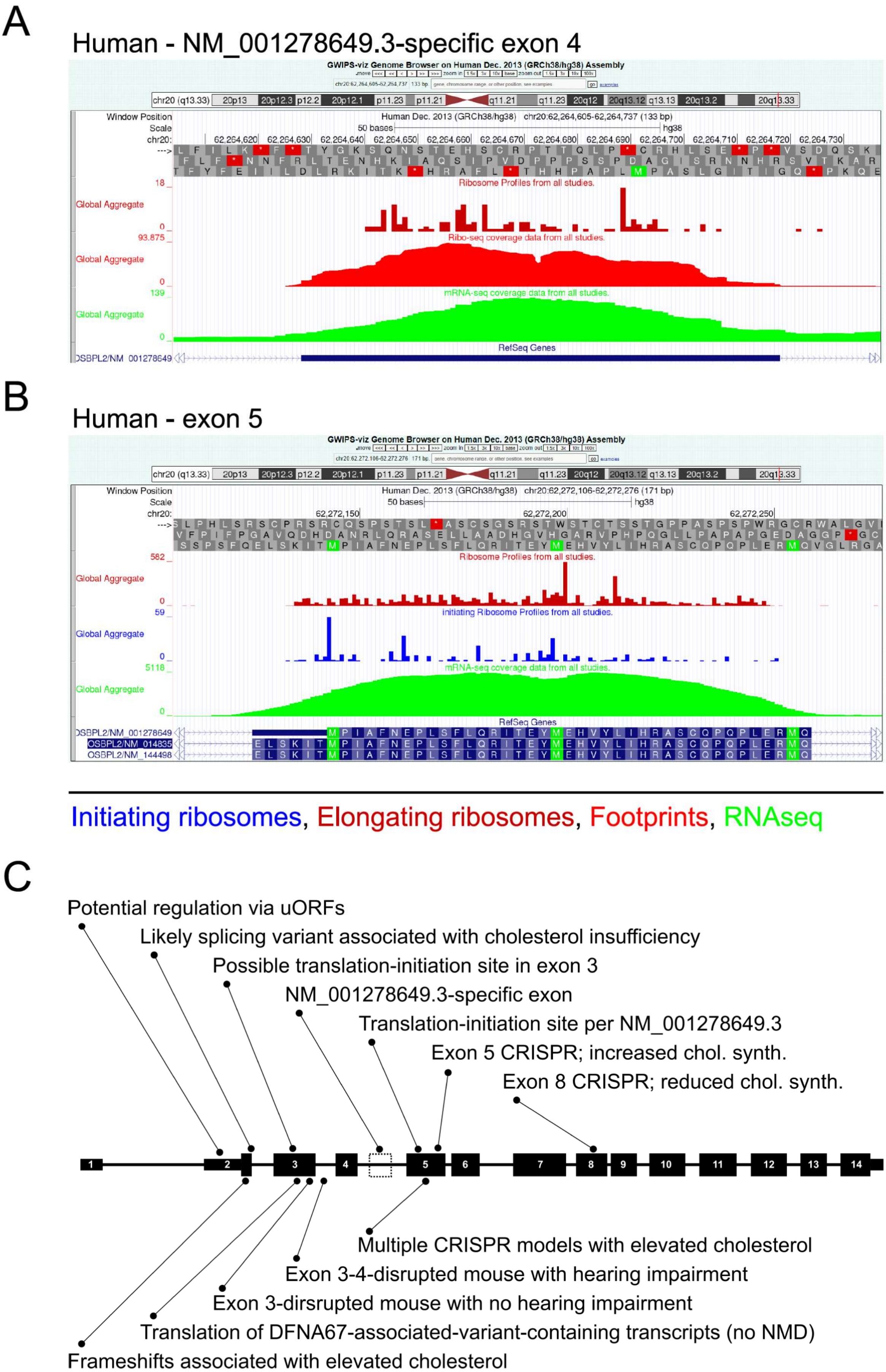
A
Human - NM_001278649.3-specific exon 4
GWIPS-viz Genome Browser on Human Dec. 2013 (GRCh38/hg38) Assembly
Ribosome Profiles from all studies.
Global Aggregate
Ribo-seq coverage data from all studies.
mRNA-seq coverage data from all studies.
RefSeq Genes
B
Human - exon 5
GWIPS-viz Genome Browser on Human Dec. 2013 (GRCh38/hg38) Assembly
Ribosome Profiles from all studies.
initiating Ribosome Profiles from all studies.
mRNA-seq coverage data from all studies.
RefSeq Genes
Initiating ribosomes, Elongating ribosomes, Footprints, RNAseq
C
Potential regulation via uORFs
Likely splicing variant associated with cholesterol insufficiency
Possible translation-initiation site in exon 3
NM_001278649.3-specific exon
Translation-initiation site per NM_001278649.3
Exon 5 CRISPR; increased chol. synth.
Exon 8 CRISPR; reduced chol. synth.
1
2
3
4
5
6
7
8
9
10
11
12
13
14
Multiple CRISPR models with elevated cholesterol
Exon 3-4-disrupted mouse with hearing impairment
Exon 3-dirsrupted mouse with no hearing impairment
Translation of DFNA67-associated-variant-containing transcripts (no NMD)
Frameshifts associated with elevated cholesterol

Figure S12. Apparent translation-initiation in exon 5 of human OSBPL2. (A) and (B) show ribosome profiling data from the GWIPS-viz resource (2), https://gwips.ucc.ie/, screenshots taken July 2023. Tracks for window position, sequence translation, initiating ribosomes (P-sites), elongating ribosomes (A-sites), elongating ribosomes (footprints), mRNAseq reads, and reference transcripts are shown. (A) Low-level signal is evidence for physiological existence and ribosome-engagement of mRNA containing exon ENSE00003640537 which is associated with the alternative OSBPL2 transcript NM_001278649.3. (B) Signal from initiating ribosomes is consistent with translation initiation at a codon corresponding to the annotated AUG start site of the alternative OSBPL2 transcript NM_001278649.3, within exon 5 of the canonical OSBPL2 transcript NM_144498.4. Notably, exon 5 contains three in-frame Met codons. (C) Summary of evidence for regulation of OSBPL2 protein at levels of translation and alternative splicing. From left to right: ribosome profiling data suggest potential regulation of translation by uORFs (see this study, Figure S9); CRISPR models generated through introduction of frameshift variants into exon 2 are associated with elevated cholesterol (4, 5); in contrast, a probable exon 2-splicing-mutant CRISPR model is associated with apparent cholesterol insufficiency (6); ribosome profiling data are consistent with some translation-initiation in exon 3 in human and mouse (see this study, Figure S10) and this is also consistent with a 51kDa ORP2 band observed upon Western blotting of cells transfected with human ORP2 cDNA, and a panel of mouse tissues (3); human DFNA67-disease-associated frameshift variants are not associated with nonsense-mediated decay (NMD) of transcript (7); a mouse model in which exon 3 is disrupted with CRISPR-induced frameshifts does not exhibit hearing impairment (7); whereas a model in which exons 3-4 are excised does (8); ribosome profiling data are consistent with physiological existence and ribosome-engagement of mRNA containing an exon specific to an alternative transcript, NM_001278649.3 (see Figure S11A, above); ribosome profiling data are consistent with low-level translation initiation at the annotated NM_001278649.3 start-site in exon 5 (see Figure S11B, above); multiple CRISPR models in which exon 5 is disrupted are associated with elevated cholesterol (see this study, Table S6); disruption of exon 5 in this study produced increased cholesterol biosynthesis (see Figure 5H); whilst disruption of exon 8 produced reduced cholesterol biosynthesis (see Figure 5H-J).

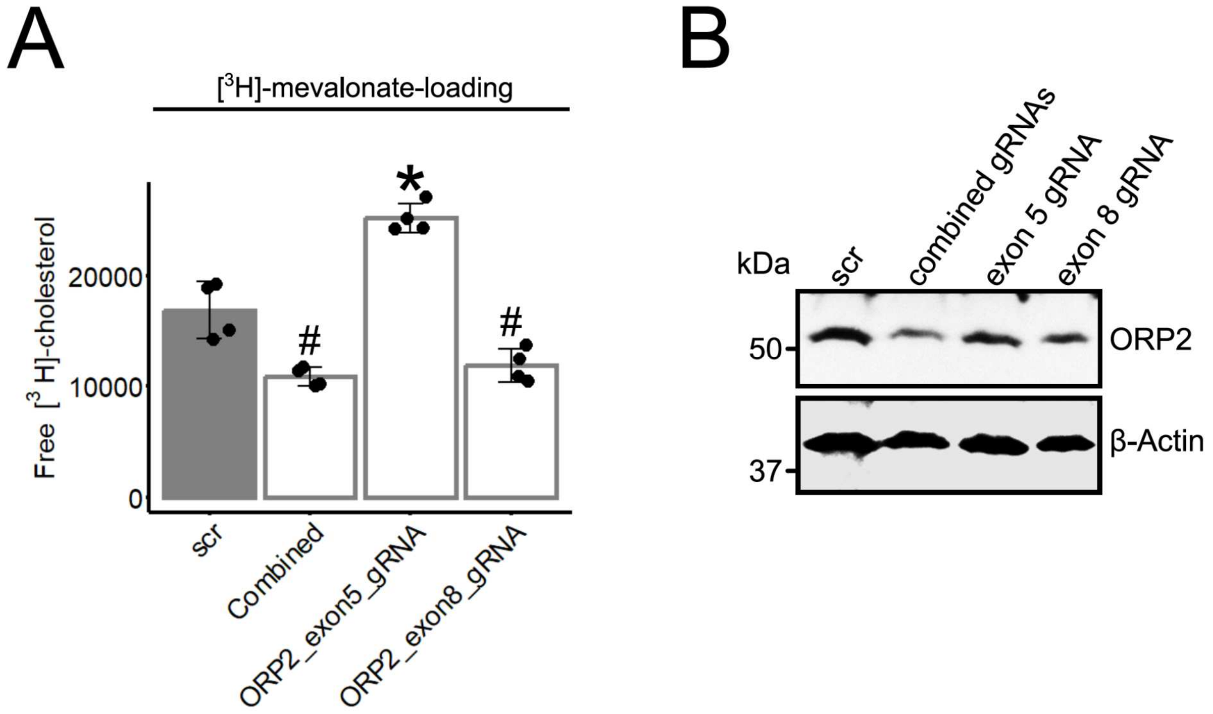

Figure S13. Reduced cholesterol biosynthesis upon simultaneous disruption of *OSBPL2* exons 5 and 8. (A) Bar graph to show incorporation of [^{3}H]-mevalonate into cholesterol in HEK293 cells transduced with lentivirus constructs. Cells transduced with the indicated constructs were selected with puromycin for at least 7 days, grown in media supplemented with LPDS for 24 hours, then incubated with 5µCi/well [^{3}H]-mevalonate for 24 hours. TLC was used to separate free cholesterol and radioactivity was quantified by scintillation counting (n=4; mean±SD). Data are representative of three replicate experiments. (B) Western blotting to show levels of full-length OSBPL2 expression in cells transduced with the indicated lentivirus constructs. Prior to sampling, cells were selected with puromycin for at least 7 days. $^{\#}p<0.05$, $^{*}p<0.01$.

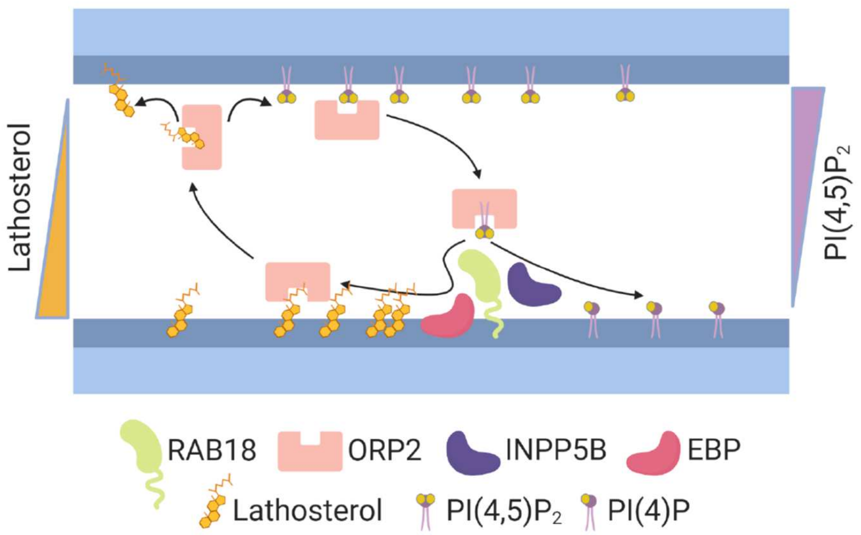

Figure S14. Model for lathosterol mobilization mediated by RAB18. ORP2 binds $PI(4,5)P_2$ on an apposed membrane. RAB18 interacts with ORP2 and INPP5B promoting the hydrolysis of $PI(4,5)P_2$ to PI(4)P and maintaining a $PI(4,5)P_2$ concentration gradient. RAB18 coordinates the biosynthesis of lathosterol by EBP and subsequent lathosterol mobilization by ORP2.

SUPPLEMENTARY MATERIALS AND METHODS

BirA/BioID proximity labelling (T-REx-293 cells)

The T-REx-293 Cell Lines (described above) were seeded onto 3x 15cm plates each and allowed to adhere. Expression of BirA*-RAB18 fusion proteins was induced by treatment with 20ng/ml Tetracycline for 16 hours. Media was then replaced with media containing 20% FBS, 20 ng/ml Tetracycline and 50 uM Biotin and the cells were incubated for a further 8 hours, washed with warmed PBS and pelleted in ice-cold PBS. Cell pellets were snap-frozen and stored at -80ºC prior to lysis. Lysis was carried out in 3ml of ice-cold RIPA buffer (150 mM NaCl, 1% NP40, 0.5% Sodium Deoxycholate, 0.1% SDS, 1mM EDTA, 50mM Tris, pH 7.4) supplemented with complete-mini protease inhibitor cocktail (Roche, Basel, Switzerland), 1mM PMSF, and 62.5 U/ml Benzonase (Merck). Lysates were incubated for 1 hour at 4ºC then sonicated in an ice bath (four 10 second bursts on low power). They were then clarified by centrifugation, and the supernatants transferred to tubes containing pre-washed streptavidin-sepharose (30µl bed-volume)(Merck). The beads were incubated for 3 hours at 4ºC, then washed five times in RIPA buffer and four times in buffer containing 100mM NaCl, 0.025% SDS and 25 mM Tris, pH7.4.

Preparation of cell lysates for label-free quantitative proteomics

RPE1 and HeLa cells were grown to confluence in T75 flasks. They were then trypsinised, and cell pellets were washed with PBS and snap-frozen prior to use. RPE1 pellets were resuspended in 300µl 6M GnHCl, 75mM Tris, pH=8.5. HeLa pellets were resuspended in 300µl 8M urea, 75mM NaCl, 50mM Tris, pH=8.4. In each case, samples were sonicated for 10 minutes using a Bioruptor device together with protein extraction beads (Diagenode). RPE1 samples were heated for 5 minutes at 95ºC. Samples were clarified by centrifugation.

Mass spectrometry

Washed beads from BioID experiments with T-Rex-293 cell lines were resuspended in 50µl 6M urea, 2M thiourea, 10mM Tris, pH=8.5 and DTT was added to 1mM. After

30 minutes incubation at 37ºC, samples were alkylated with 5mM iodoacetamide (IAA) in the dark for 20minutes. DTT was increased to 5mM and 1µg lysC was added, then samples were incubated at 37ºC for 6 hours. Samples were diluted to 1.4M urea, then digested with trypsin (Promega), overnight at 37°C, according to manufacturer's instructions. Samples were acidified by the addition of 0.9% formic acid and 5% acetonitrile. LC-MS was carried out as previously described (Brunet et al., 2016). Briefly, peptides in an aqueous solution containing 5% acetonitrile and 0.1% formic acid were loaded onto a 3 µm PepMap100, 2 cm, 75 µm diameter sample column using an Easy nLC 1000 ultrahigh pressure liquid chromatography system (ThermoFisher). They were eluted with acetonitrile/formic acid into an in-line 50 cm separating column (2 µm PepMap C18, 75 µm diameter) at 40°C. Separated peptides were ionized using an Easy Spray nano source and subjected to MS/MS analysis using a Velos Orbitrap instrument (ThermoFisher). One set of samples was used for the BioID-RAB18 experiment in T-Rex-293 cells (Figure S3, Table S2).

Following acquisition, data were analysed using SEAQUEST software. A NeXprot Human database with 20379 entries was searched. No missed cleavages were permitted. Fixed modification by carbamidomethylation of cysteine residues was considered. Variable modification by oxidation or hydroxylation of methionine residues was considered. Mass tolerance for precursor ions was ±2m/z and that for fragment ions was ±1m/z. Thresholds for accepting individual spectra were set at $p<0.05$. A %FDR of 0.25% was calculated using the PeptideProphet package (http://peptideprophet.sourceforge.net/). Single-peptide identifications of proteins were removed.

RPE1 lysates were reduced and alkylated through addition of tris(2-carboxyethyl)phosphine (TCEP) and 2-chloroacetamide (CAA) to 5mM and 10mM respectively and then incubated at 95°C for 5 minutes. After cooling, samples were diluted to 3M guanidine and 0.5µg lysC added with incubation overnight at 37°C. A further dilution to 1M guanidine was followed by digest with 0.3µg trypsin at 37°C for 4 hours. Samples were acidified with TFA. HeLa lysates were reduced and alkylated by addition of DTT to 10mM, then by addition of IAA to 25mM, then further addition of DTT to 25mM, with incubation at room temperature for 30-60 minutes following each step. Samples were digested with lysC, overnight at 37°C. They were then diluted to 2M urea, and further digested, overnight at 37°C. Samples were acidified

with TFA. Trypsin cleaves on the C-terminal side of lysine and arginine residues unless the C-terminal residue is proline. Hydrolysis is slower where the C-terminal residue is acidic. Lys-C cleaves on the C-terminal side of lysine residues. Peptides were loaded on to activated (methanol), equilibrated (0.1% TFA) C18 stage tips before being washed with 0.1% TFA and eluted with 0.1% TFA/80 acetonitrile. The organic was dried off, 0.1% TFA added to 15 µl and 5 µl injected onto LC-MS. Peptides were separated on an Ultimate nano HPLC instrument (ThermoFisher), and analysed on either an Orbitrap Lumos or a Q Exactive Plus instrument (ThermoFisher).

Three sets of replicate samples were used to generate the RPE1 quantitative proteomics dataset (Figure S4, Table S3). Each set of samples was grown and harvested independently. Six sets of replicate samples were used to generate the HeLa cell quantitative proteomics dataset (Table S4). Two different wild-type clones and two different TBC1D20-null genotypes were used (three replicates each). These can be considered biological replicates.

After data-dependent acquisition of HCD fragmentation spectra, data were analysed using MaxQuant (version 1.6.2.10 for the RPE1 experiment and version 1.5.7.4 for the HeLa cell experiment). For the RPE1 experiment, the Uniprot Human 2018_07 database with 21050 entries was searched. For the HeLa cell experiment, the Uniprot Human 2017_01 database with 21031 entries was searched. 2 missed/non-specific cleavages were permitted. Fixed modification by carbamidomethylation of cysteine residues was considered. Variable modification by oxidation of methionine residues and N-terminal acetylation were considered. Mass error was set at 20 ppm for the first search tolerance and 4.5 ppm main search tolerance. Thresholds for accepting individual spectra were set at $p<0.05$. Single-peptide identifications of proteins were removed. %FDR was estimated at <5% using the decoy search method. Additional parameters and gradients used for separation are provided in Table S7. Quantification data were produced with MaxLFQ (9). p values for comparisons between LFQ intensities of each protein in samples from test and wild-type genotypes were first calculated by Student's t-tests. p values were then adjusted for multiple testing using an online calculator (https://www.sdmproject.com/utilities/?show=FDR).

SUPPORTING REFERENCES

1. The FANTOM Consortium and the RIKEN PMI and CLST (DGT) (2014) A promoter-level mammalian expression atlas. *Nature*. **507**, 462–470
2. Michel, A. M., Fox, G., M. Kiran, A., De Bo, C., O'Connor, P. B. F., Heaphy, S. M., Mullan, J. P. A., Donohue, C. A., Higgins, D. G., and Baranov, P. V. (2014) GWIPS-viz: development of a ribo-seq genome browser. *Nucl. Acids Res.* **42**, D859–D864
3. Laitinen, S., Lehto, M., Lehtonen, S., Hyvärinen, K., Heino, S., Lehtonen, E., Ehnholm, C., Ikonen, E., and Olkkonen, V. M. (2002) ORP2, a homolog of oxysterol binding protein, regulates cellular cholesterol metabolism. *J Lipid Res*. **43**, 245–255
4. Wang, T., Wei, Q., Liang, L., Tang, X., Yao, J., Lu, Y., Qu, Y., Chen, Z., Xing, G., and Cao, X. (2020) OSBPL2 Is Required for the Binding of COPB1 to ATGL and the Regulation of Lipid Droplet Lipolysis. *iScience*. **23**, 101252
5. Zhang, C., Zhang, H., Zhang, M., Lin, C., Wang, H., Yao, J., Wei, Q., Lu, Y., Chen, Z., Xing, G., and Cao, X. (2019) OSBPL2 deficiency upregulate SQLE expression increasing intracellular cholesterol and cholesteryl ester by AMPK/SP1 and SREBF2 signalling pathway. *Experimental Cell Research*. **383**, 111512
6. Wang, H., Ma, Q., Qi, Y., Dong, J., Du, X., Rae, J., Wang, J., Wu, W.-F., Brown, A. J., Parton, R. G., Wu, J.-W., and Yang, H. (2019) ORP2 Delivers Cholesterol to the Plasma Membrane in Exchange for Phosphatidylinositol 4, 5-Bisphosphate (PI(4,5)P2). *Molecular Cell*. **73**, 458-473.e7
7. Koh, Y. I., Oh, K. S., Kim, J. A., Noh, B., Choi, H. J., Joo, S. Y., Rim, J. H., Kim, H.-Y., Kim, D. Y., Yu, S., Kim, D. H., Lee, S.-G., Jung, J., Choi, J. Y., and Gee, H. Y. (2022) *OSBPL2* mutations impair autophagy and lead to hearing loss, potentially remedied by rapamycin. *Autophagy*. **18**, 2593–2614
8. Shi, H., Wang, H., Zhang, C., Lu, Y., Yao, J., Chen, Z., Xing, G., Wei, Q., and Cao, X. (2022) Mutations in OSBPL2 cause hearing loss associated with primary cilia defects via sonic hedgehog signaling. *JCI Insight*. **7**, e149626
9. Cox, J., Hein, M. Y., Luber, C. A., Paron, I., Nagaraj, N., and Mann, M. (2014) Accurate proteome-wide label-free quantification by delayed normalization and maximal peptide ratio extraction, termed MaxLFQ. *Mol Cell Proteomics*. **13**, 2513–2526
