# Supplementary material for: Comparative proximity biotinylation implicates the small GTPase RAB18 in sterol mobilization and biosynthesis
Source: J Biol Chem. 2023 Sep 28;299(11):105295. doi: 10.1016/j.jbc.2023.105295 (PMC10641524; doi:10.1016/j.jbc.2023.105295)
Supplement: Single_peptide_identification [file mmc2.pdf]

# – single-peptide-identifications –

List of Figures

1 AHCY First experiment 5
2 AHCY Second experiment 5
3 AHCY Third experiment 6
4 ARHGDIA Second experiment 6
5 ARHGDIA Third experiment 7
6 ARID4A Second experiment 7
7 ARID4A Third experiment 8
8 ATL3 First experiment 8
9 ATL3 Second experiment 9
10 BABAM1 Second experiment 9
11 BABAM1 Third experiment 10
12 BET1L First experiment 10
13 BET1L Second experiment 11
14 BET1L Third experiment 11
15 c11orf49 First experiment 12
16 c11orf49 Second experiment 12
17 c11orf49 Third experiment 13
18 CAPG Second experiment 13
19 CAPG Third experiment 14
20 CHP1 Second experiment 14
21 CHP1 Third experiment 15
22 COL16A1 Second experiment 15
23 COL16A1 Third experiment 16
24 CROCC First experiment 16
25 CROCC Third experiment 17

26 CUL4B First experiment 17
27 CUL4B Third experiment 18
28 EI24 Second experiment 18
29 EI24 Third experiment 19
30 ELOVL5 First experiment 19
31 ELOVL5 Second experiment 20
32 ELOVL5 Third experiment 20
33 FAM101B First experiment 21
34 FAM101B Second experiment 21
35 FAM134B First experiment 22
36 FAM134B Second experiment 22
37 FAM134B Third experiment 23
38 GBA2 First experiment 23
39 GBA2 Second experiment 24
40 GBA2 Third experiment 24
41 GPR180 First experiment 25
42 GPR180 Second experiment 25
43 GPR180 Third experiment 26
44 GPR89B;GPR89A First experiment 26
45 GPR89B;GPR89A Second experiment 27
46 GPR89B;GPR89A Third experiment 27
47 HSPA4 Second experiment 28
48 HSPA4 Third experiment 28
49 ITGB6 First experiment 29
50 ITGB6 Second experiment 29
51 ITGB6 Third experiment 30
52 JAGN1 Second experiment 30
53 JAGN1 Third experiment 31
54 MOB3B First experiment 31

55 MOB3B Second experiment 32
56 MOB3B Third experiment 32
57 MPZL1 First experiment 33
58 MPZL1 Second experiment 33
59 MPZL1 Third experiment 34
60 OSBPL2 First experiment 34
61 OSBPL2 Second experiment 35
62 OSBPL2 Third experiment 35
63 PEX11B First experiment 36
64 PEX11B Second experiment 36
65 PEX11B Third experiment 37
66 PLEKHA1 First experiment 37
67 PLEKHA1 Second experiment 38
68 PLEKHA1 Third experiment 38
69 PRAF2 First experiment 39
70 PRAF2 Second experiment 39
71 PRAF2 Third experiment 40
72 PRR14L First experiment 40
73 PRR14L First experiment 41
74 RAB13 Second experiment 41
75 RAB13 Third experiment 42
76 RAB27A Second experiment 42
77 RAB27A Third experiment 43
78 RAP1A;RAP1B Second experiment 43
79 RAP1A;RAP1B Third experiment 44
80 RER1 Second experiment 44
81 RER1 Third experiment 45
82 RGPD3;RGPD4 Second experiment 45
83 RGPD3;RGPD4 Third experiment 46

84 SLC39A7 First experiment 46
85 SLC39A7 Second experiment 47
86 SLC39A7 Third experiment 47
87 SNX9 First experiment 48
88 SNX9 Third experiment 48
89 SPCS1 First experiment 49
90 SPCS1 Second experiment 49
91 SPCS1 Third experiment 50
92 TLCD1 First experiment 50
93 TLCD1 Third experiment 51
94 TMEM115 First experiment 51
95 TMEM115 Second experiment 52
96 TMEM115 Third experiment 52
97 TMEM161A Second experiment 53
98 TMEM161A Third experiment 53
99 TMEM245 Second experiment 54
100 TMEM245 Third experiment 54
101 TNPO1 First experiment 55
102 TNPO1 Second experiment 55
103 TNPO1 Third experiment 56
104 VAT1 Second experiment 56
105 VAT1 Third experiment 57

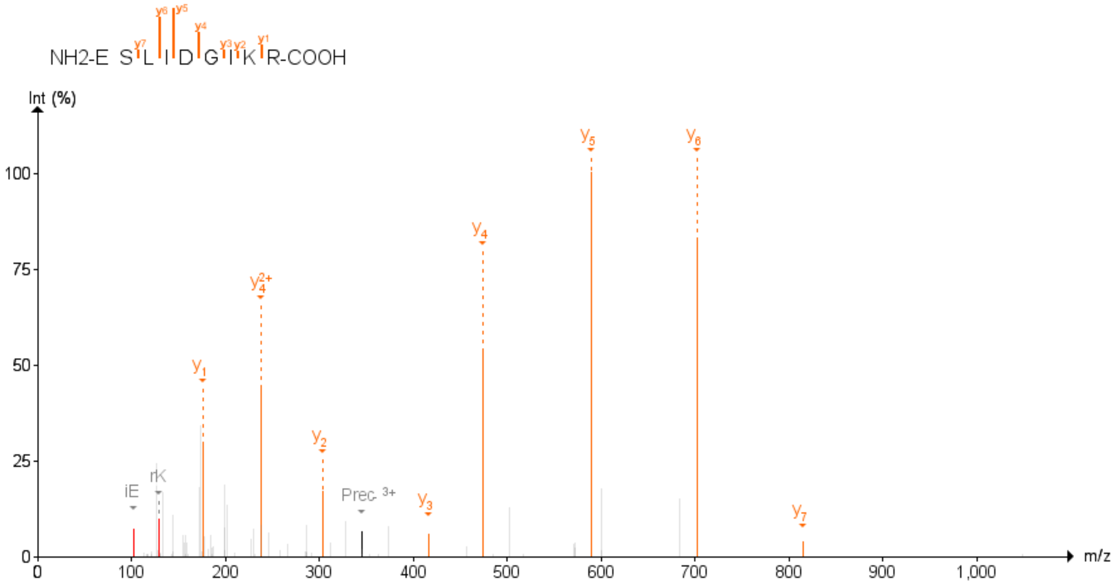

Figure 1: AHCY First experiment

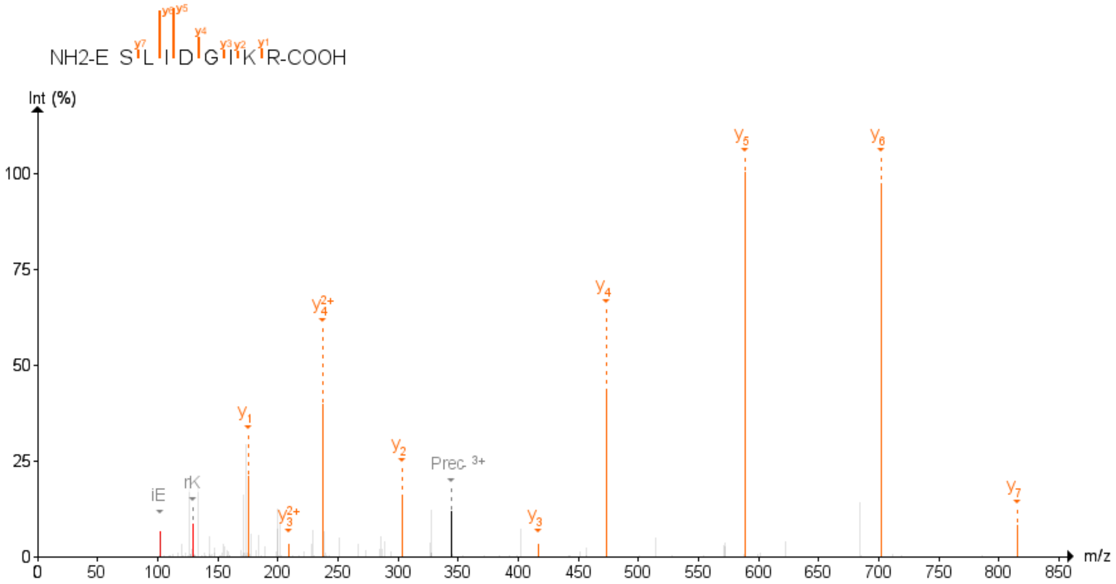

Figure 2: AHCY Second experiment

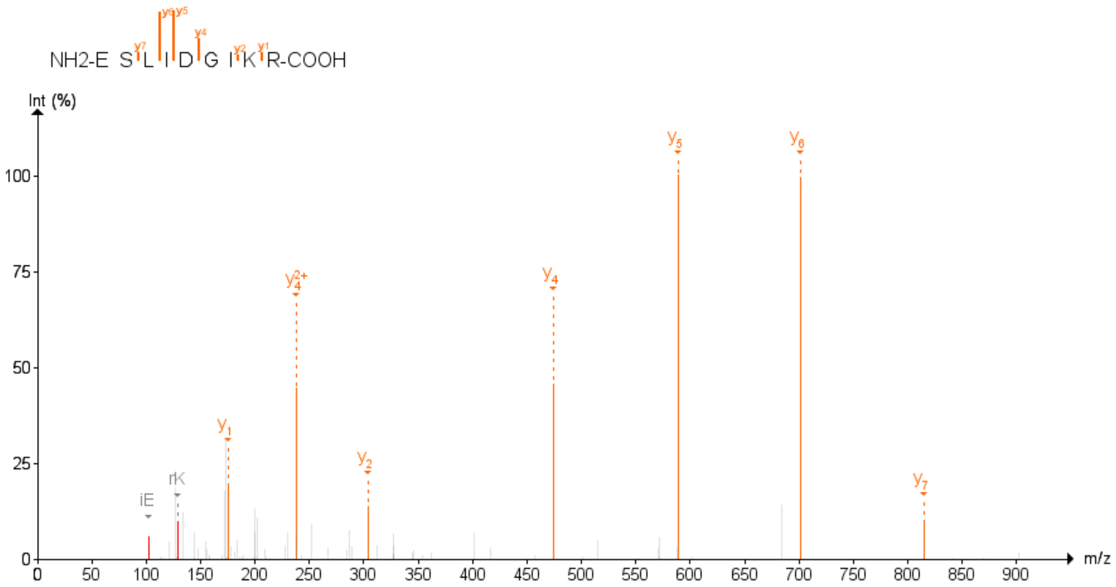

Figure 3: AHCY Third experiment

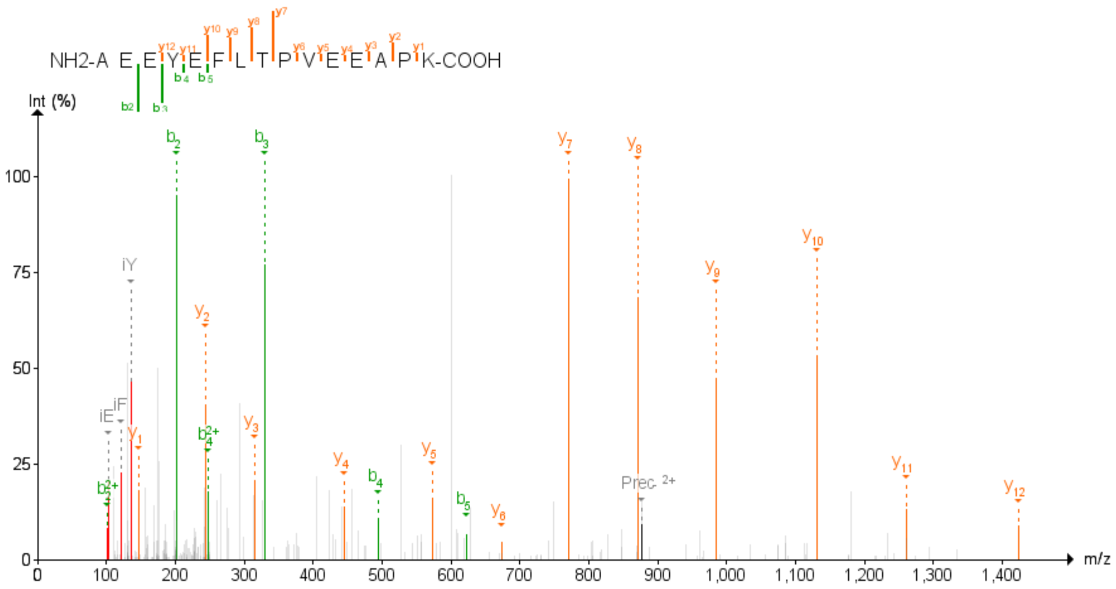

Figure 4: ARHGDIA Second experiment

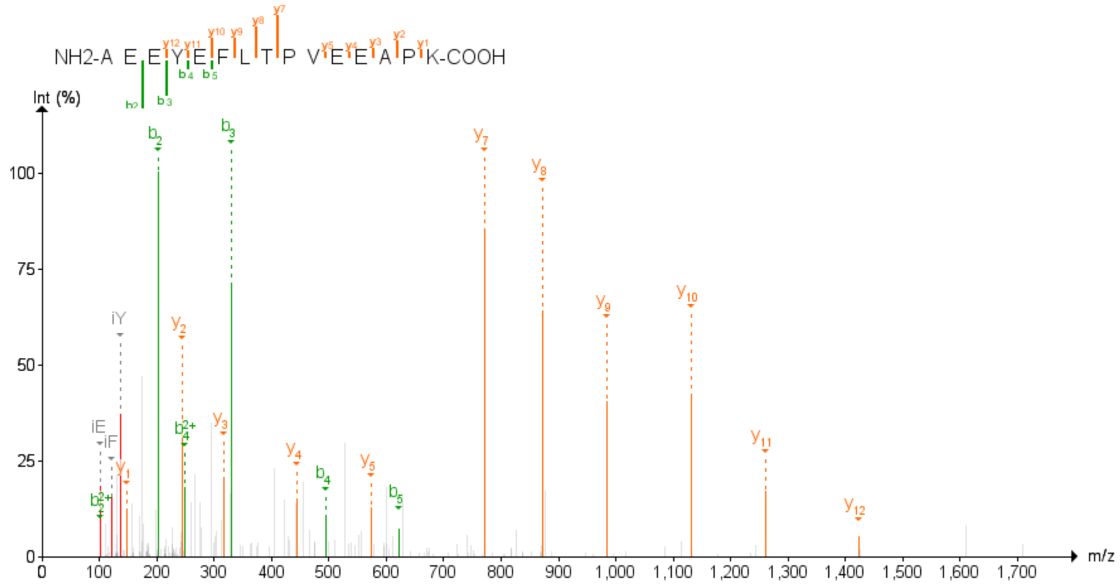

Figure 5: ARHGDIA Third experiment

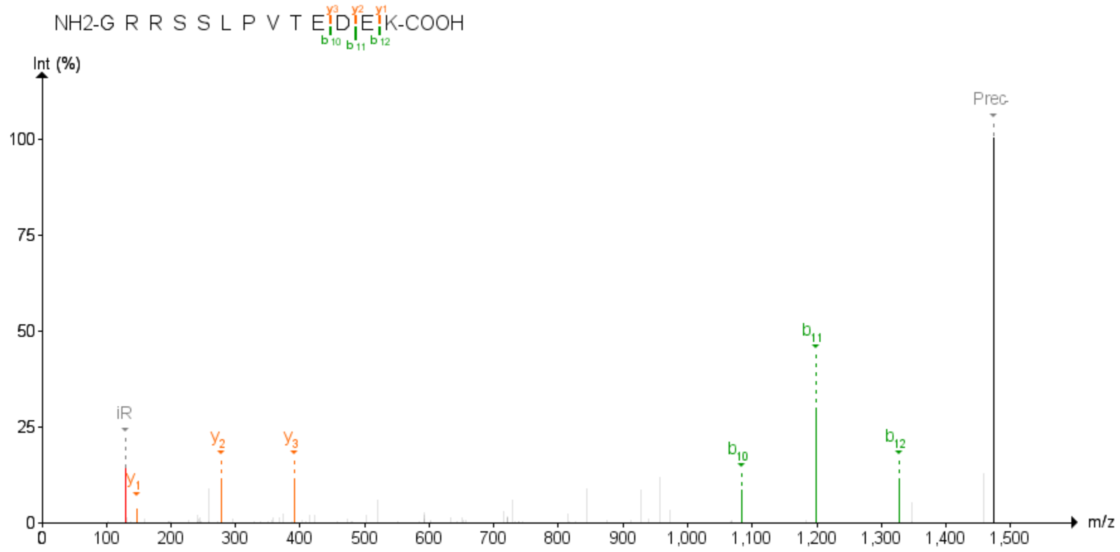

Figure 6: ARID4A Second experiment

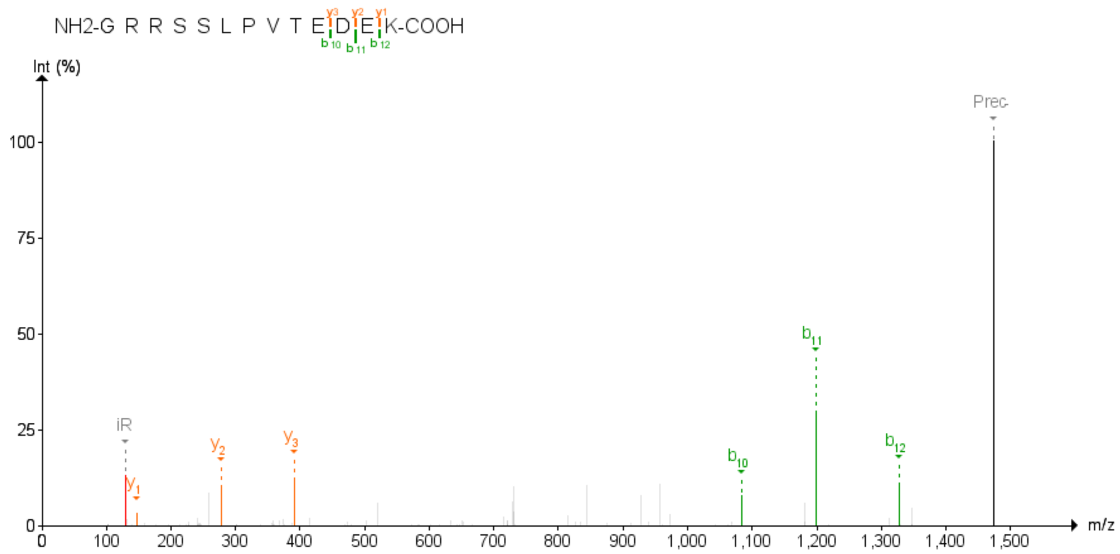

Figure 7: ARID4A Third experiment

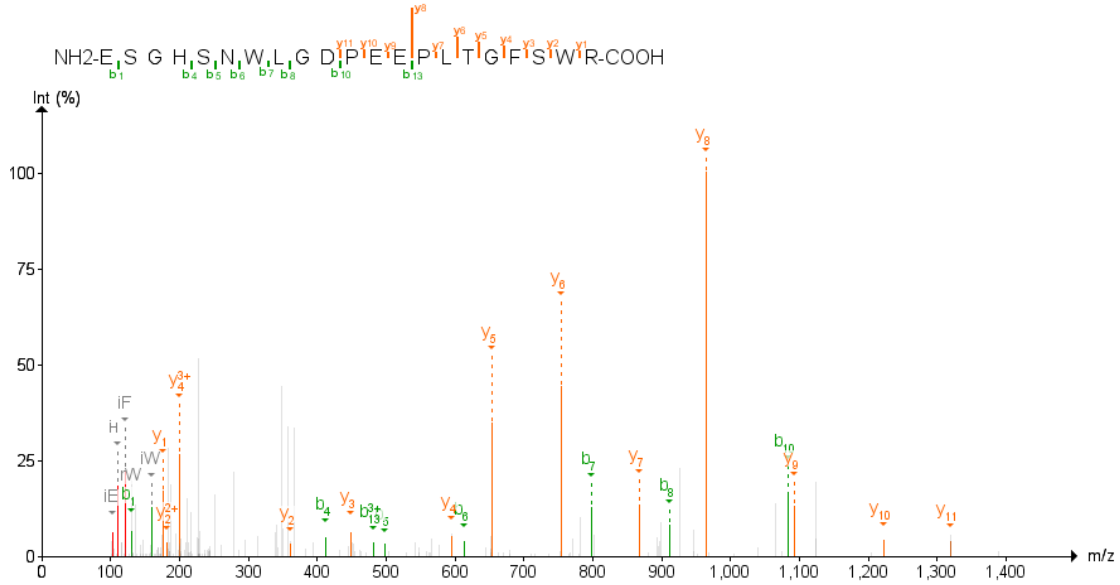

Figure 8: ATL3 First experiment

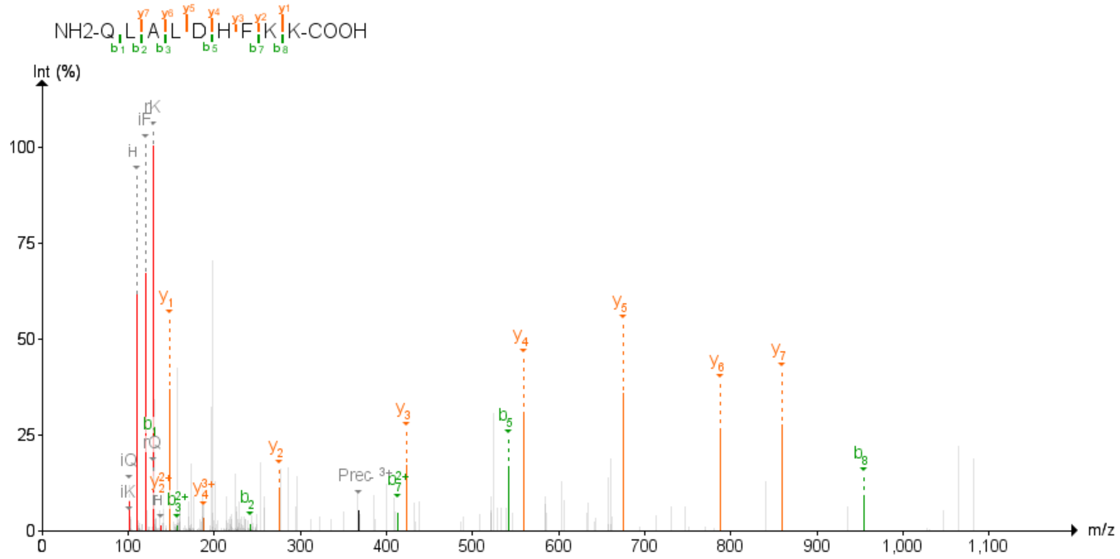

Figure 9: ATL3 Second experiment

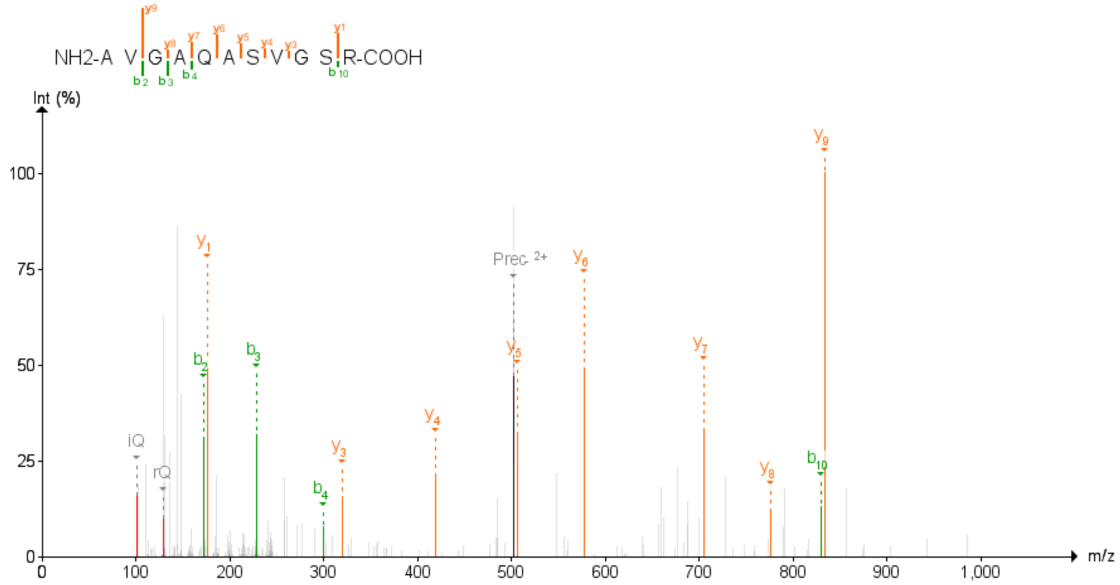

Figure 10: BABAM1 Second experiment

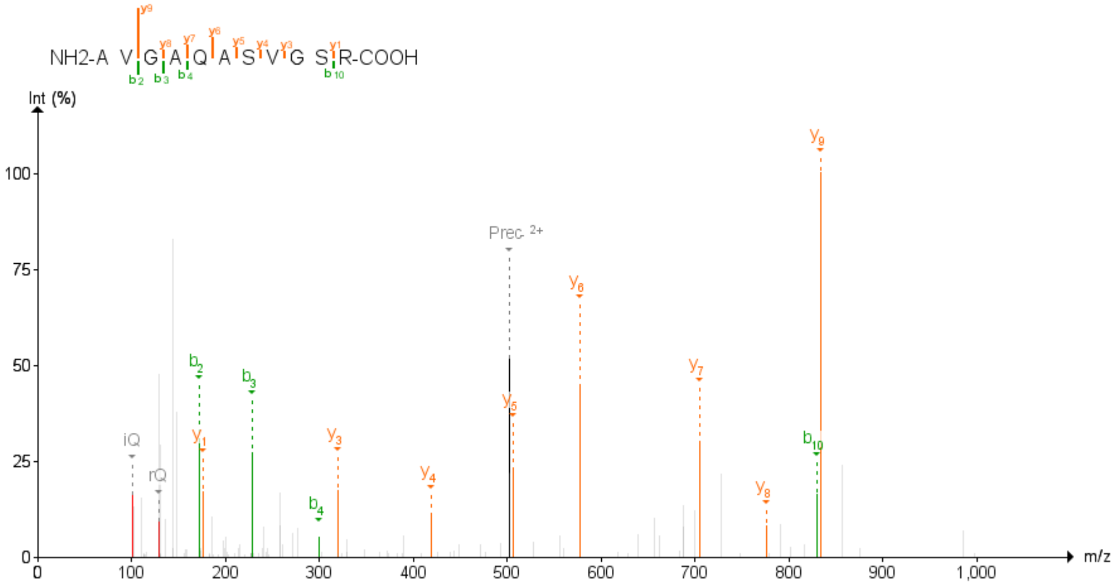

Figure 11: BABAM1 Third experiment

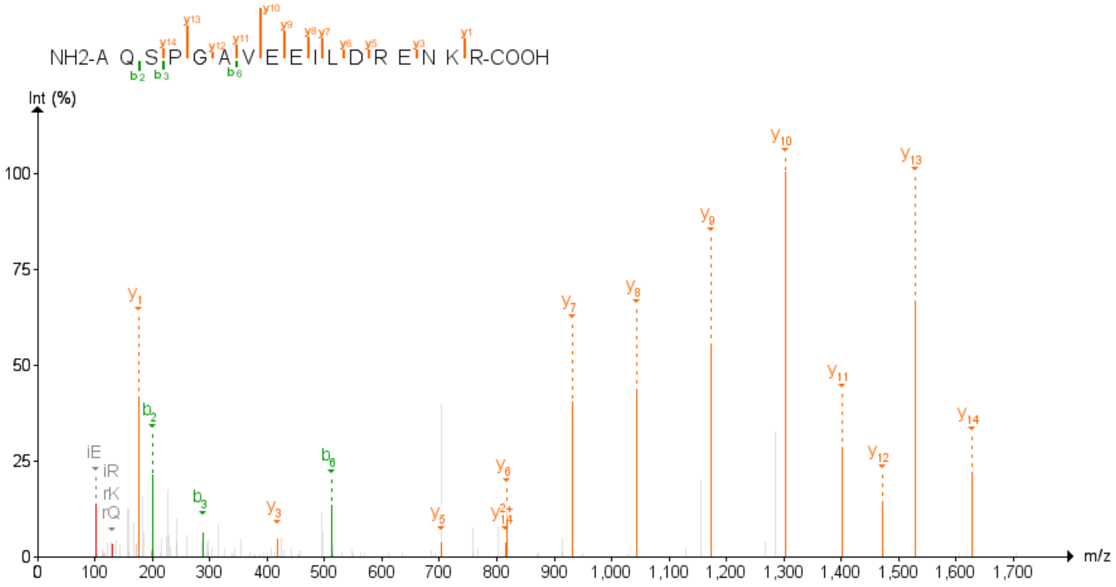

Figure 12: BET1L First experiment

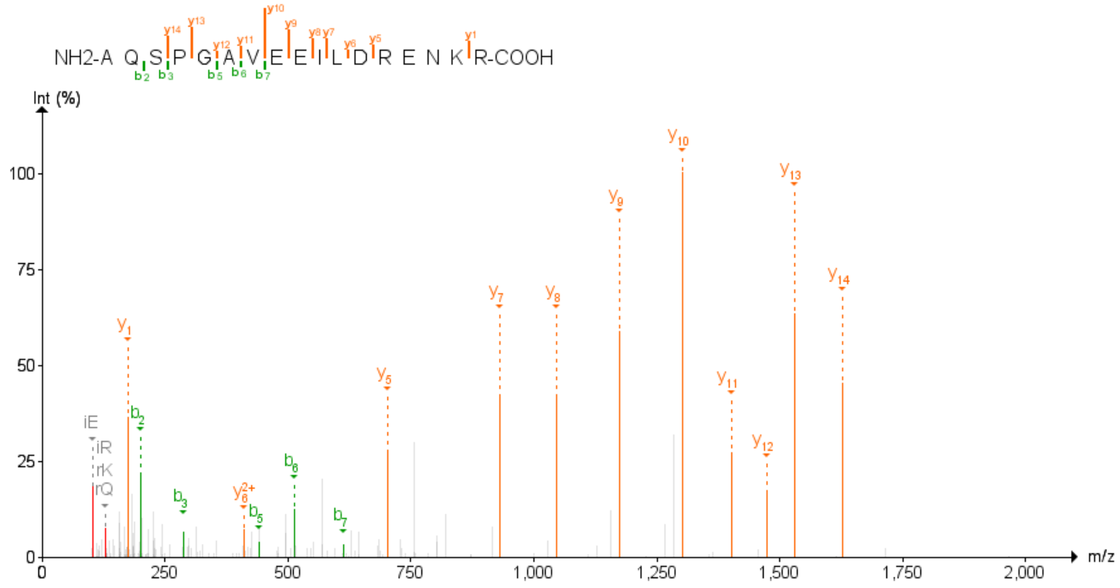

Figure 13: BET1L Second experiment

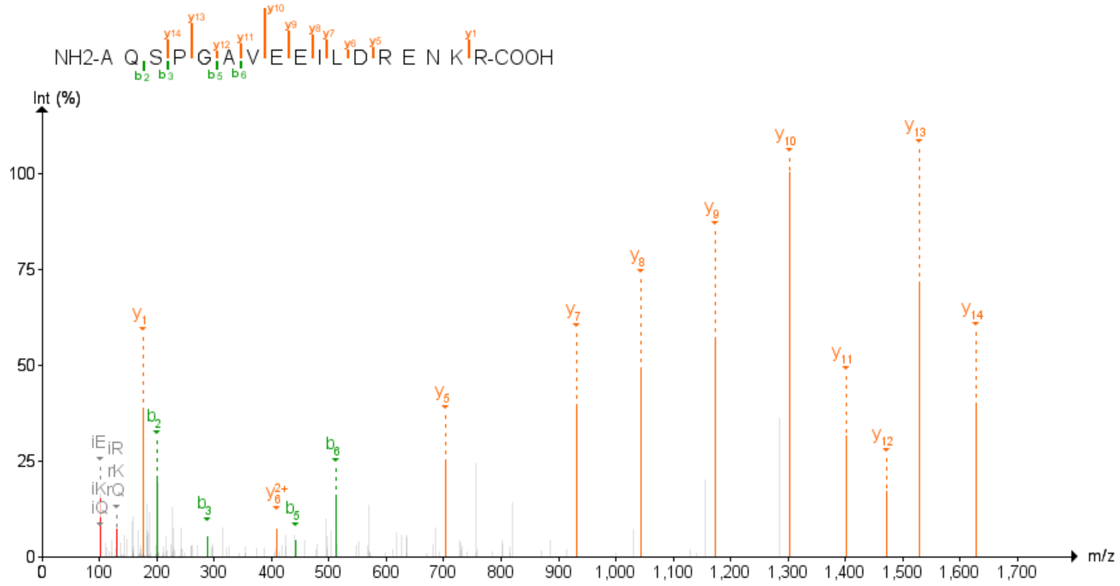

Figure 14: BET1L Third experiment

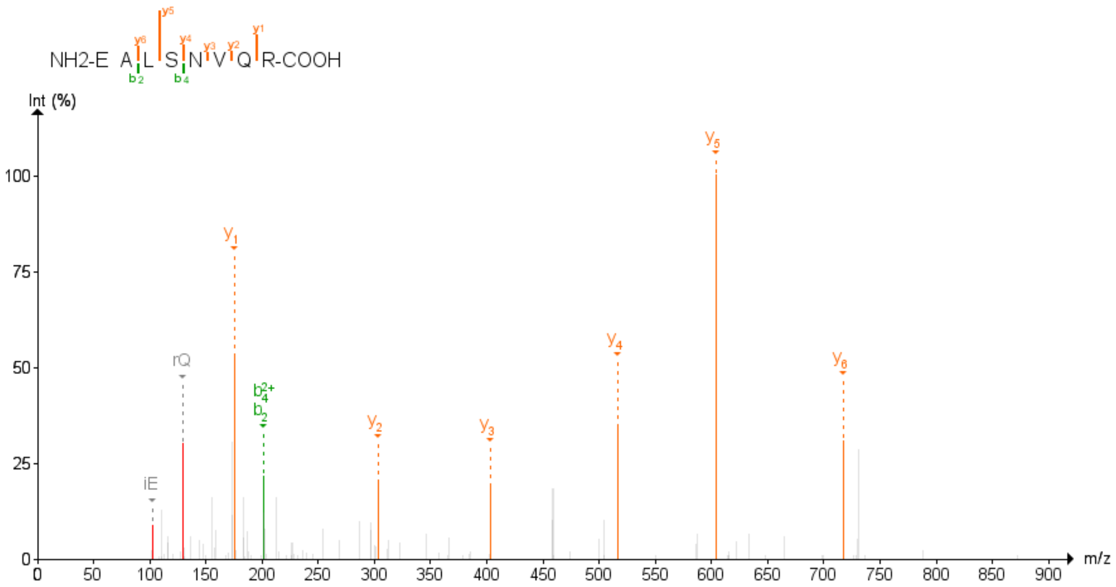

Figure 15: c11orf49 First experiment

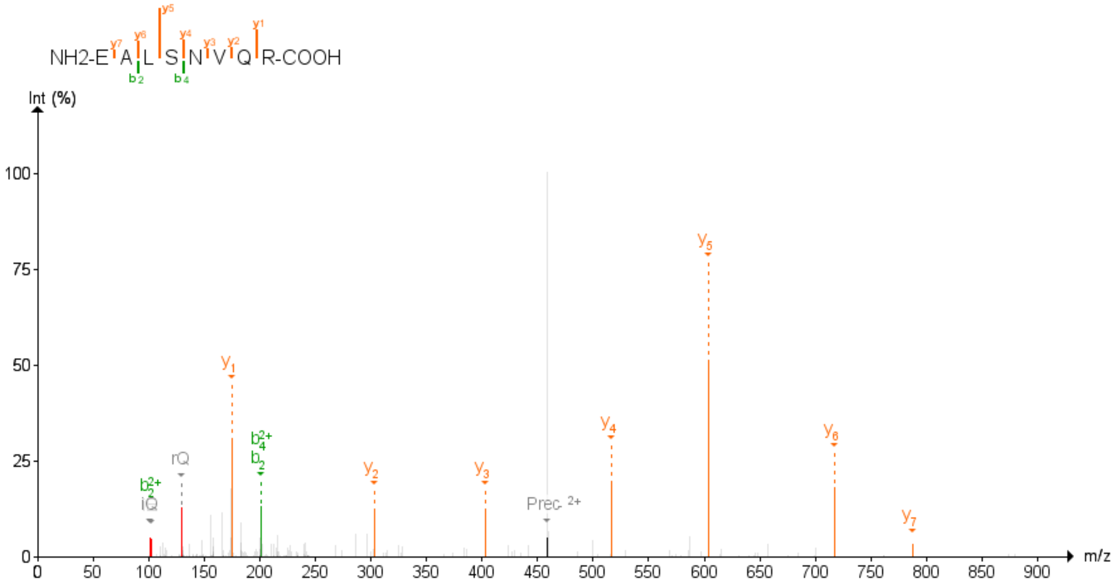

Figure 16: c11orf49 Second experiment

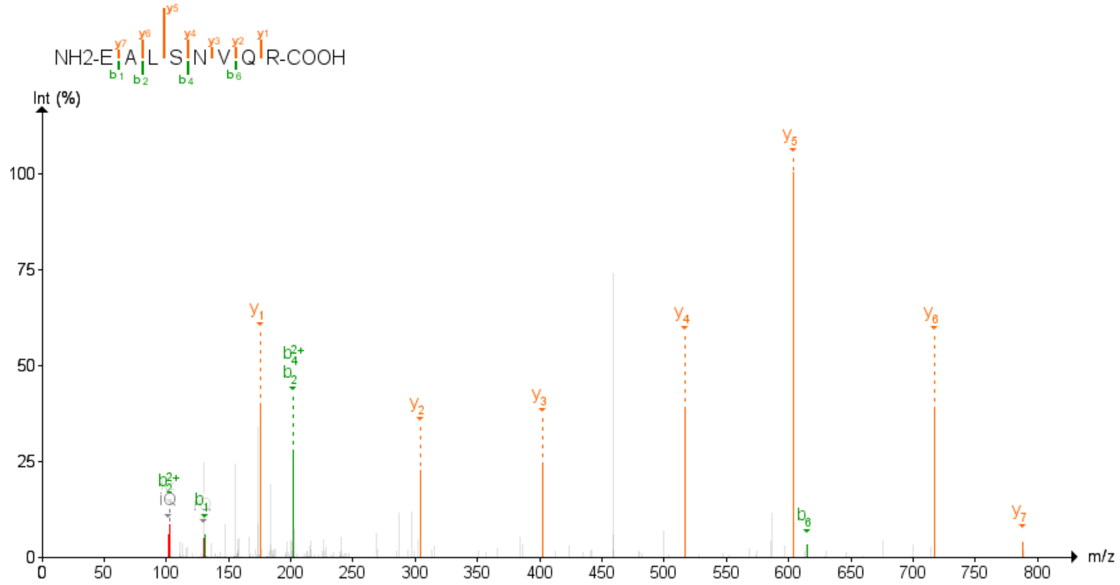

Figure 17: c11orf49 Third experiment

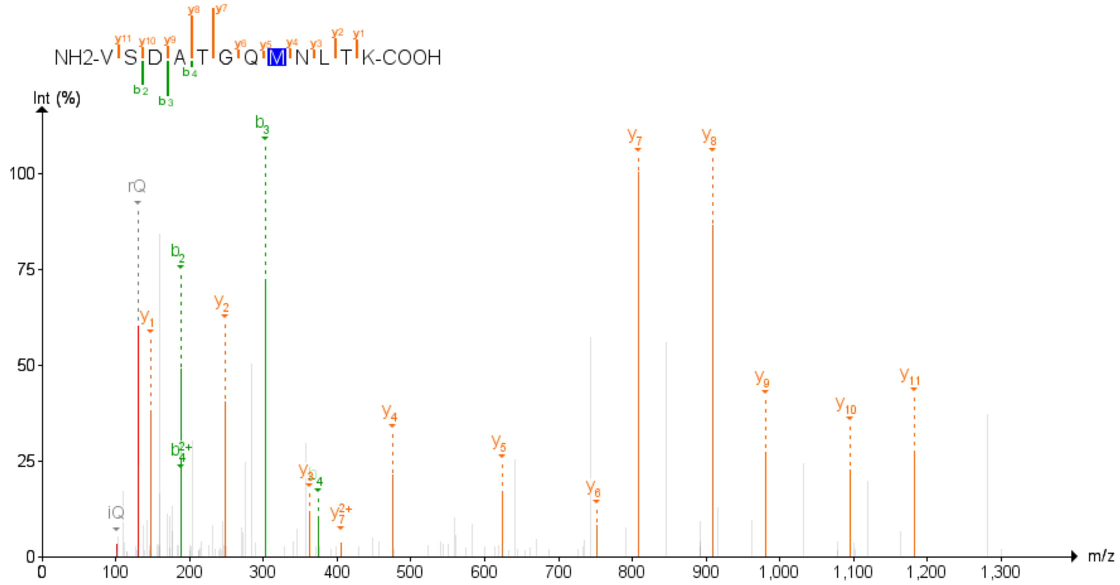

Figure 18: CAPG Second experiment

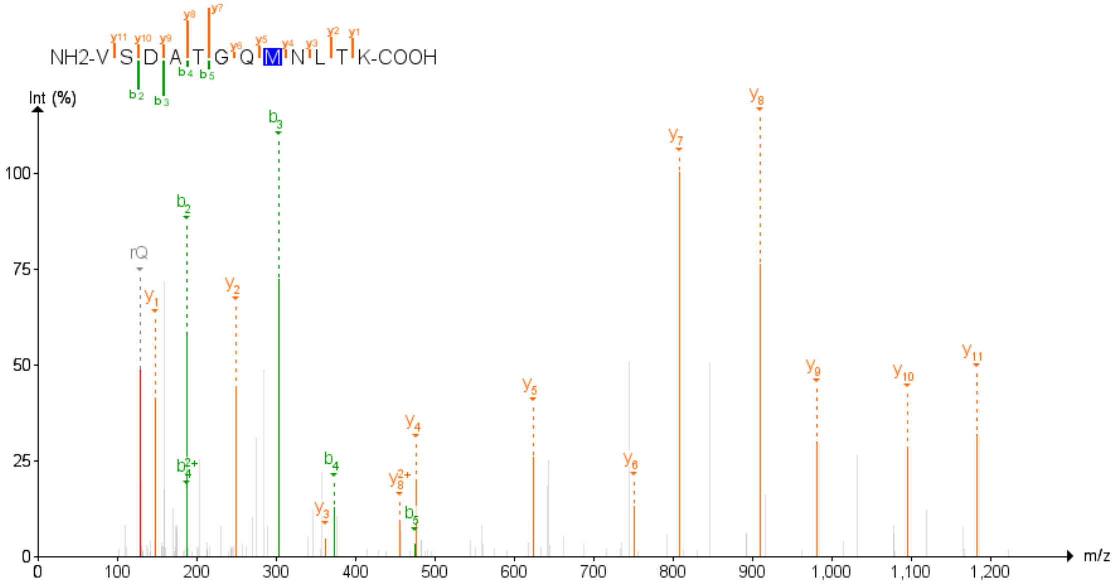

Figure 19: CAPG Third experiment

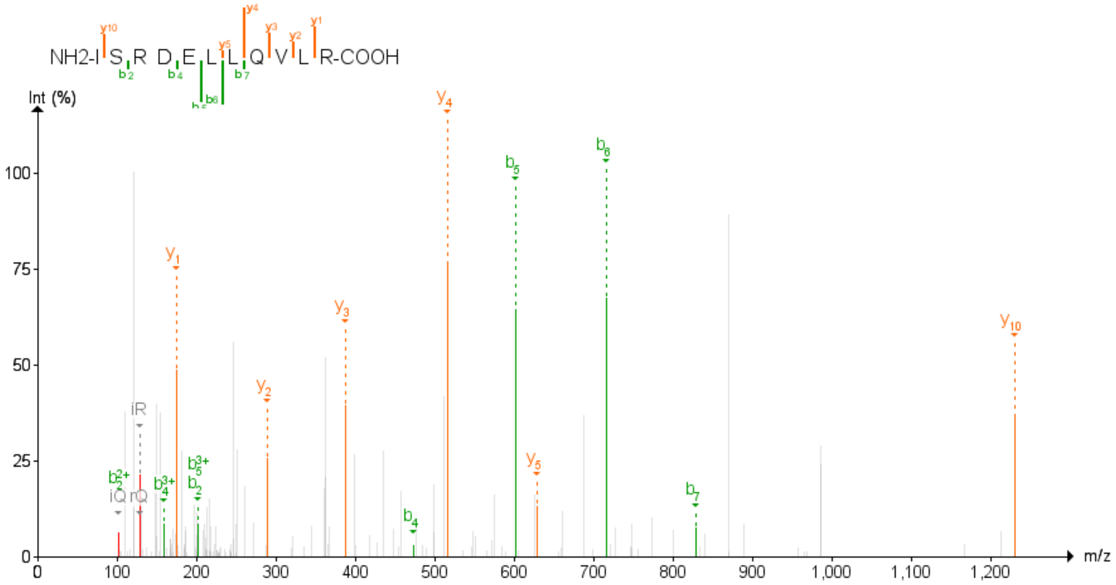

Figure 20: CHP1 Second experiment

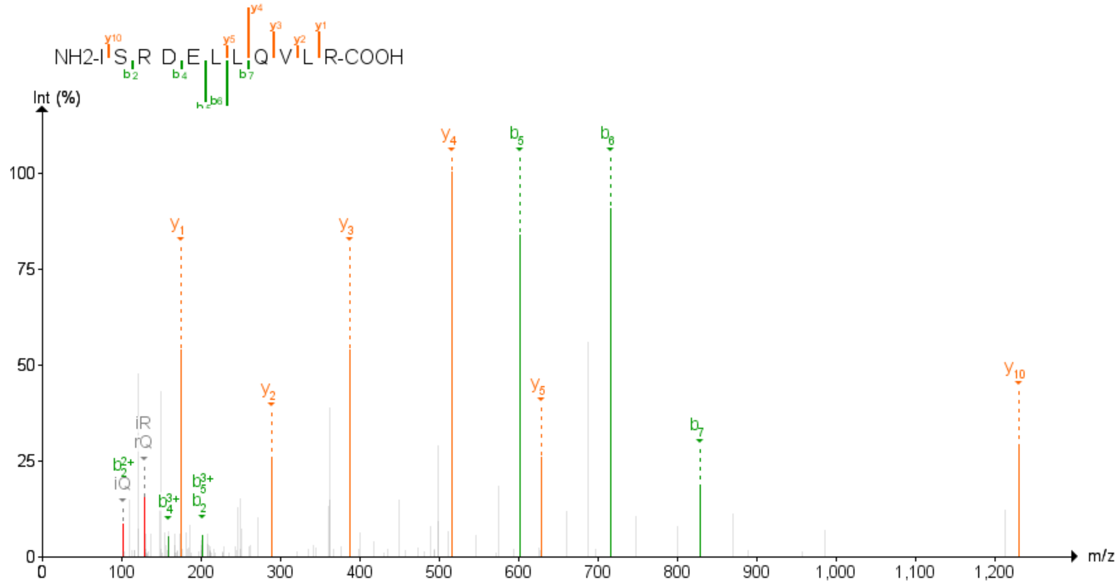

Figure 21: CHP1 Third experiment

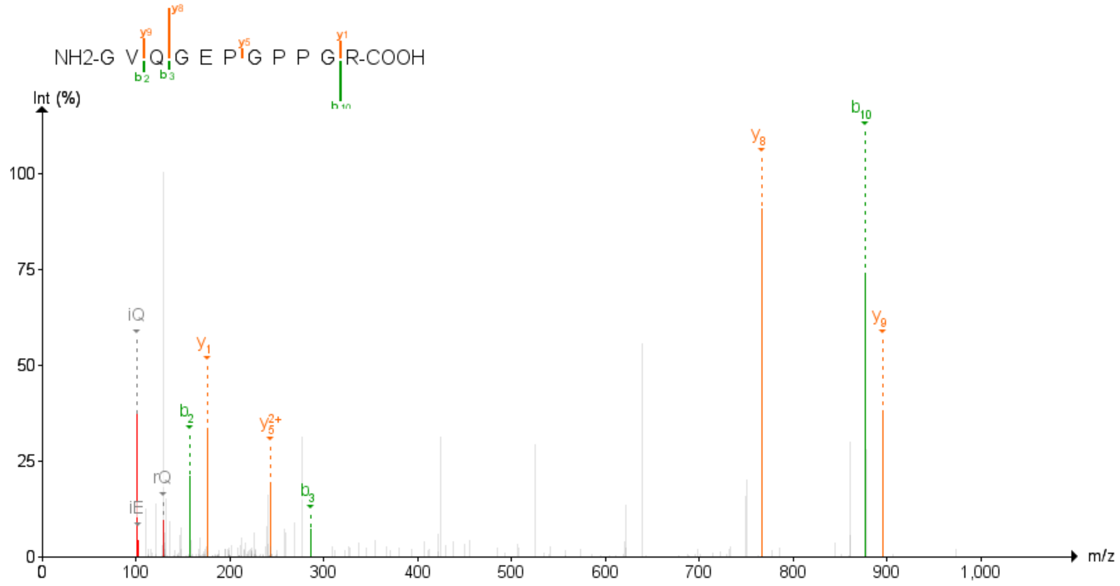

Figure 22: COL16A1 Second experiment

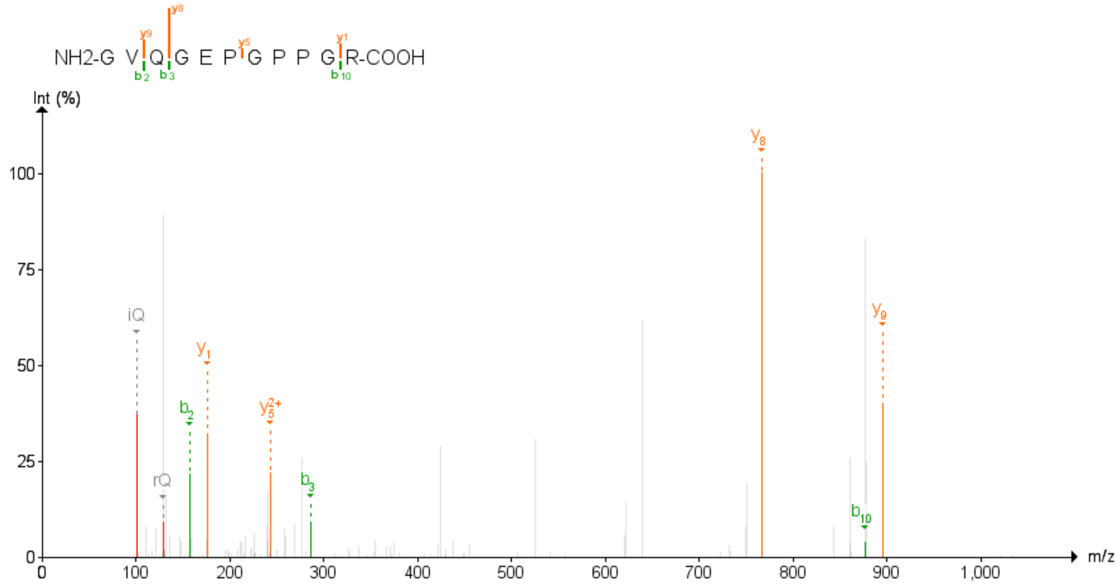

Figure 23: COL16A1 Third experiment

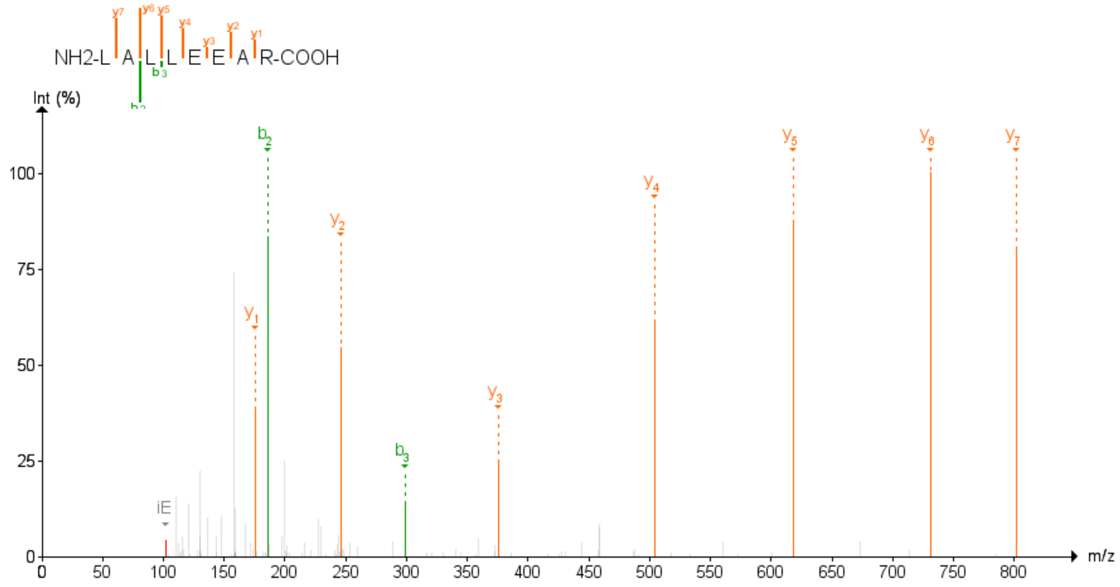

Figure 24: CROCC First experiment

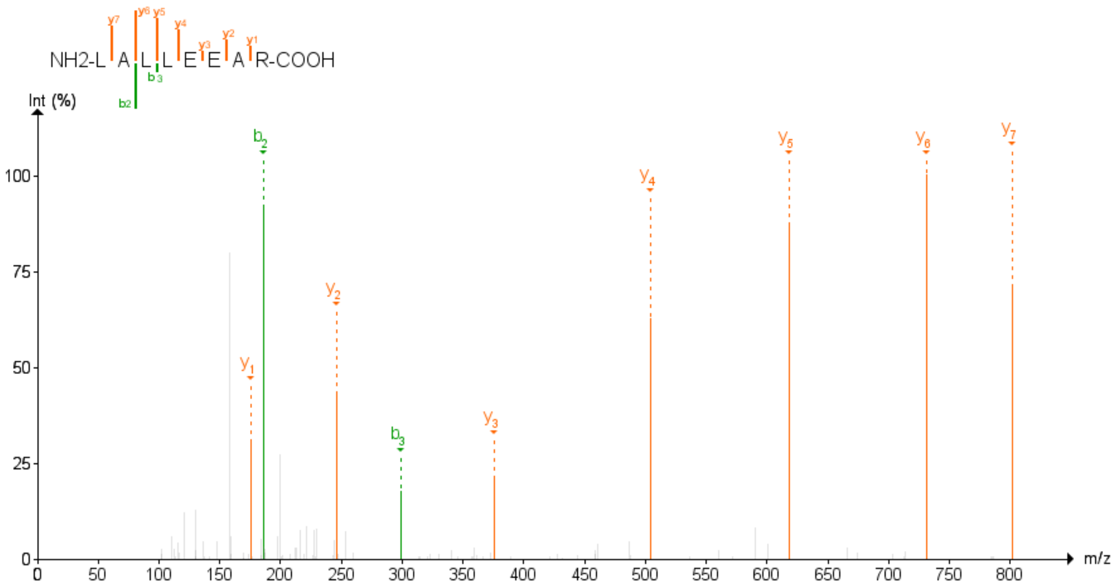

Figure 25: CROCC Third experiment

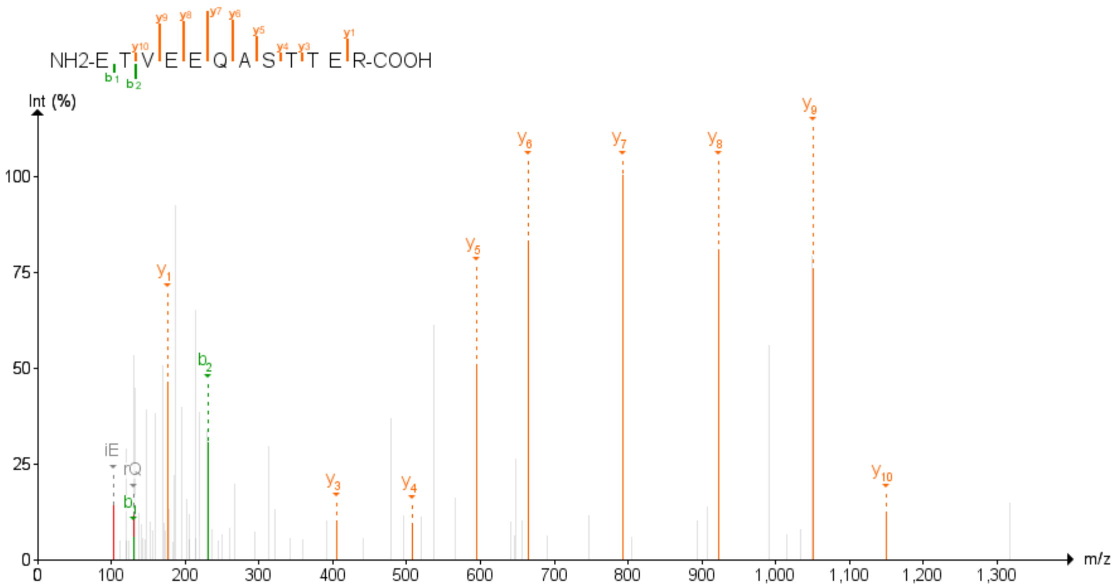

Figure 26: CUL4B First experiment

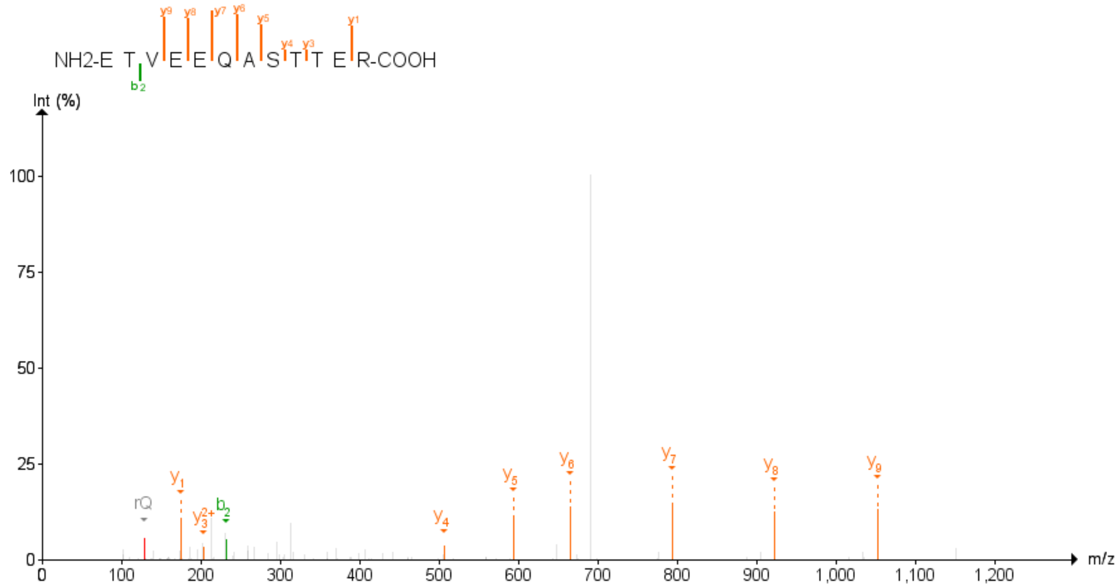

Figure 27: CUL4B Third experiment

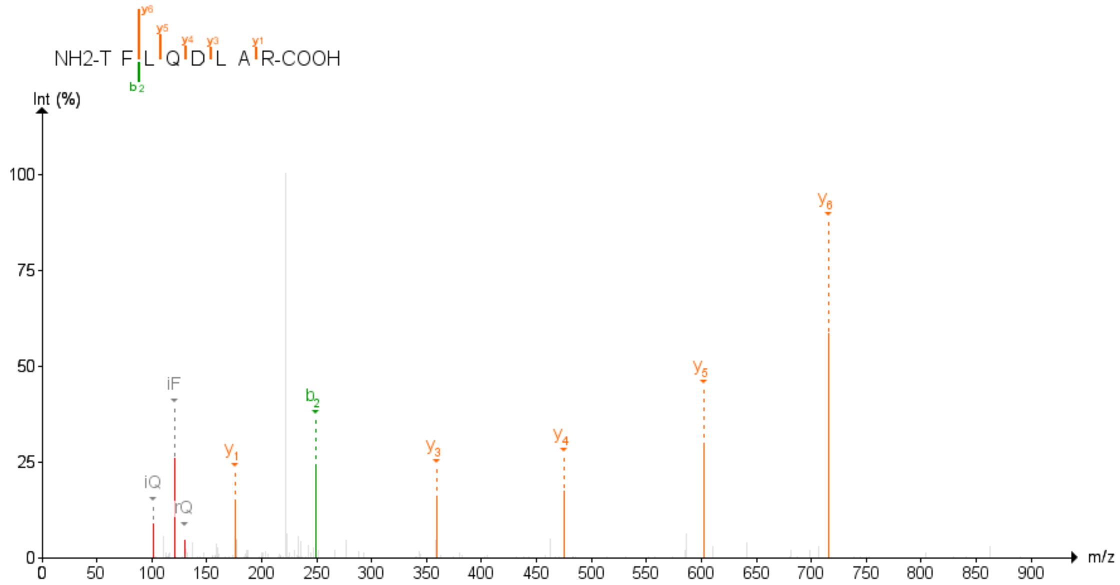

Figure 28: EI24 Second experiment

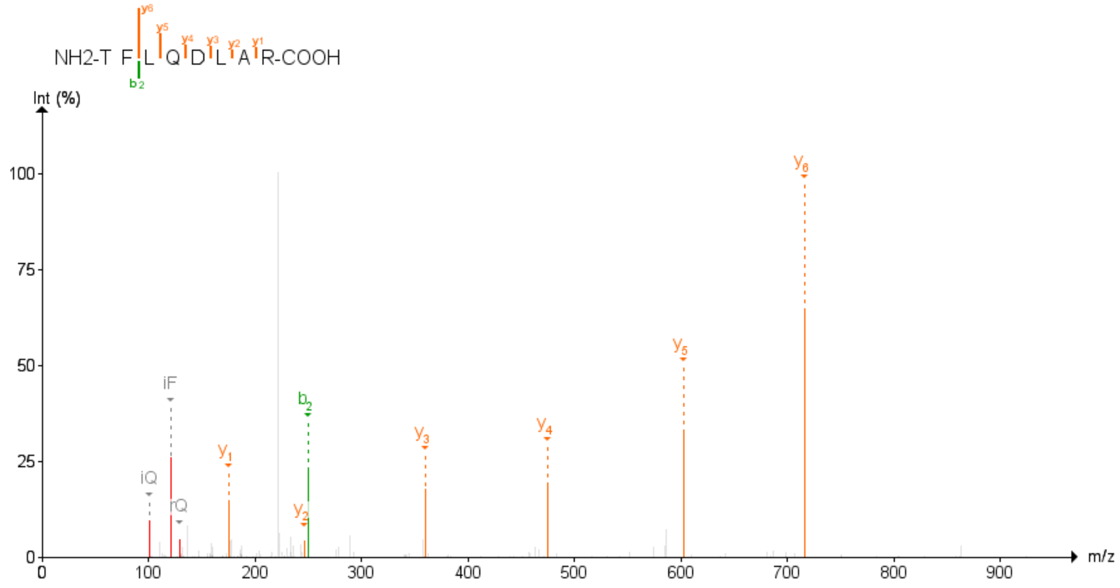

Figure 29: EI24 Third experiment

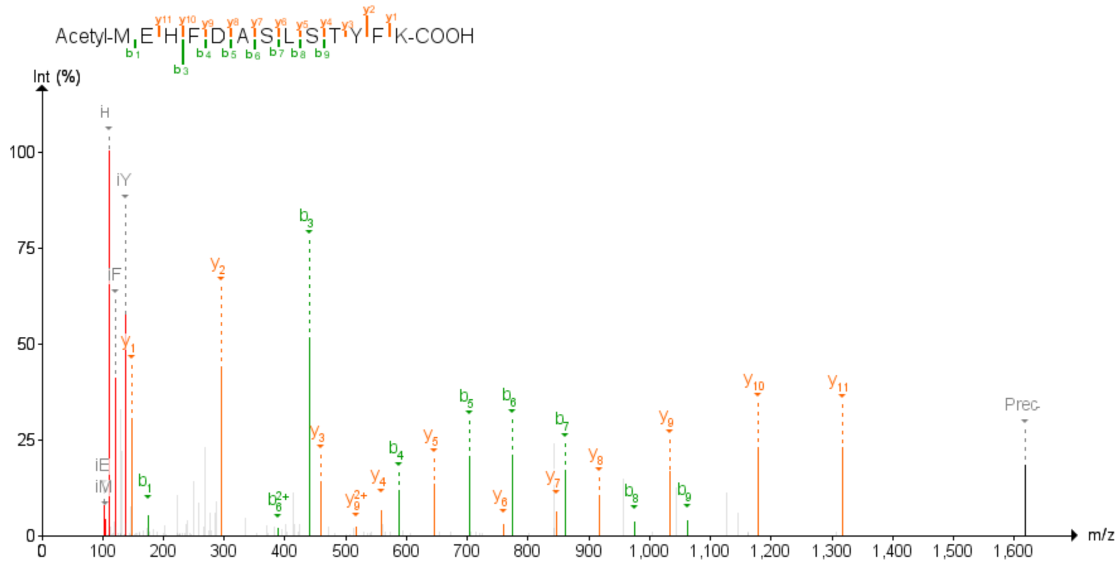

Figure 30: ELOVL5 First experiment

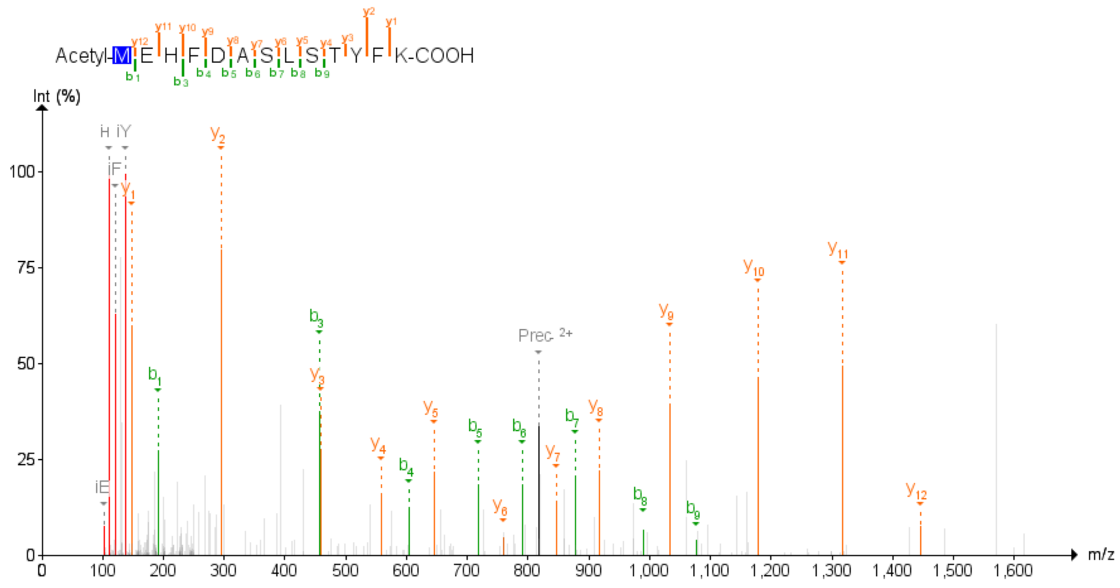

Figure 31: ELOVL5 Second experiment

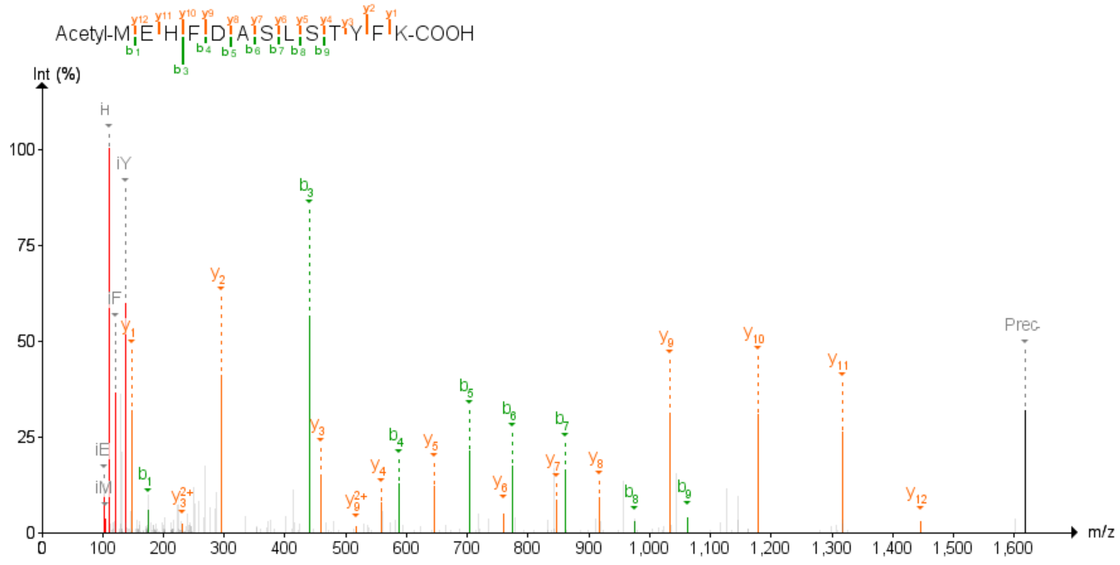

Figure 32: ELOVL5 Third experiment

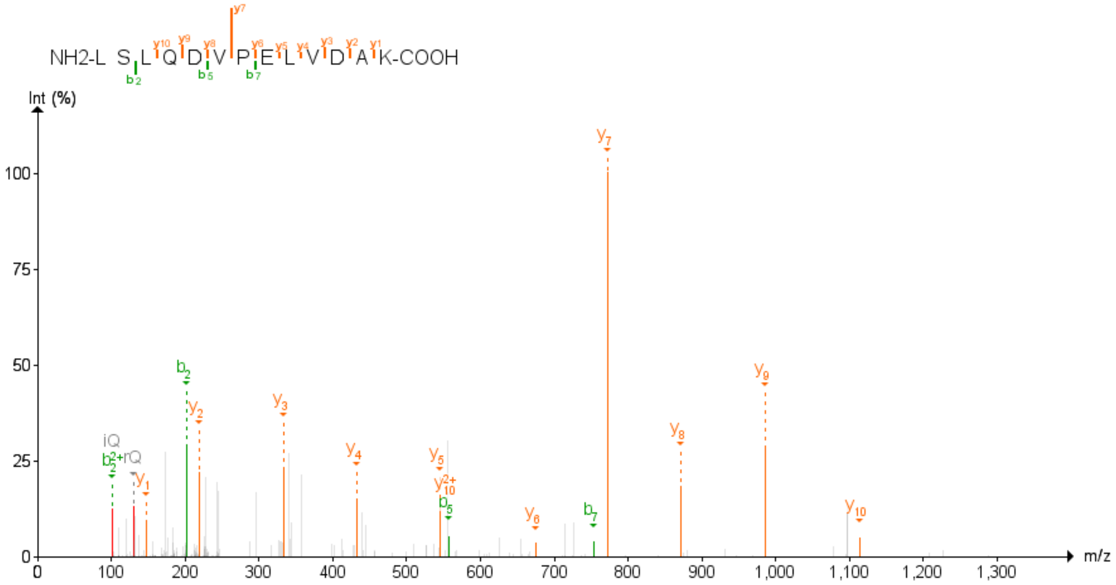

Figure 33: FAM101B First experiment

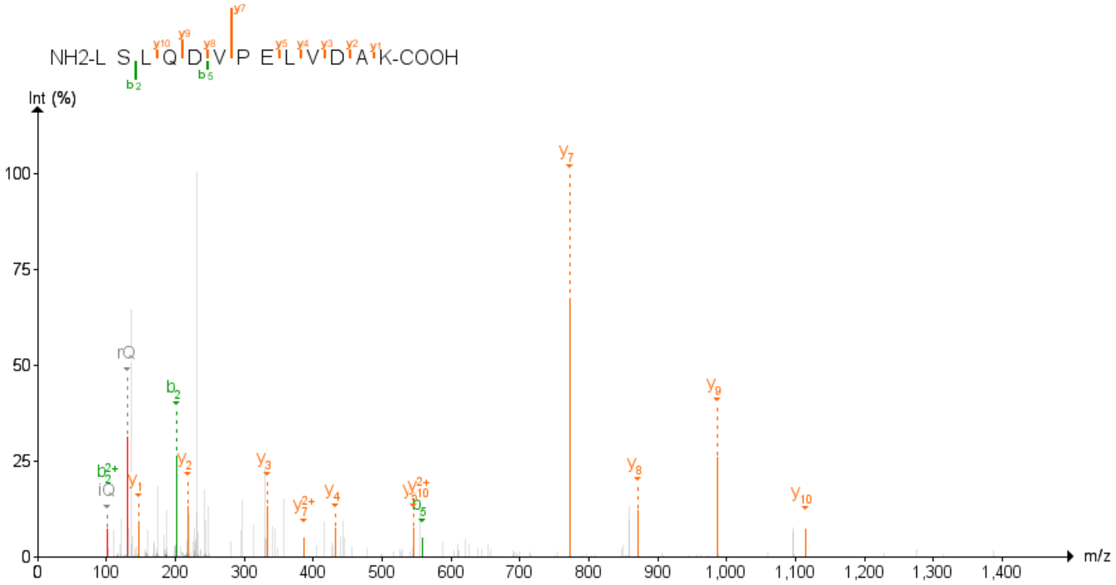

Figure 34: FAM101B Second experiment

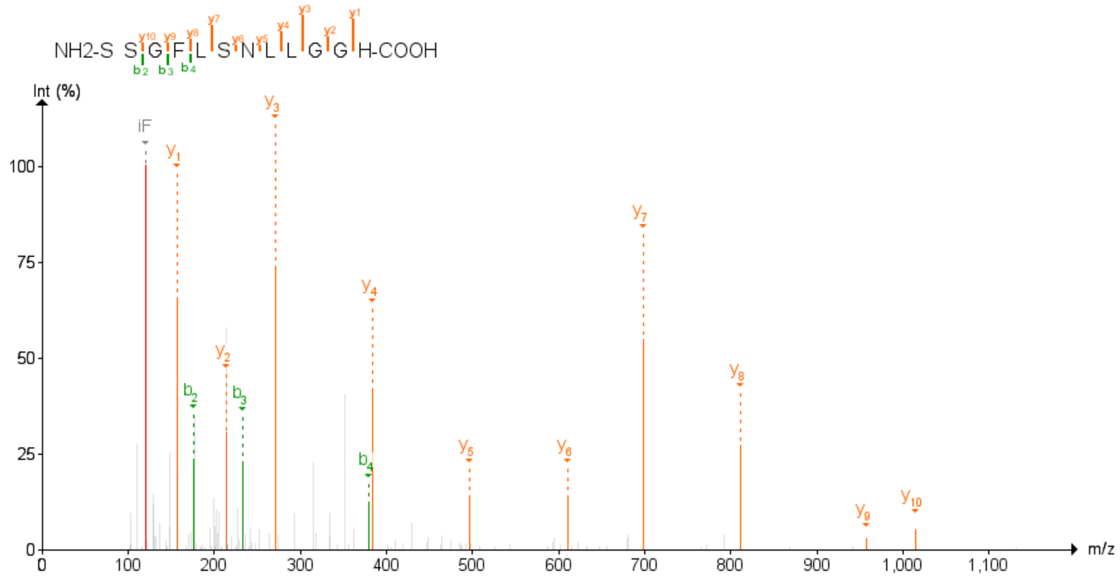

Figure 35: FAM134B First experiment

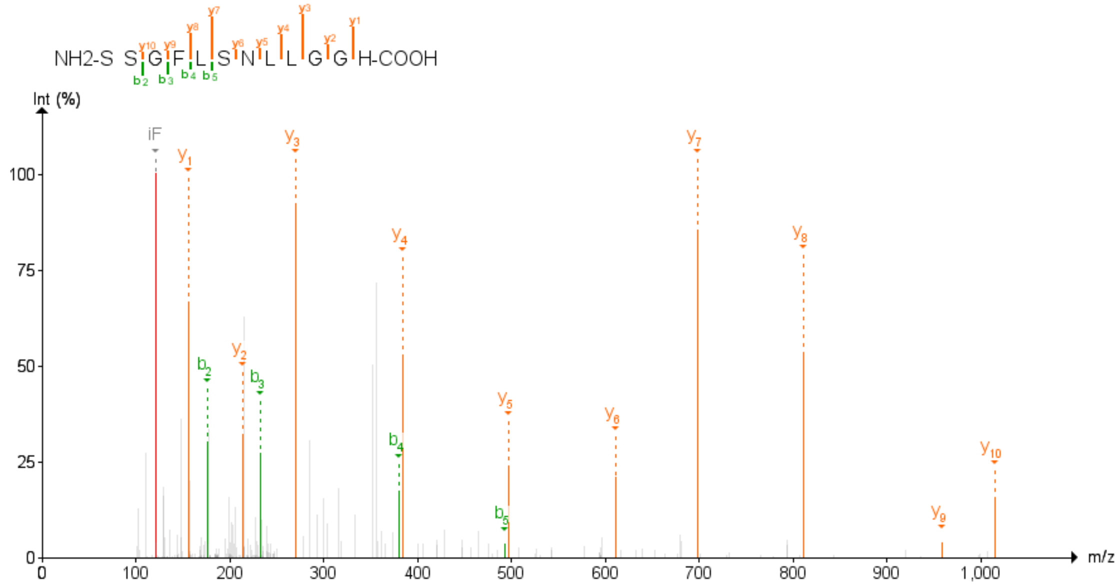

Figure 36: FAM134B Second experiment

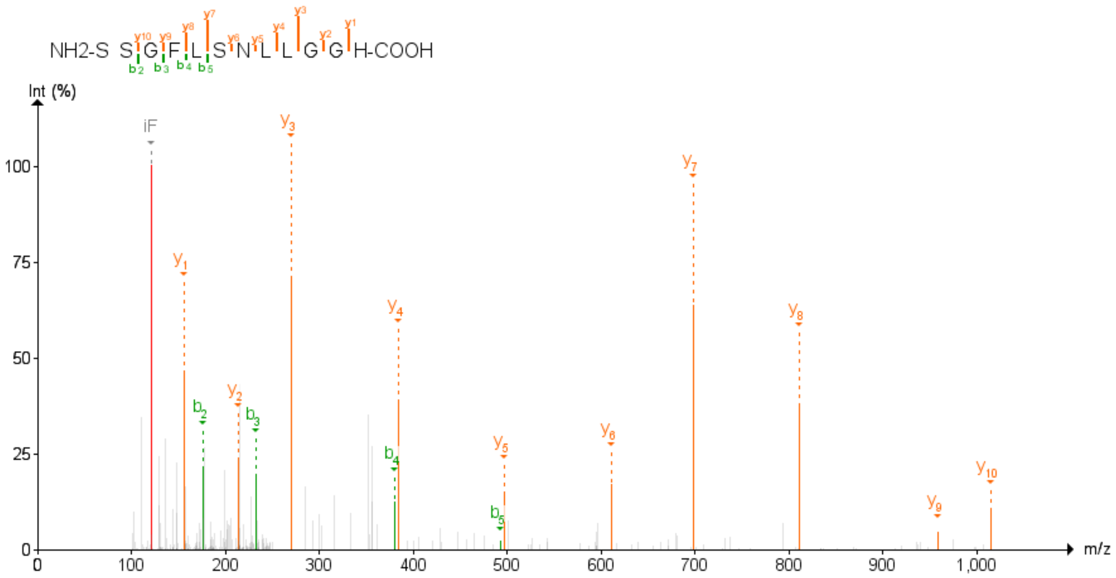

Figure 37: FAM134B Third experiment

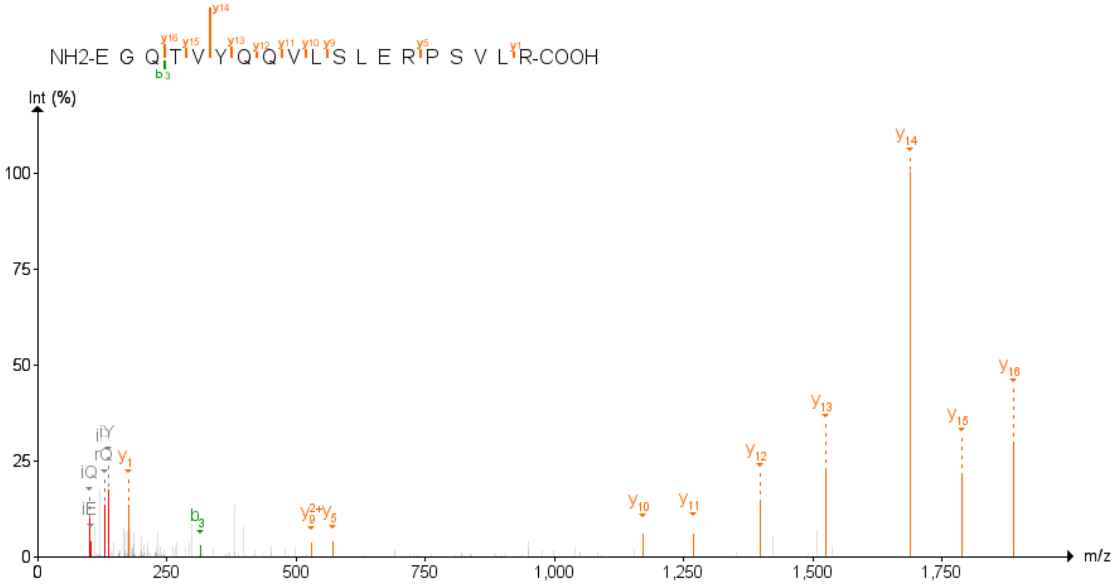

Figure 38: GBA2 First experiment

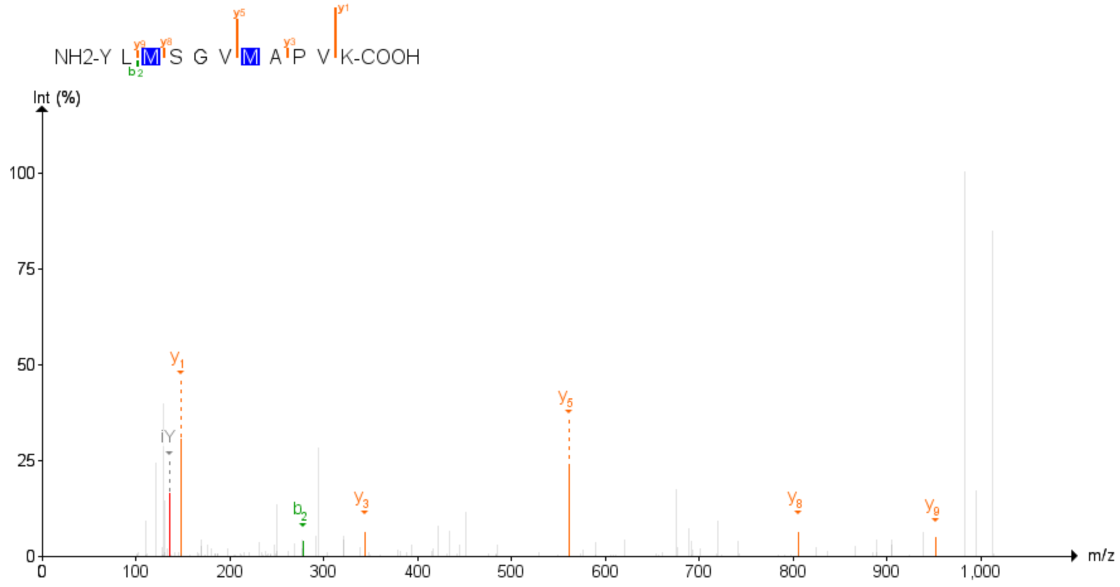

Figure 39: GBA2 Second experiment

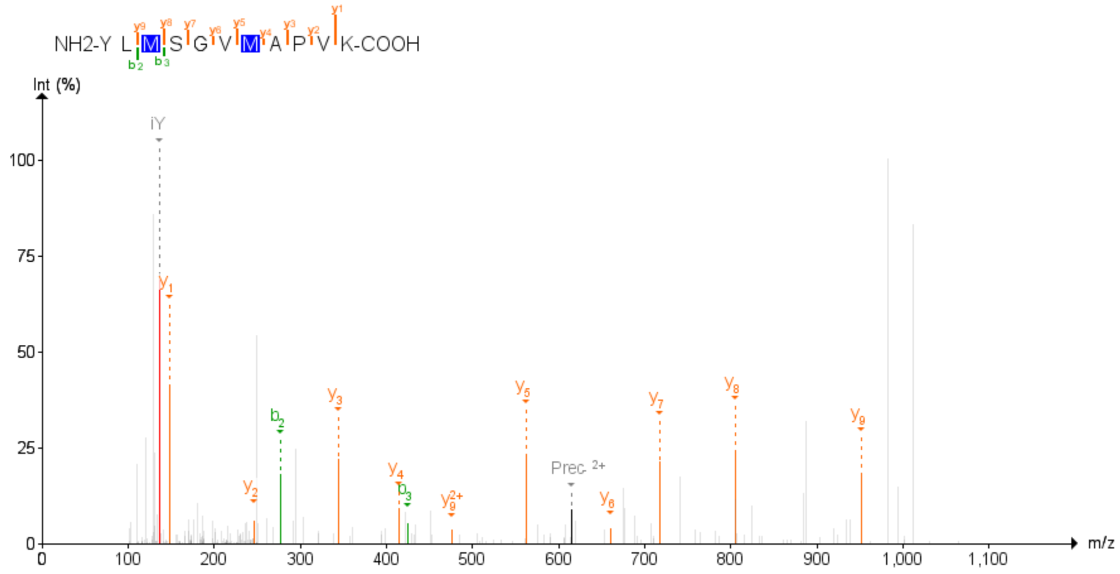

Figure 40: GBA2 Third experiment

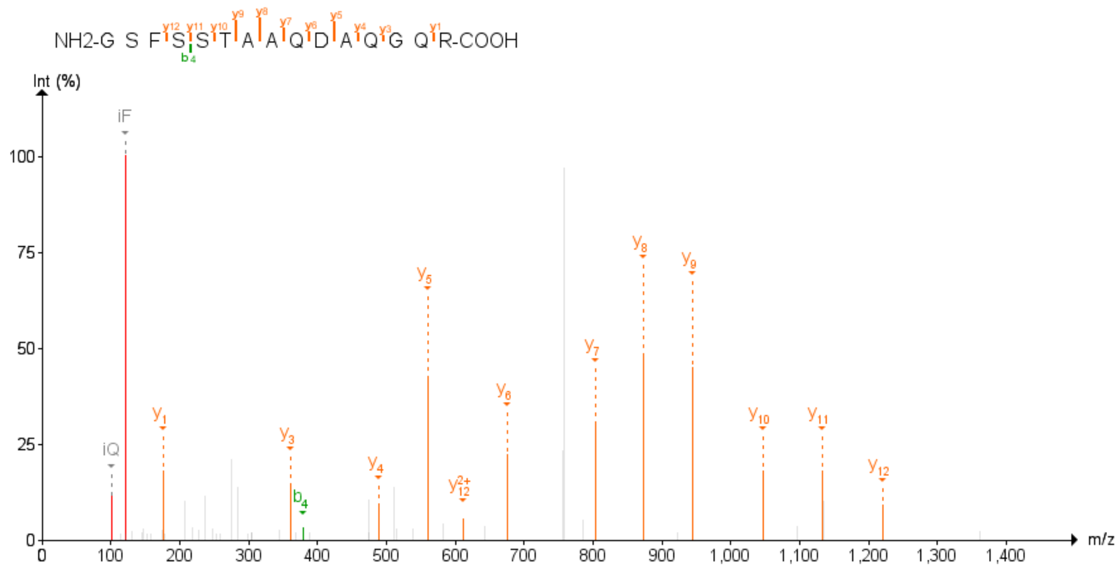

Figure 41: GPR180 First experiment

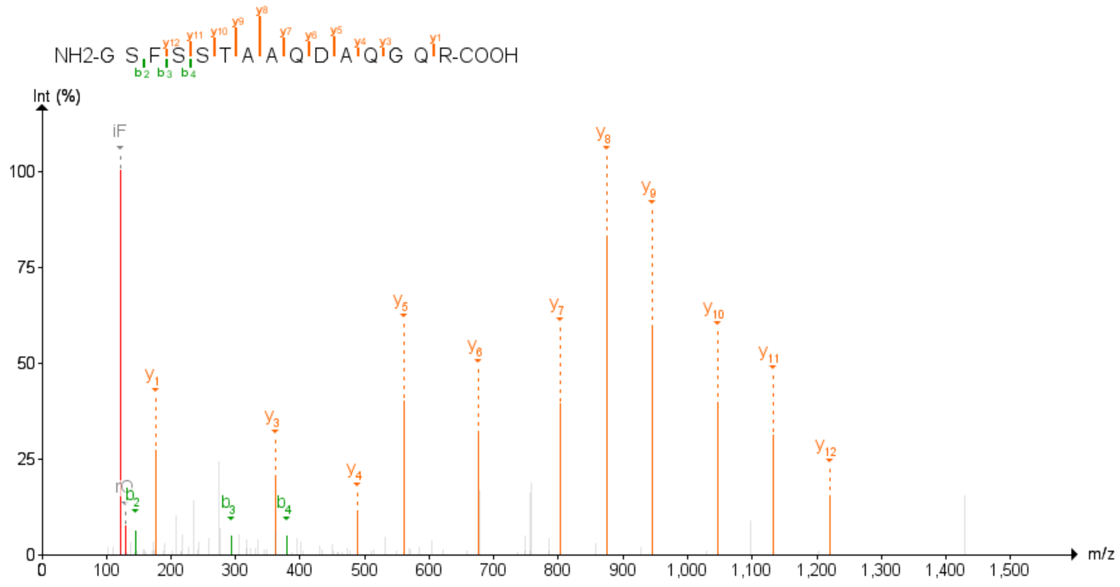

Figure 42: GPR180 Second experiment

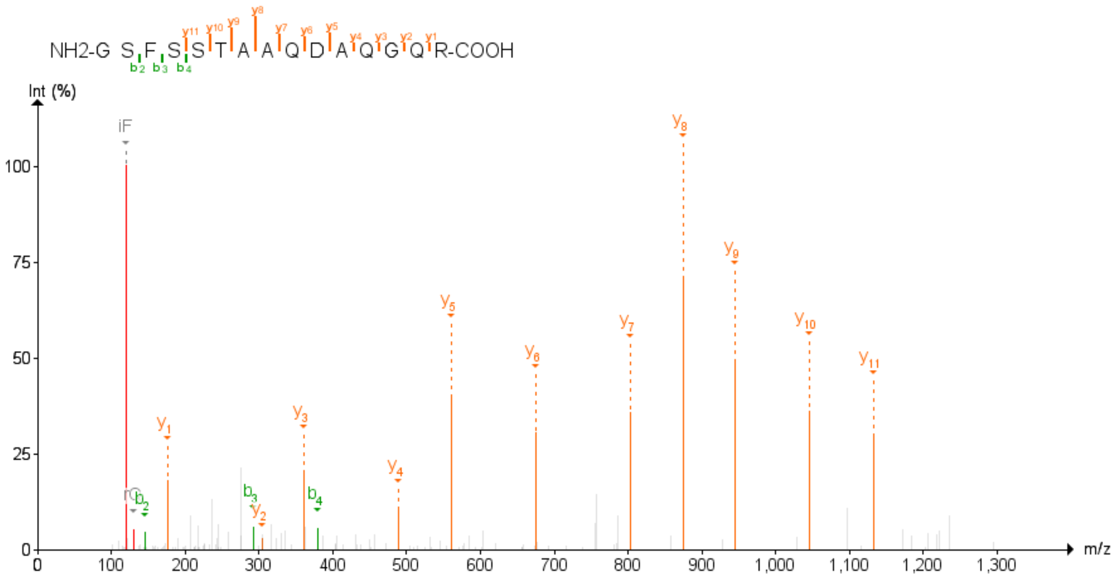

Figure 43: GPR180 Third experiment

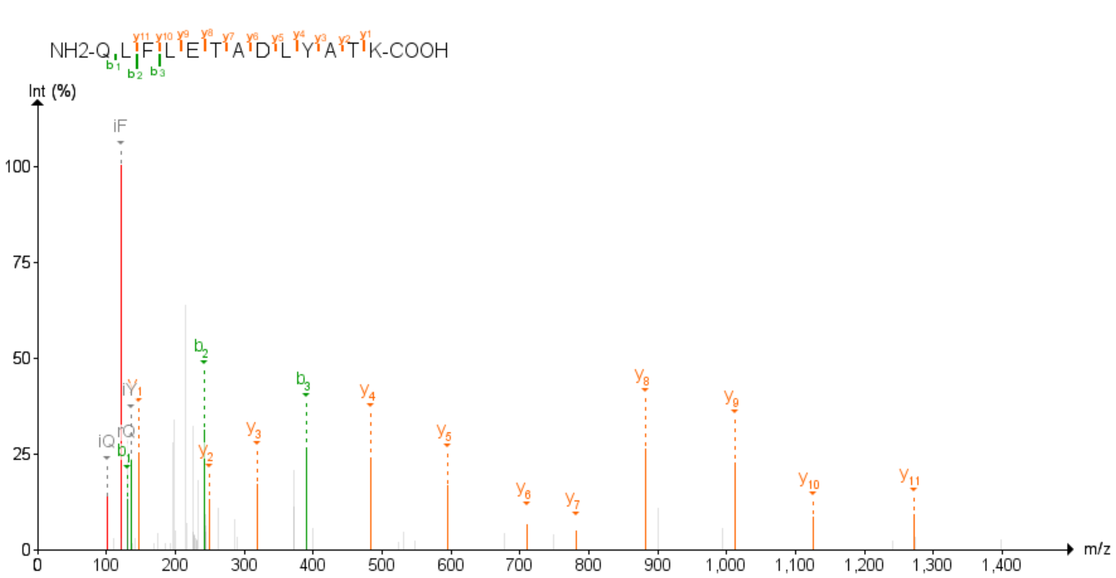

Figure 44: GPR89B;GPR89A First experiment

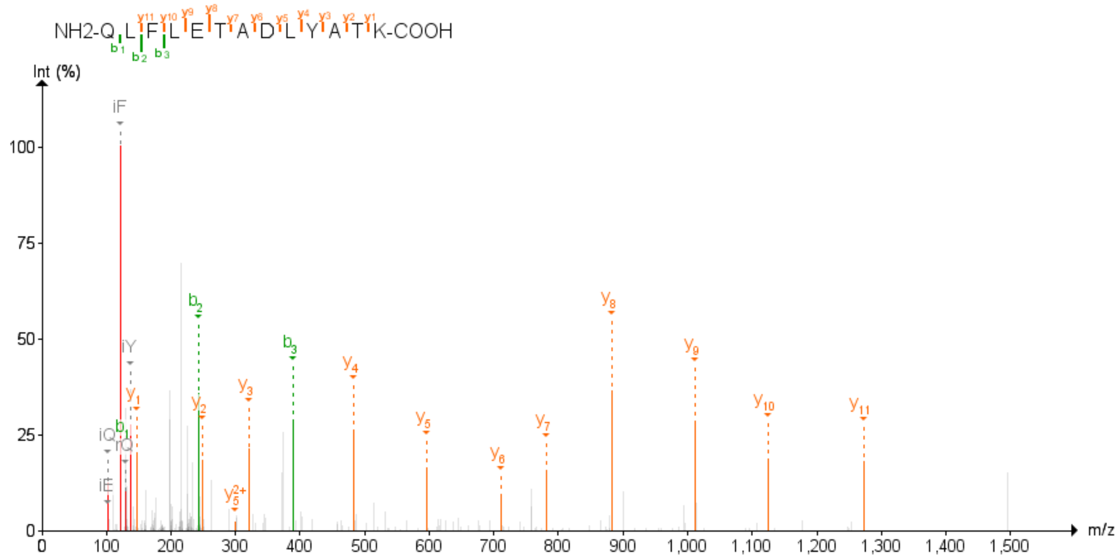

Figure 45: GPR89B;GPR89A Second experiment

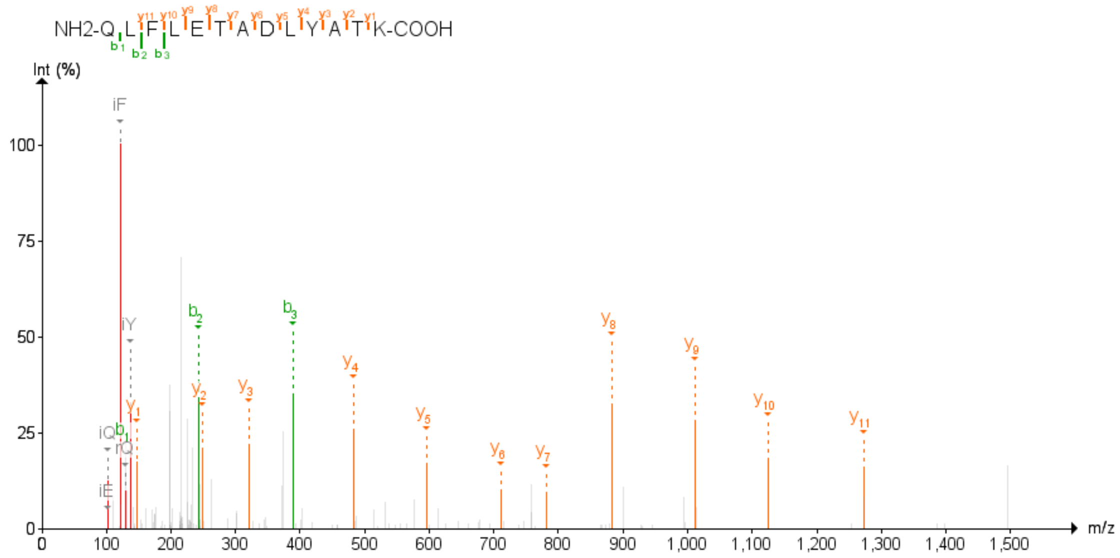

Figure 46: GPR89B;GPR89A Third experiment

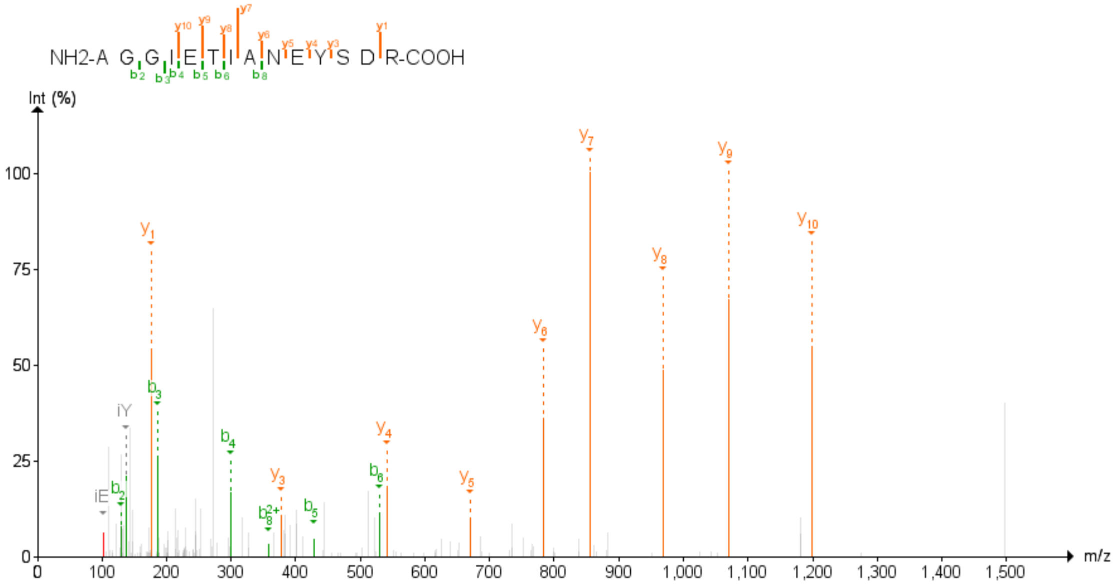

Figure 47: HSPA4 Second experiment

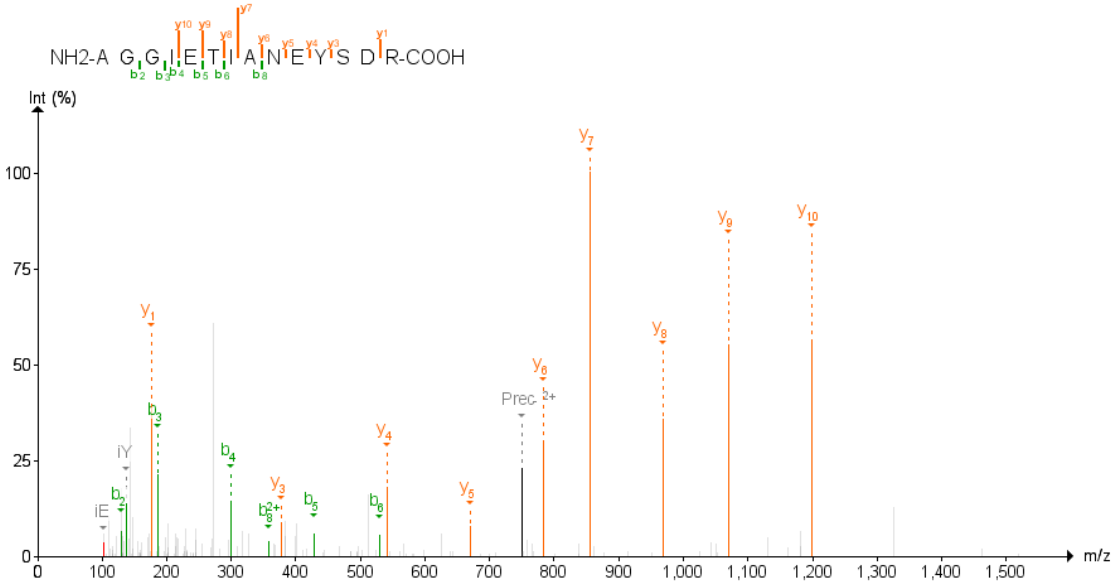

Figure 48: HSPA4 Third experiment

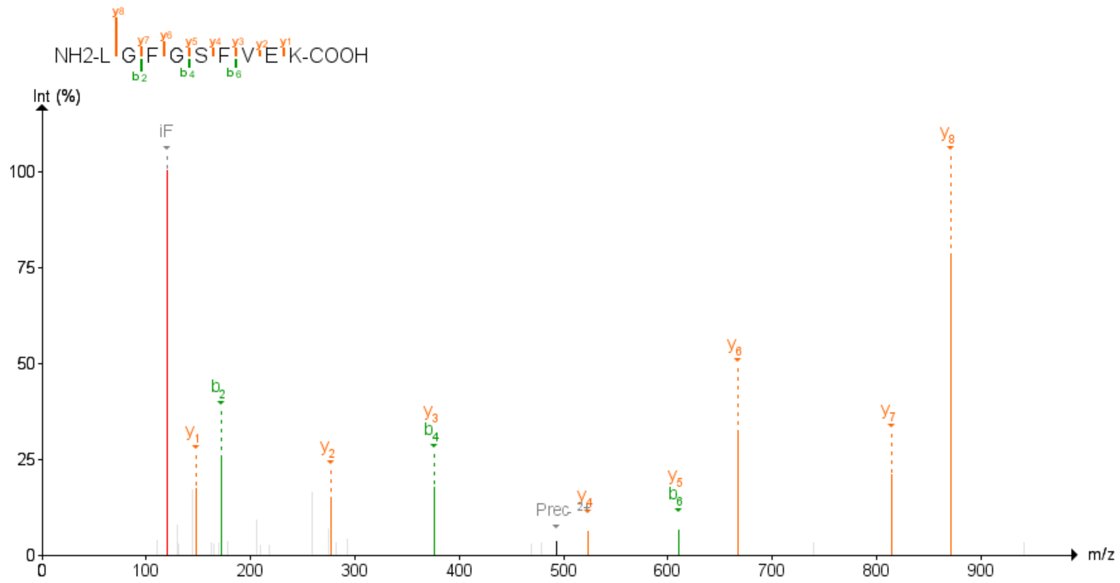

Figure 49: ITGB6 First experiment

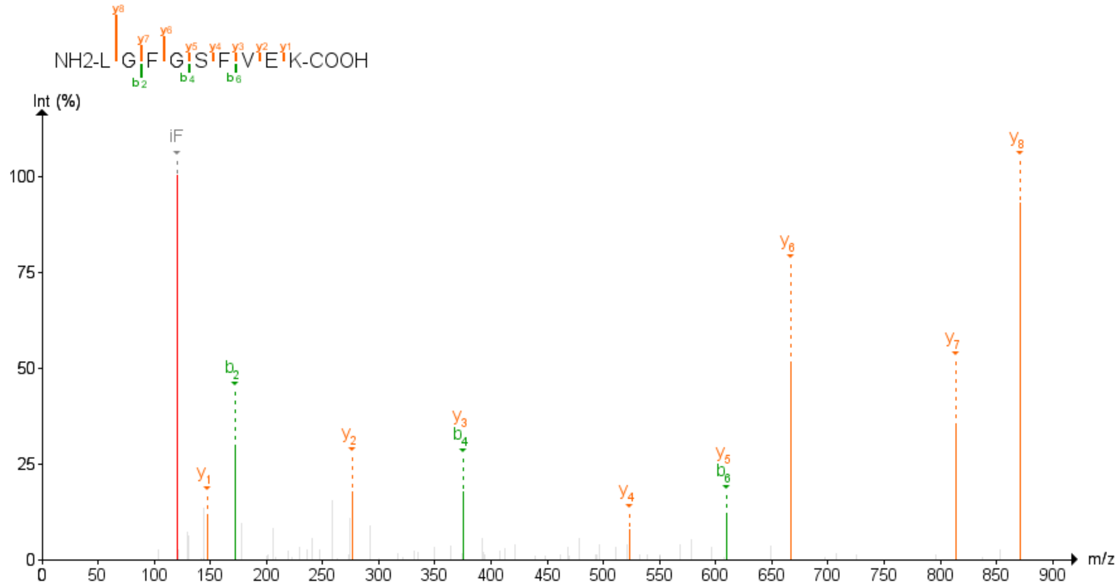

Figure 50: ITGB6 Second experiment

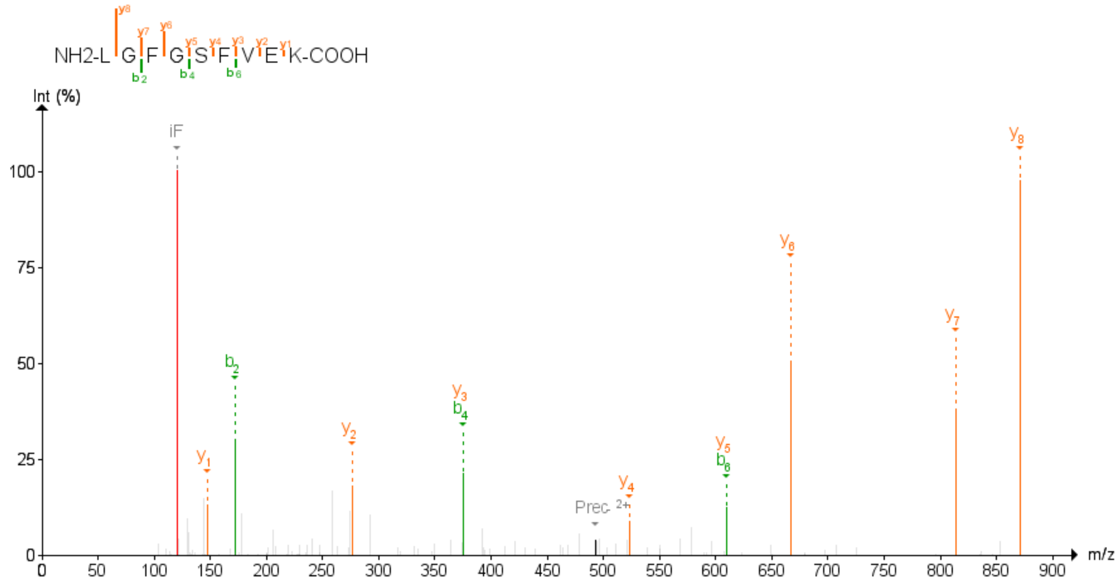

Figure 51: ITGB6 Third experiment

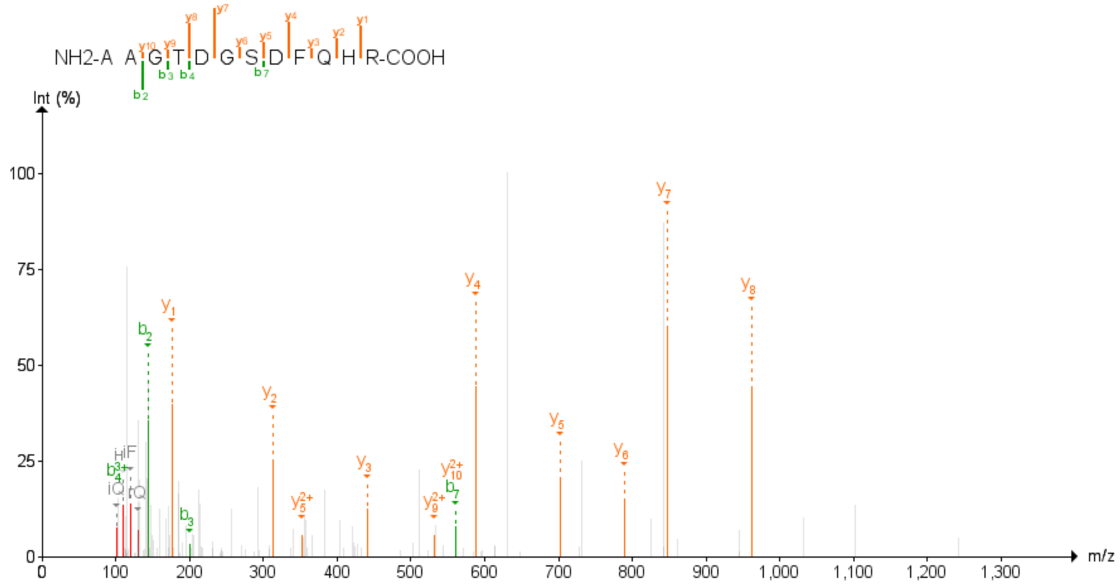

Figure 52: JAGN1 Second experiment

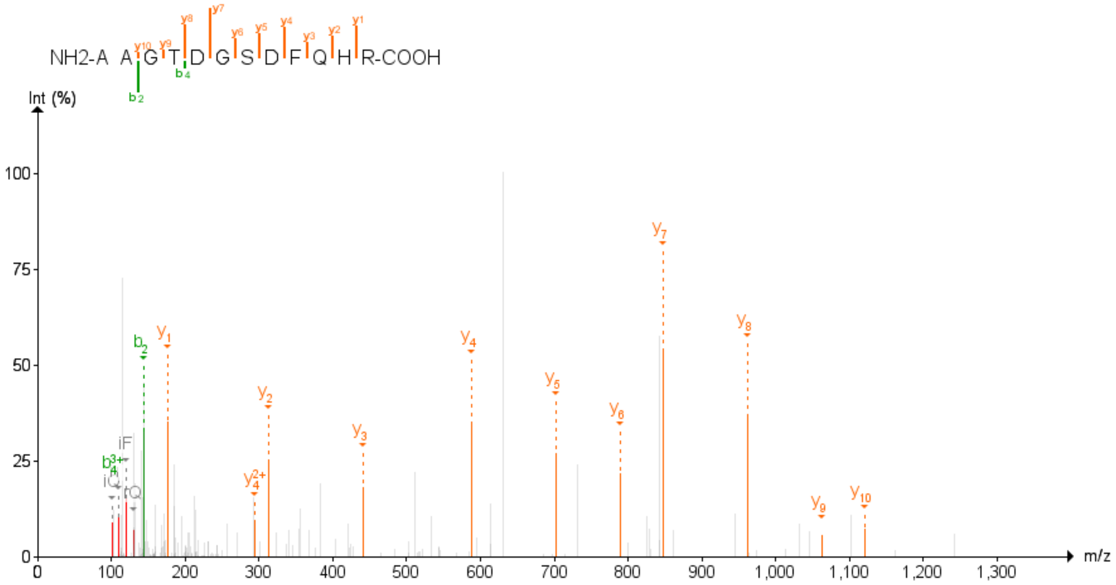

Figure 53: JAGN1 Third experiment

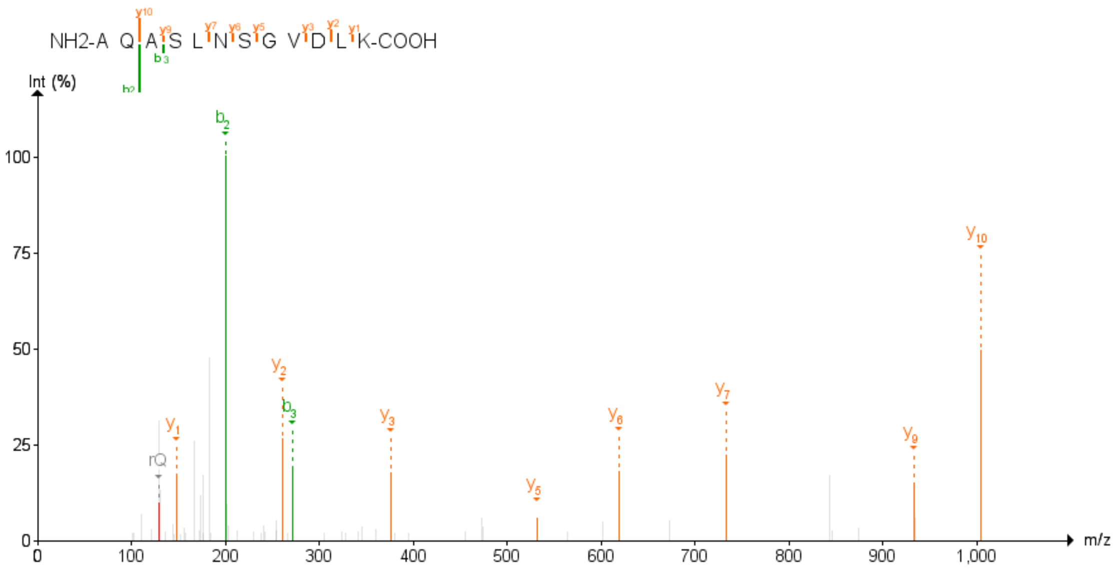

Figure 54: MOB3B First experiment

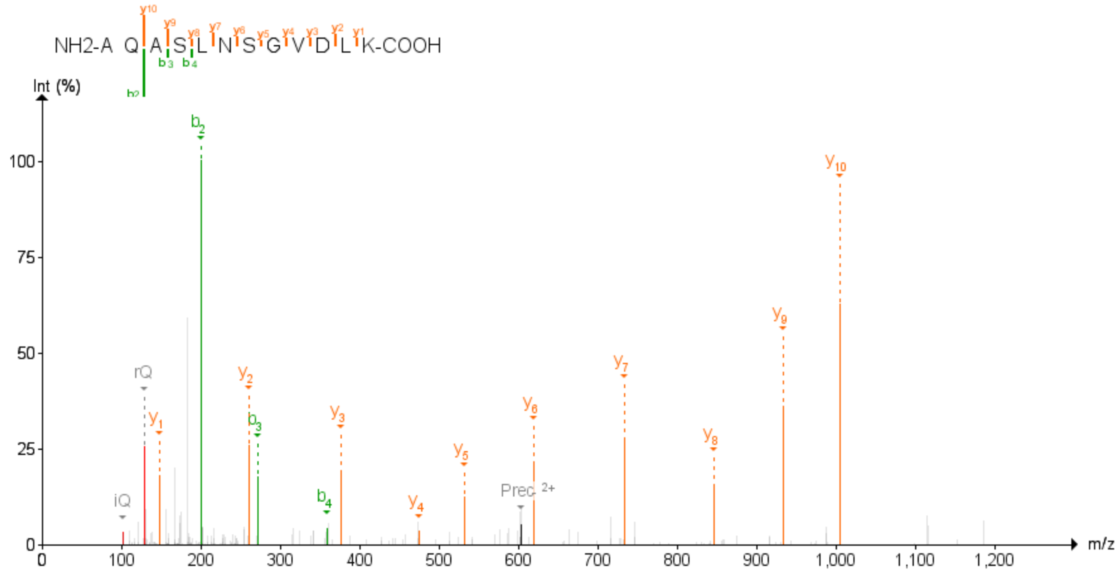

Figure 55: MOB3B Second experiment

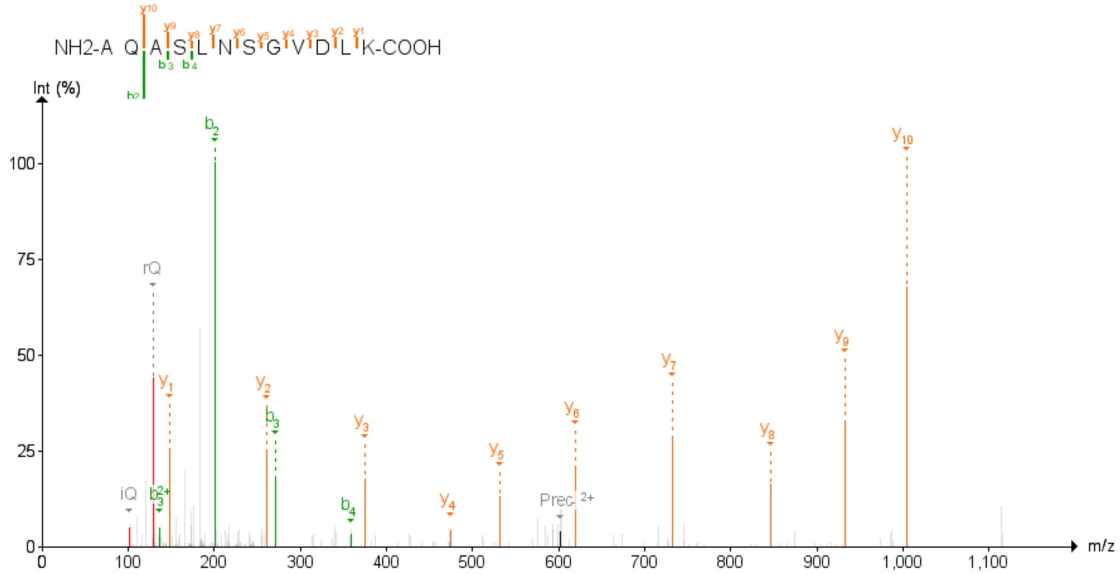

Figure 56: MOB3B Third experiment

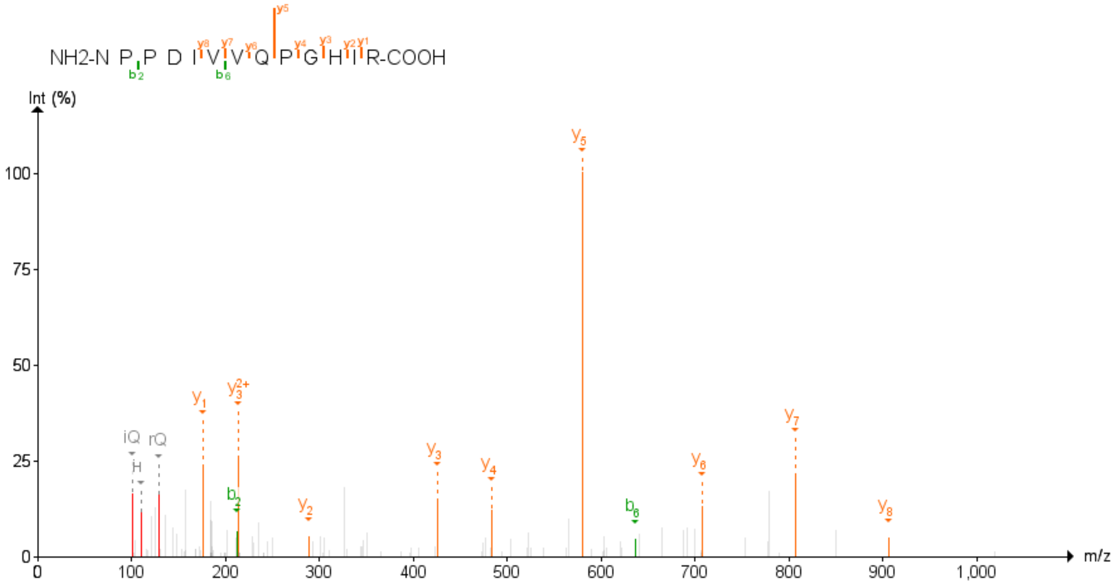

Figure 57: MPZL1 First experiment

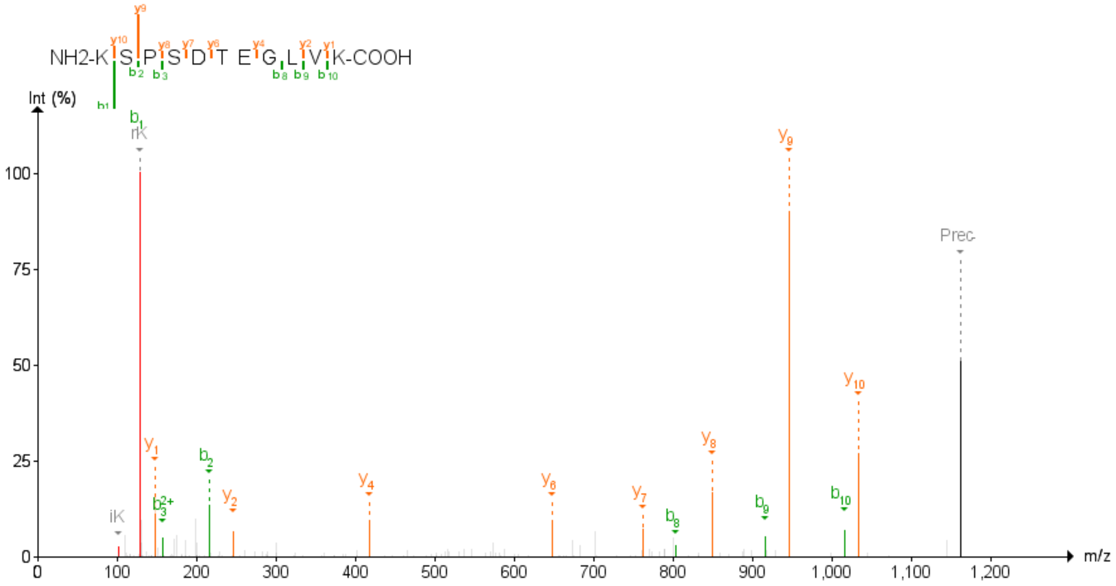

Figure 58: MPZL1 Second experiment

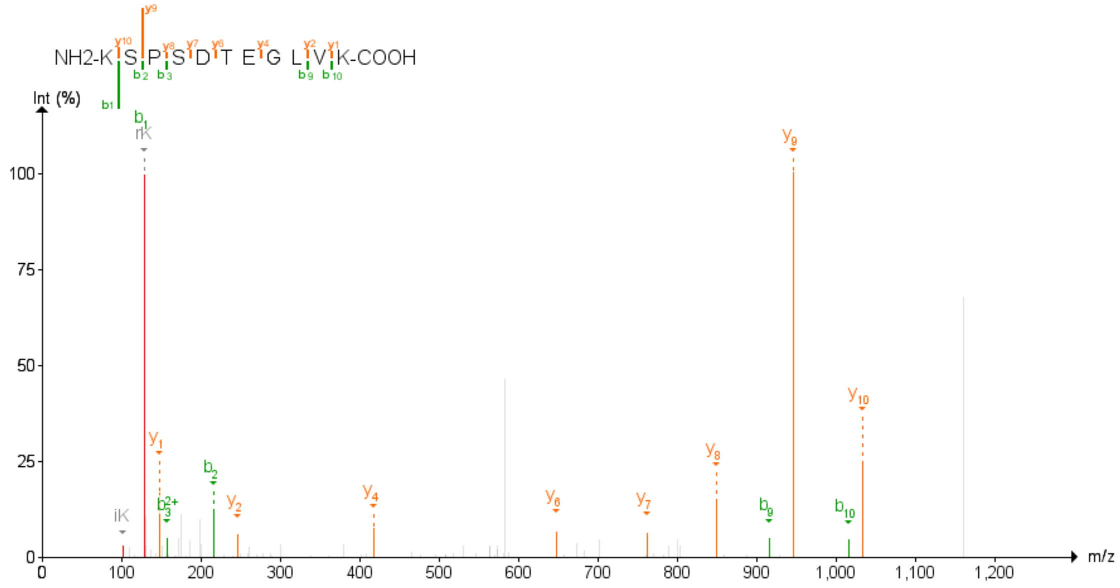

Figure 59: MPZL1 Third experiment

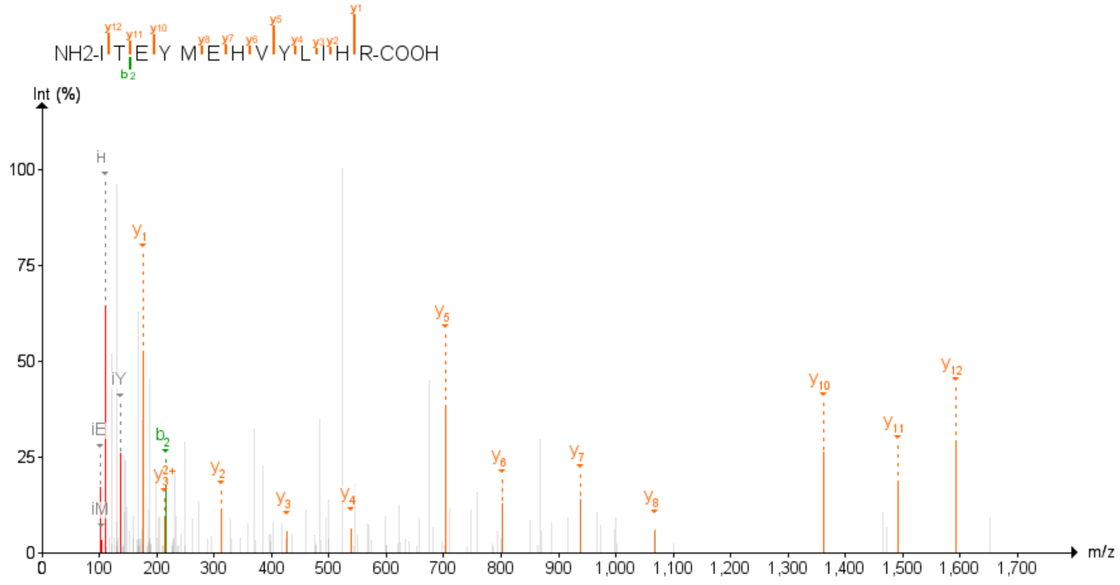

Figure 60: OSBPL2 First experiment

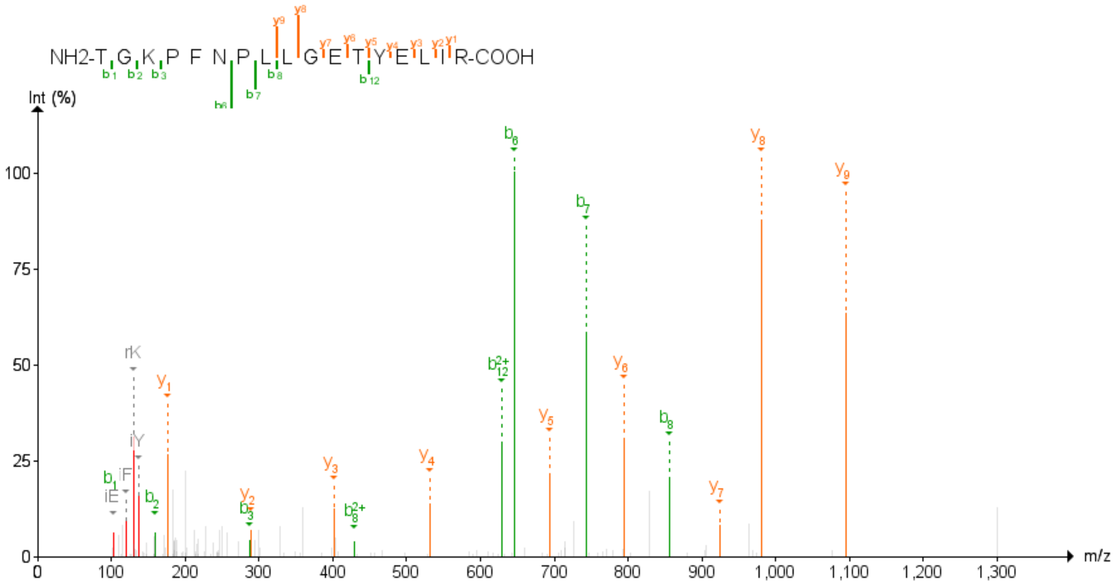

Figure 61: OSBPL2 Second experiment

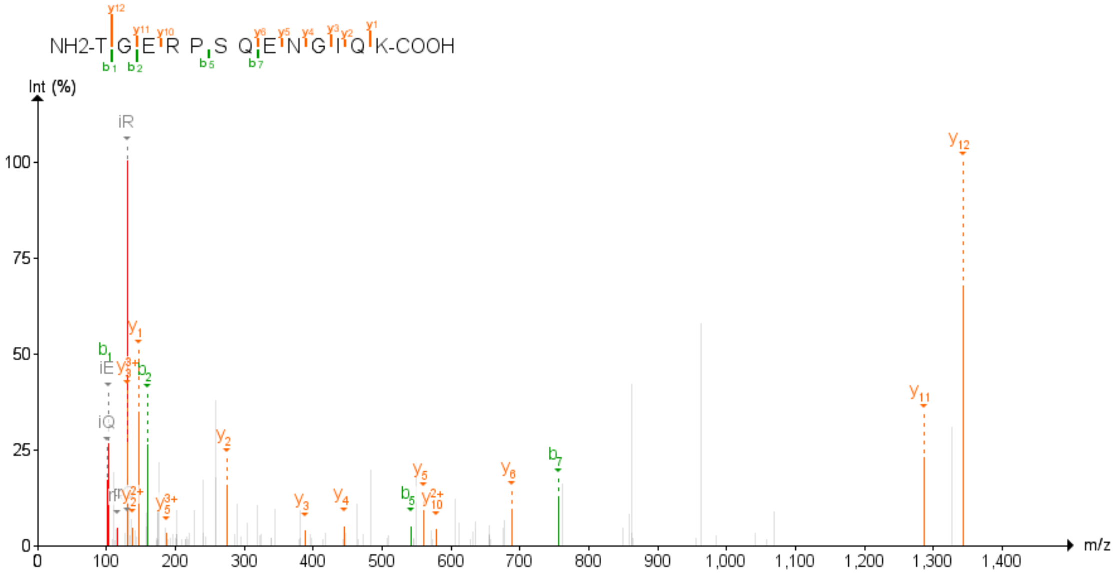

Figure 62: OSBPL2 Third experiment

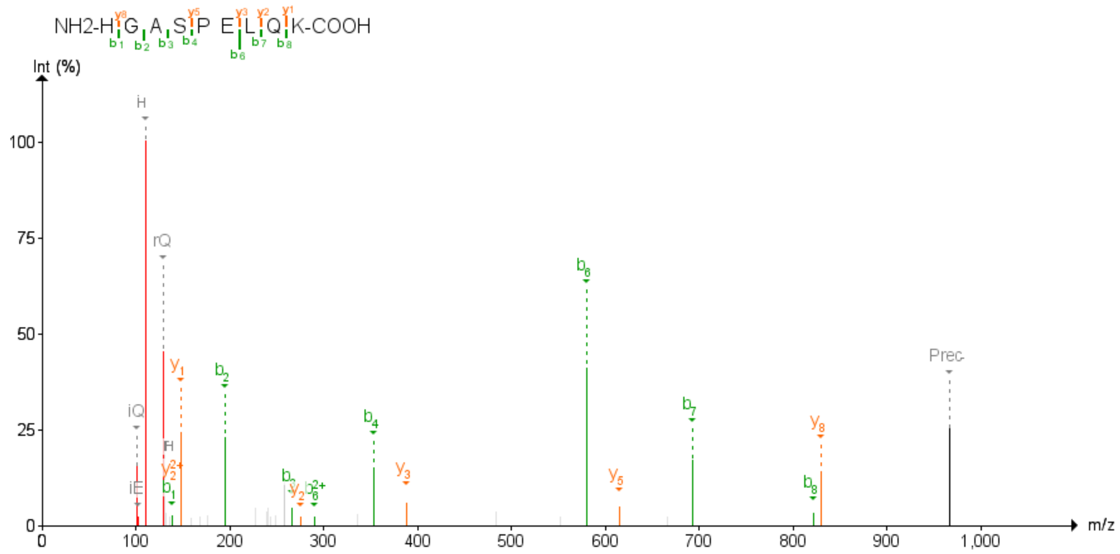

Figure 63: PEX11B First experiment

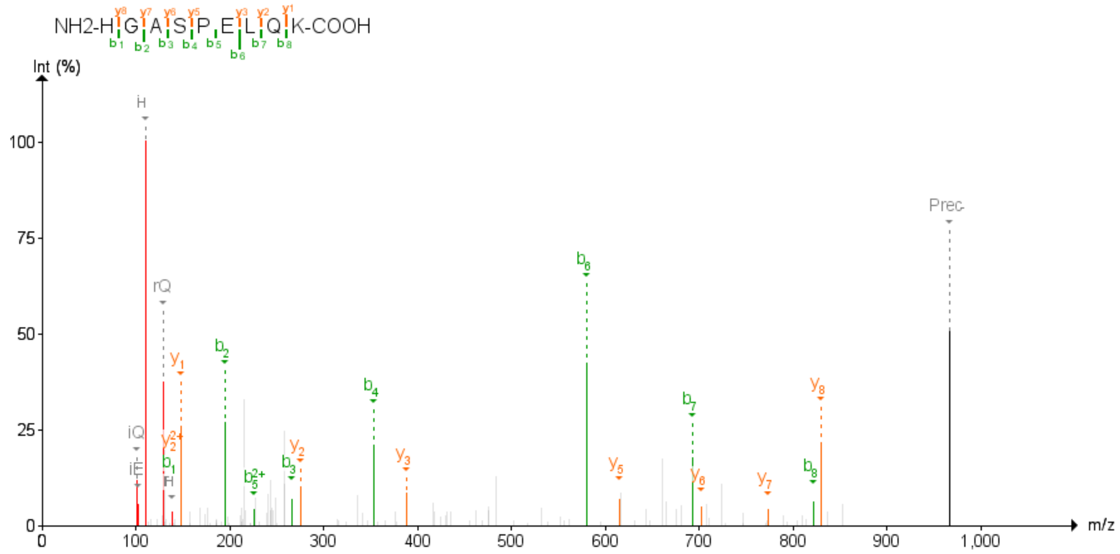

Figure 64: PEX11B Second experiment

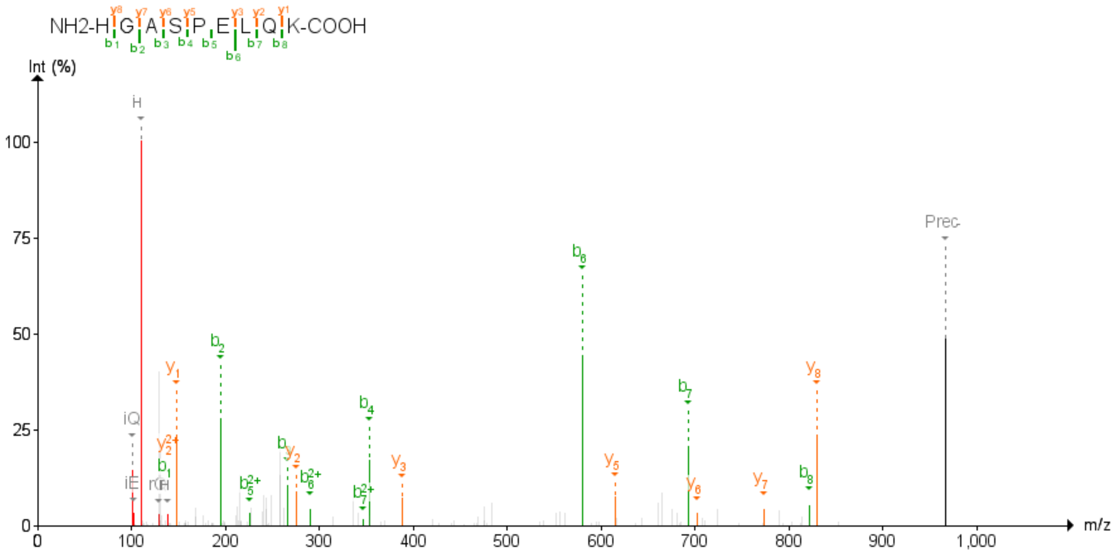

Figure 65: PEX11B Third experiment

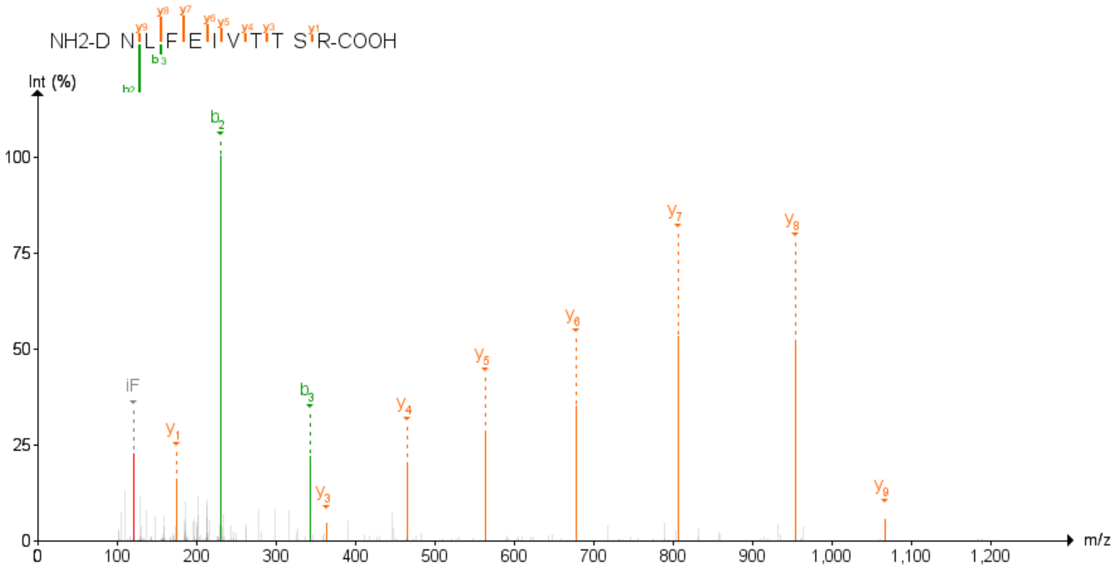

Figure 66: PLEKHA1 First experiment

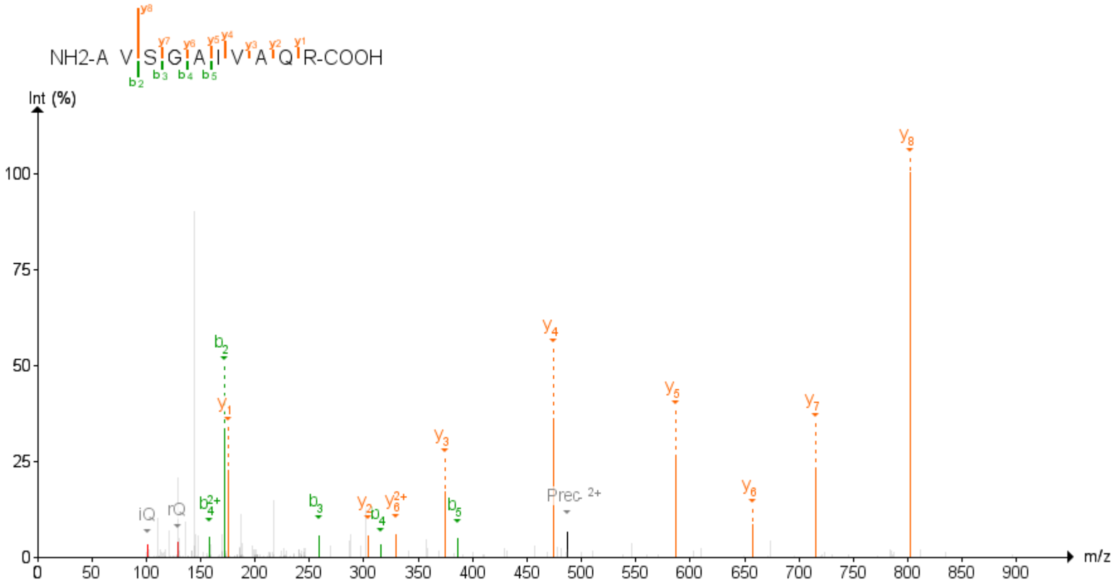

Figure 67: PLEKHA1 Second experiment

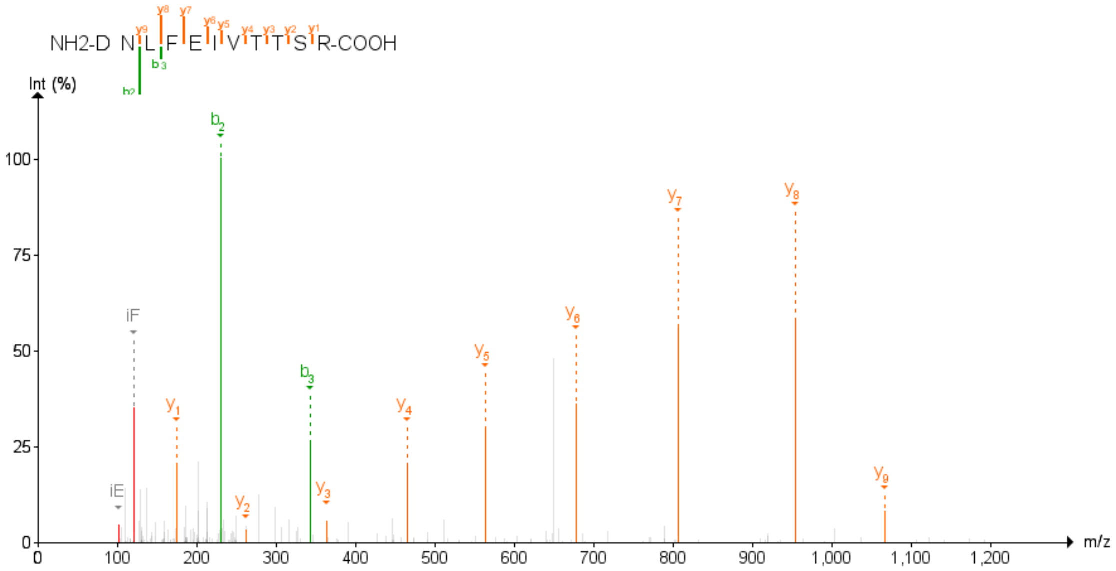

Figure 68: PLEKHA1 Third experiment

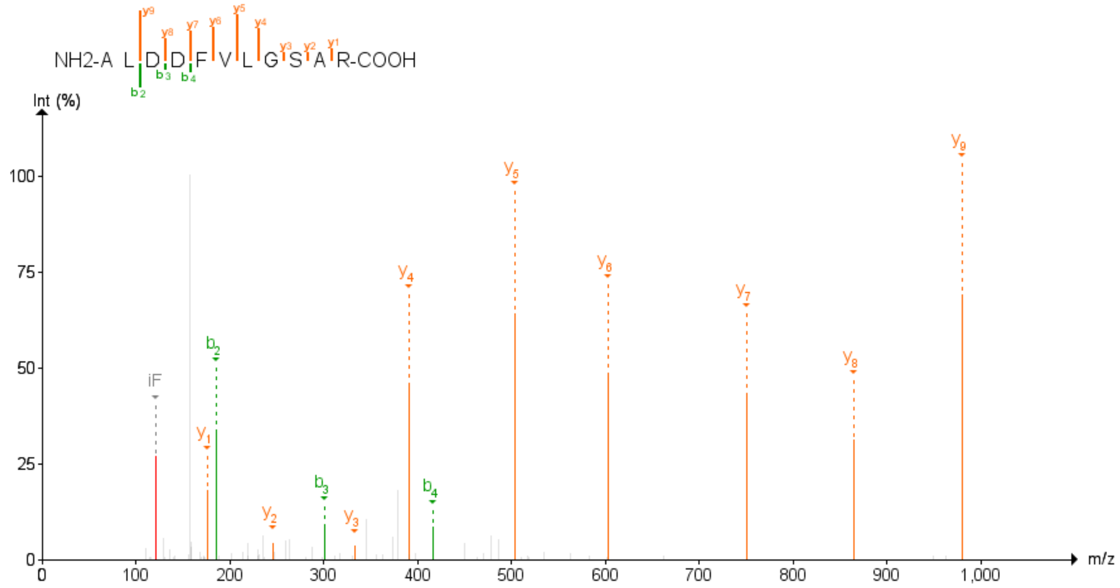

Figure 69: PRAF2 First experiment

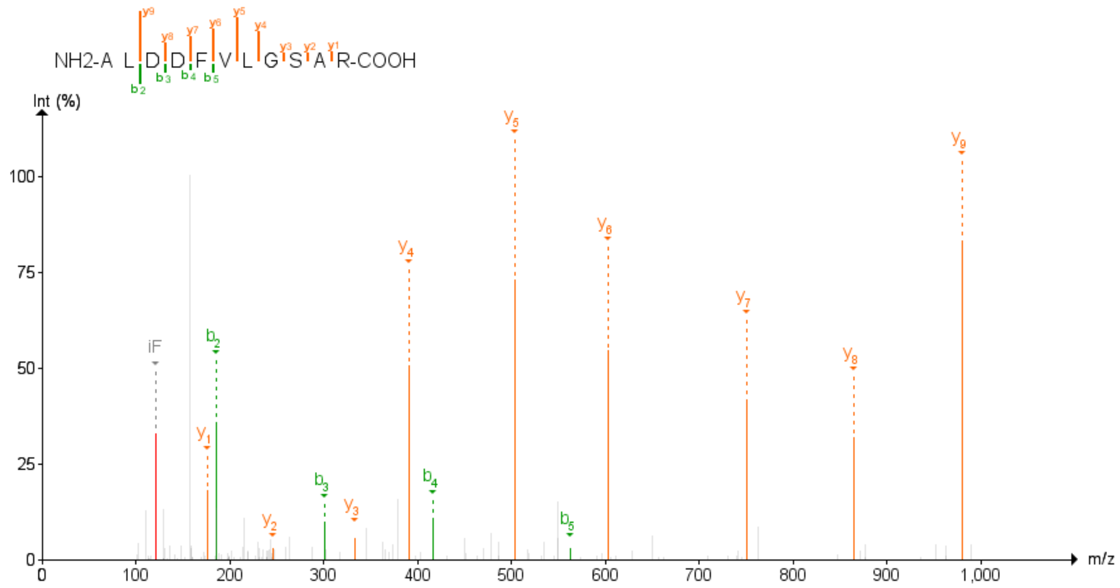

Figure 70: PRAF2 Second experiment

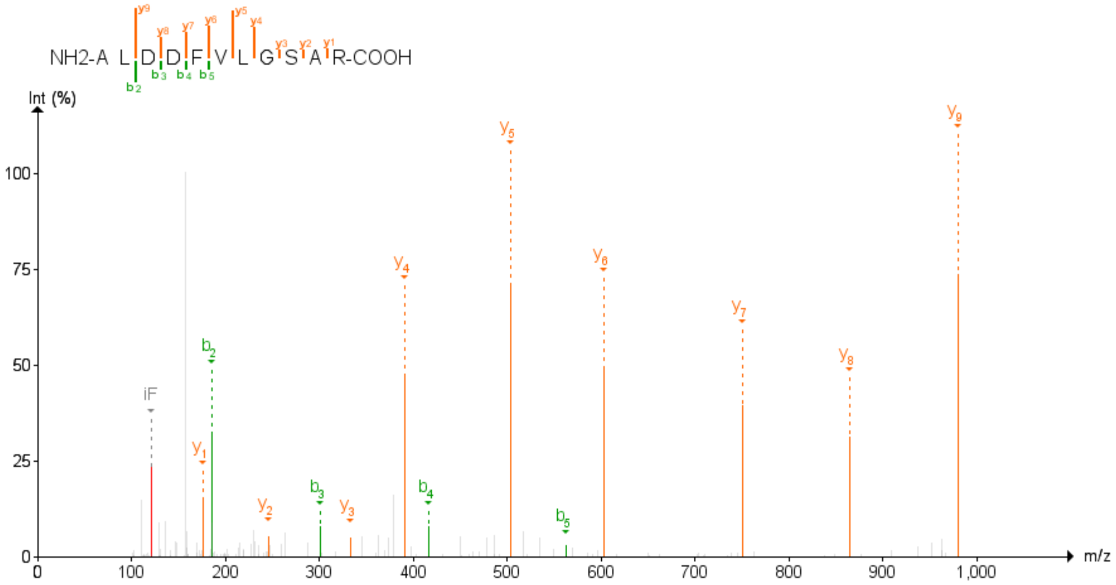

Figure 71: PRAF2 Third experiment

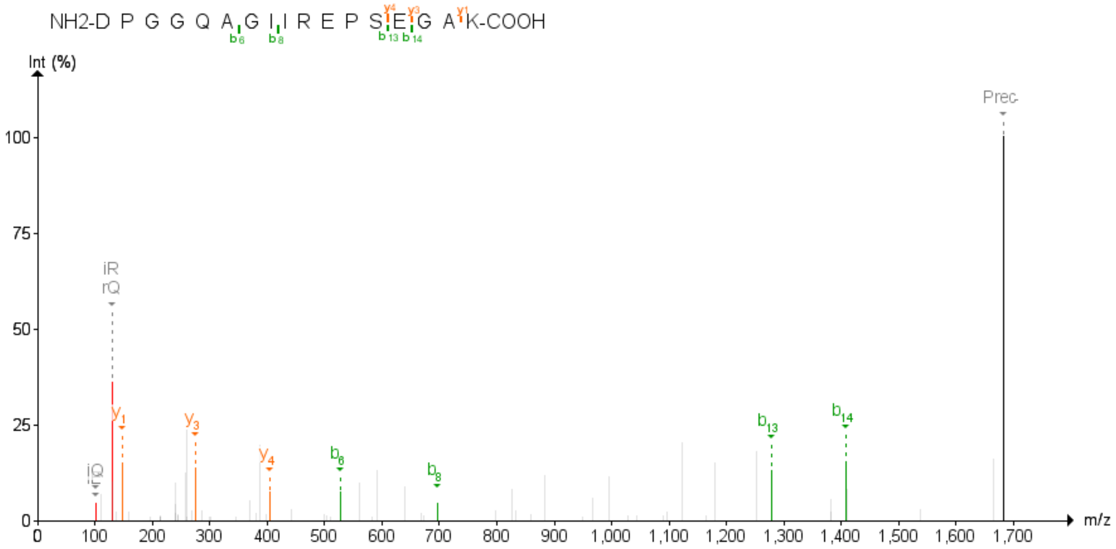

Figure 72: PRR14L First experiment

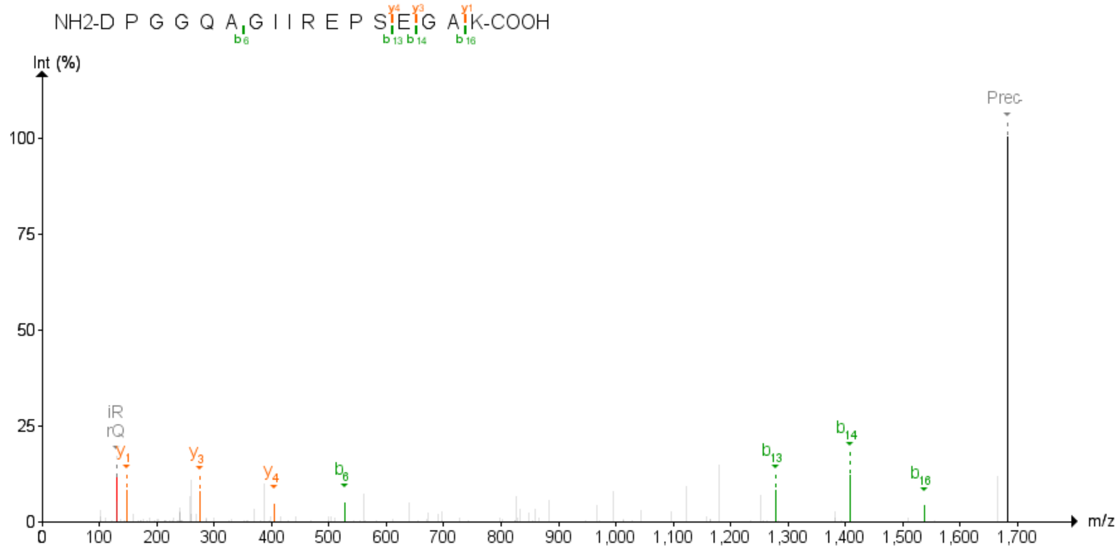

Figure 73: PRR14L First experiment

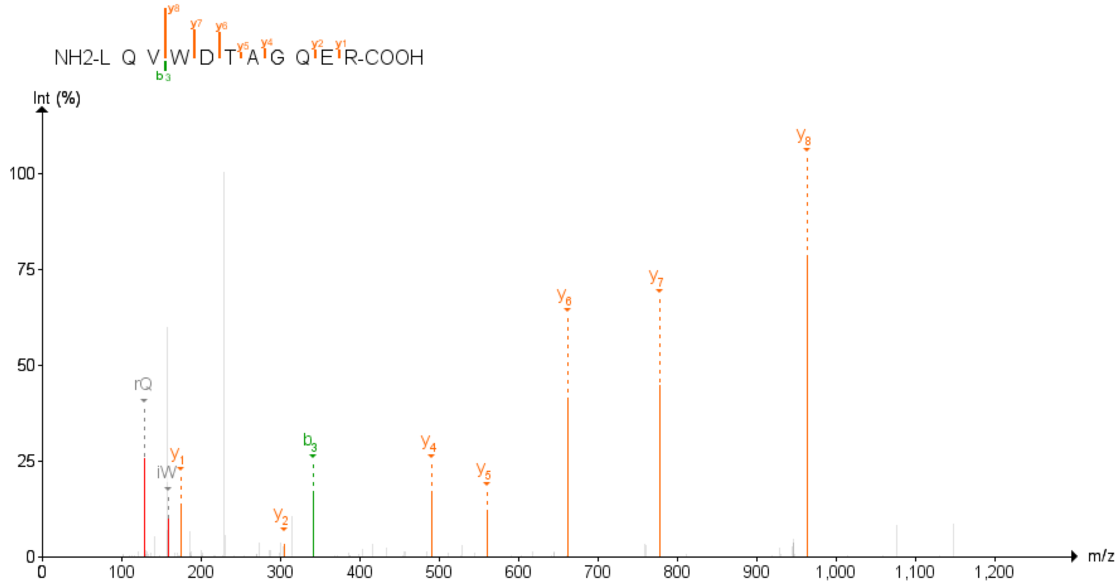

Figure 74: RAB13 Second experiment

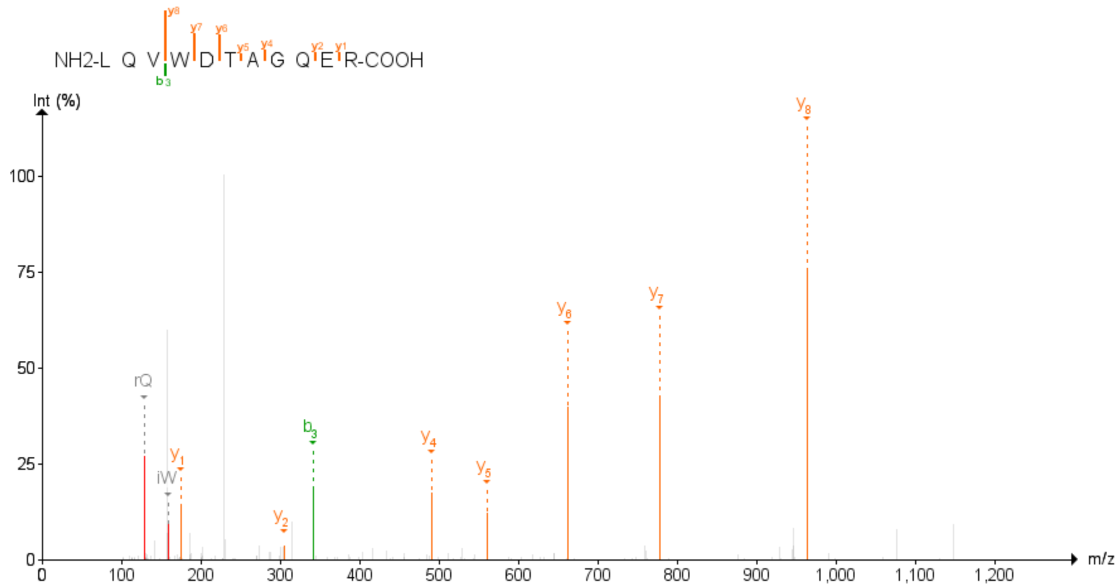

Figure 75: RAB13 Third experiment

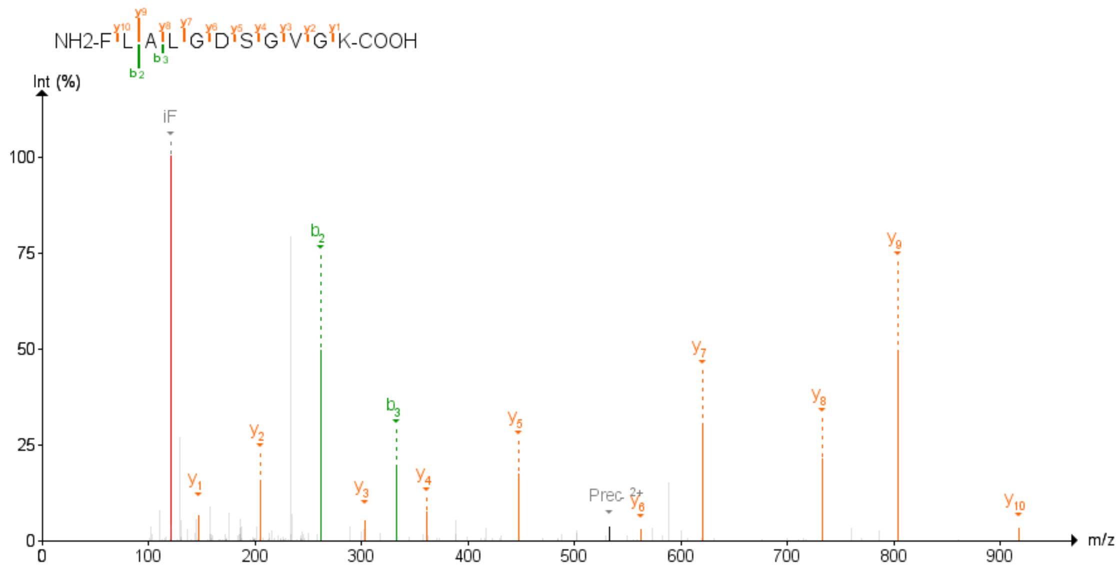

Figure 76: RAB27A Second experiment

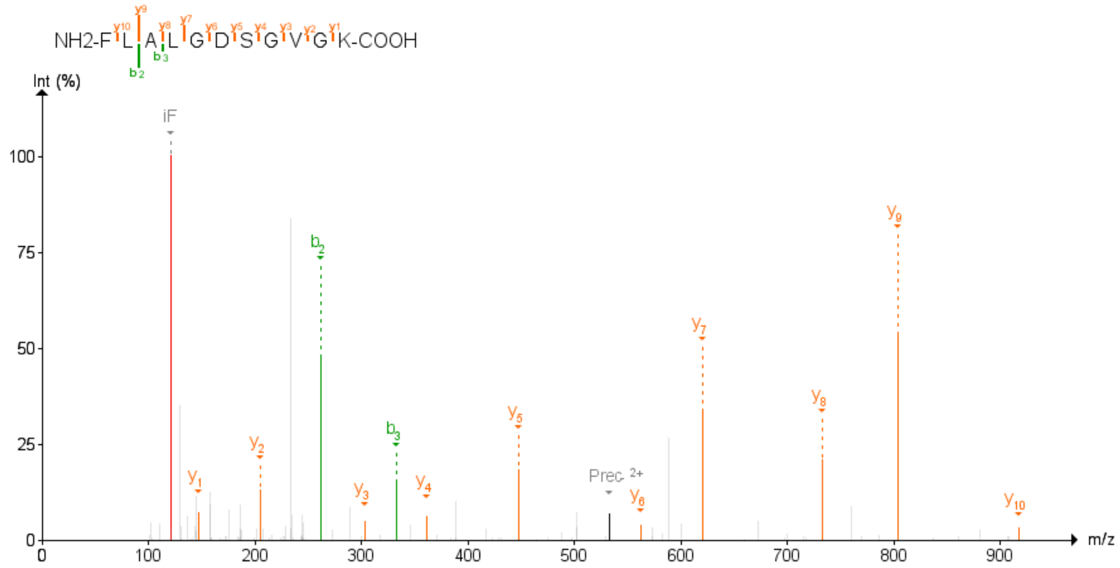

Figure 77: RAB27A Third experiment

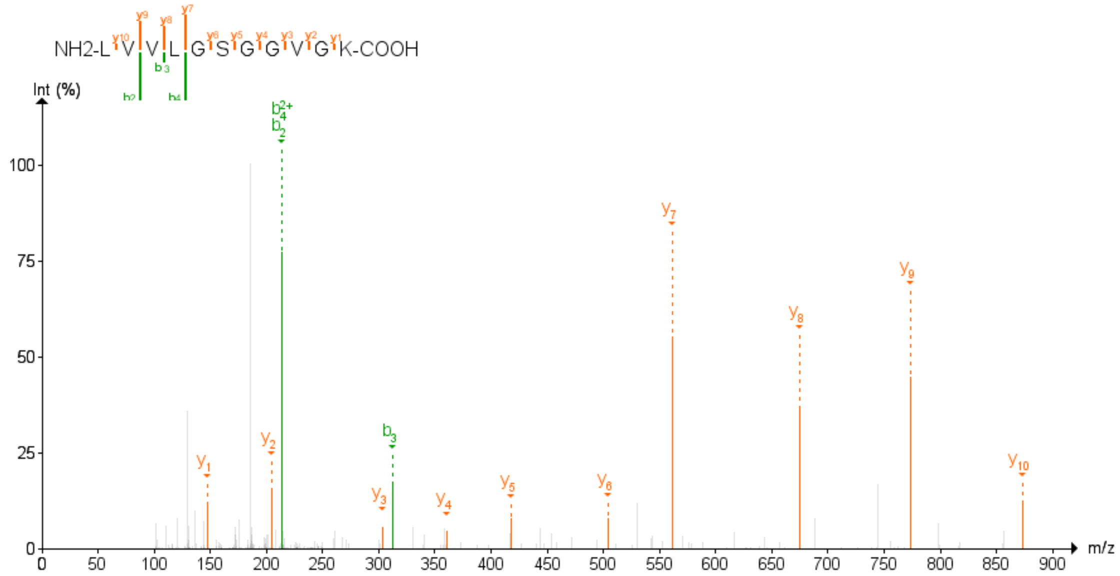

Figure 78: RAP1A;RAP1B Second experiment

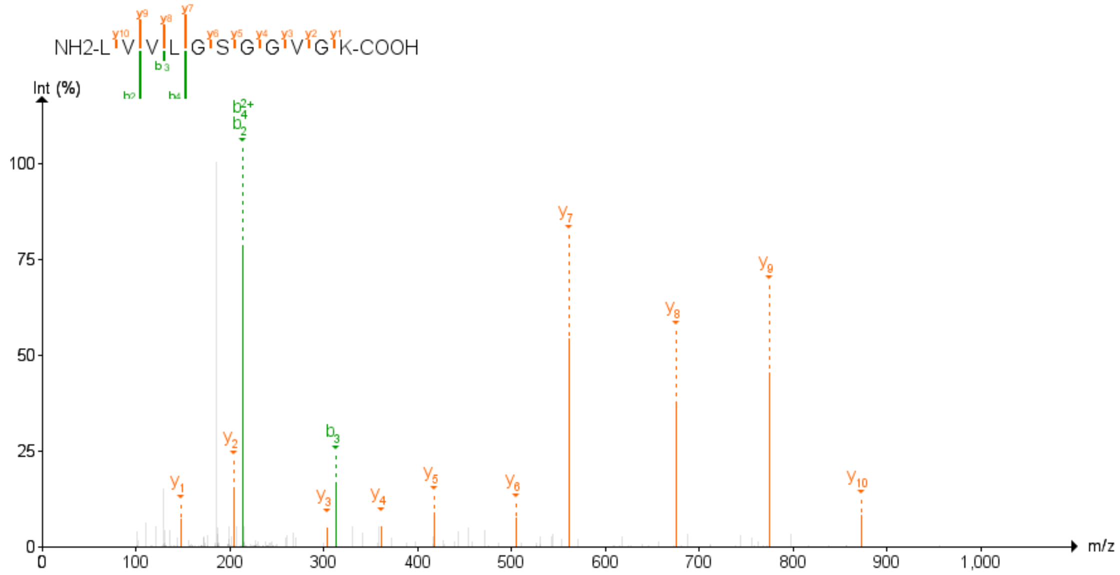

Figure 79: RAP1A;RAP1B Third experiment

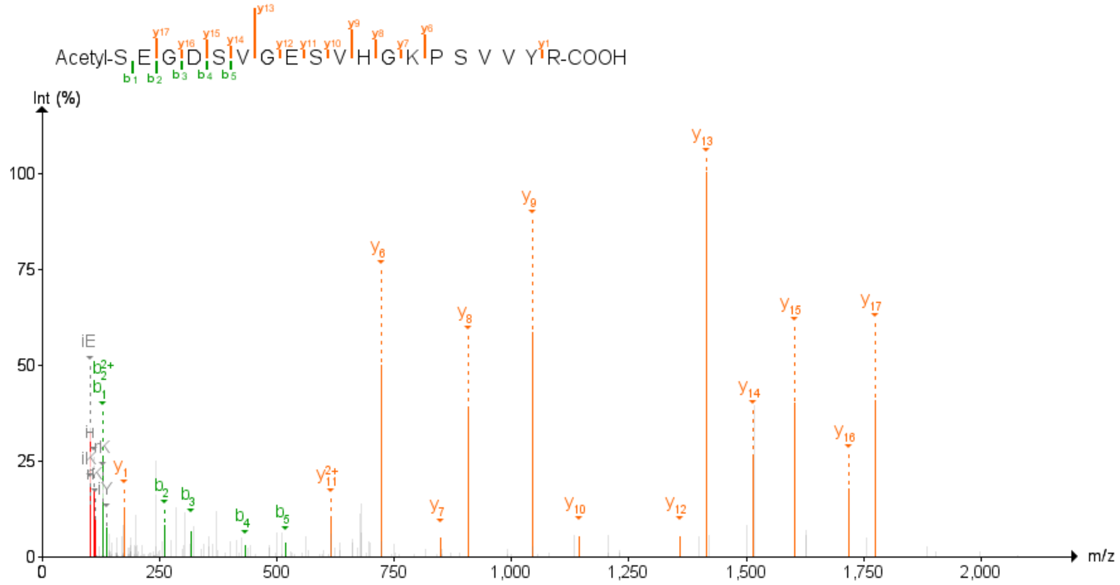

Figure 80: RER1 Second experiment

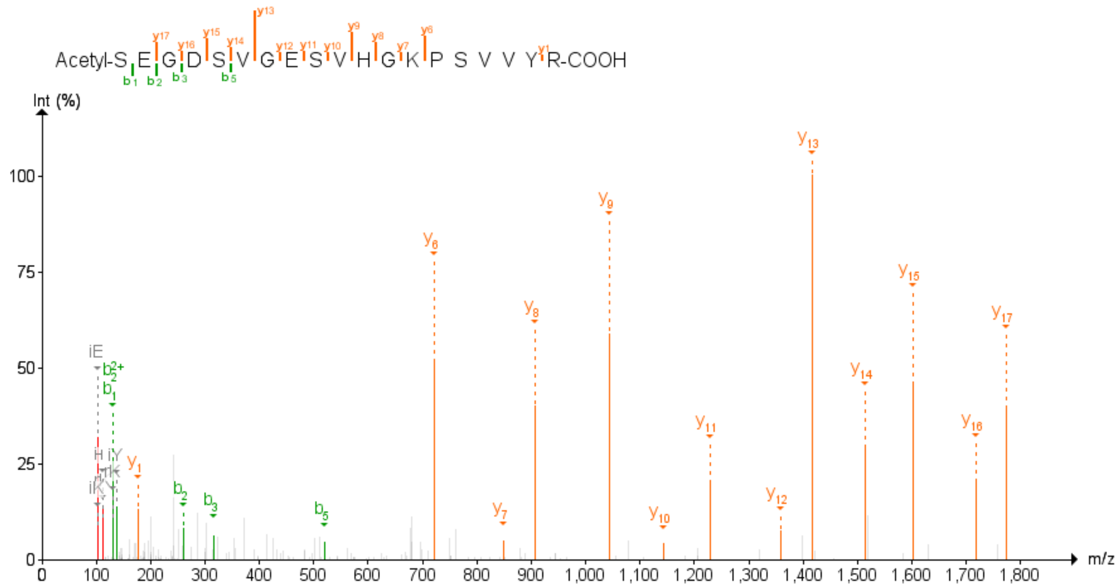

Figure 81: RER1 Third experiment

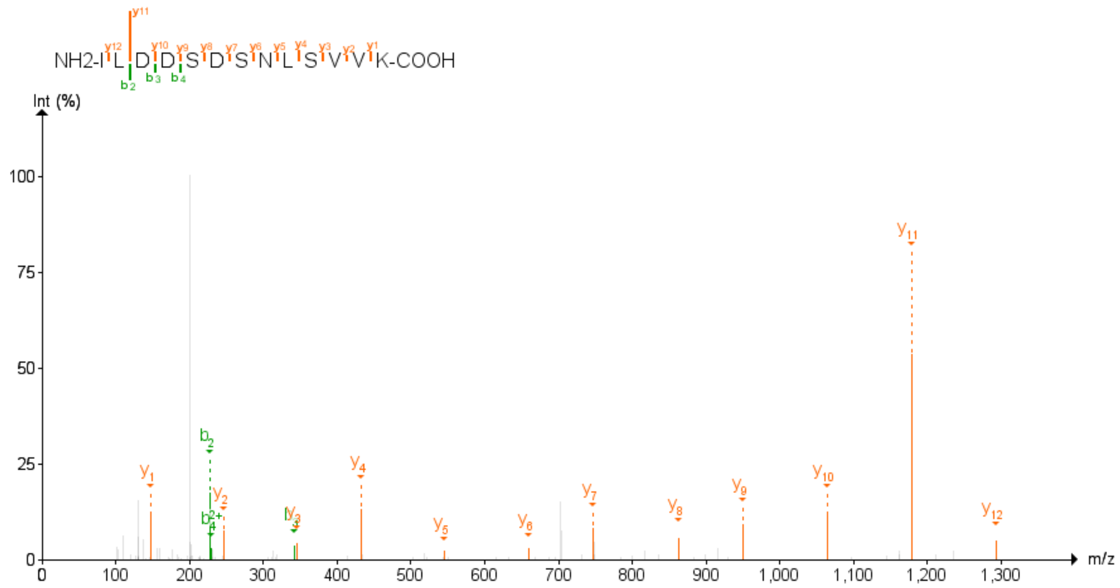

Figure 82: RGPD3;RGPD4 Second experiment

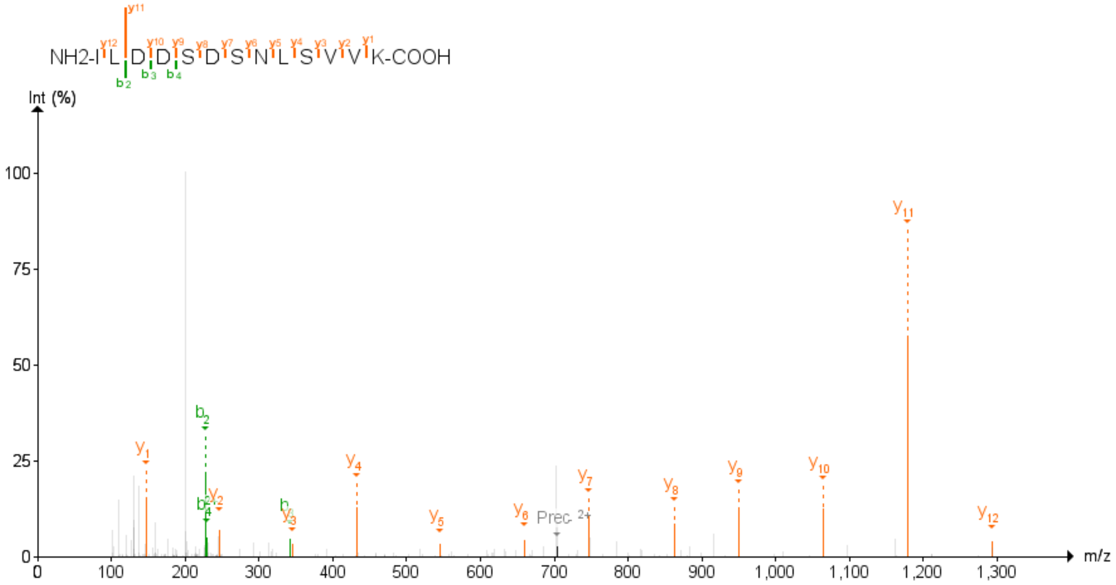

Figure 83: RGPD3;RGPD4 Third experiment

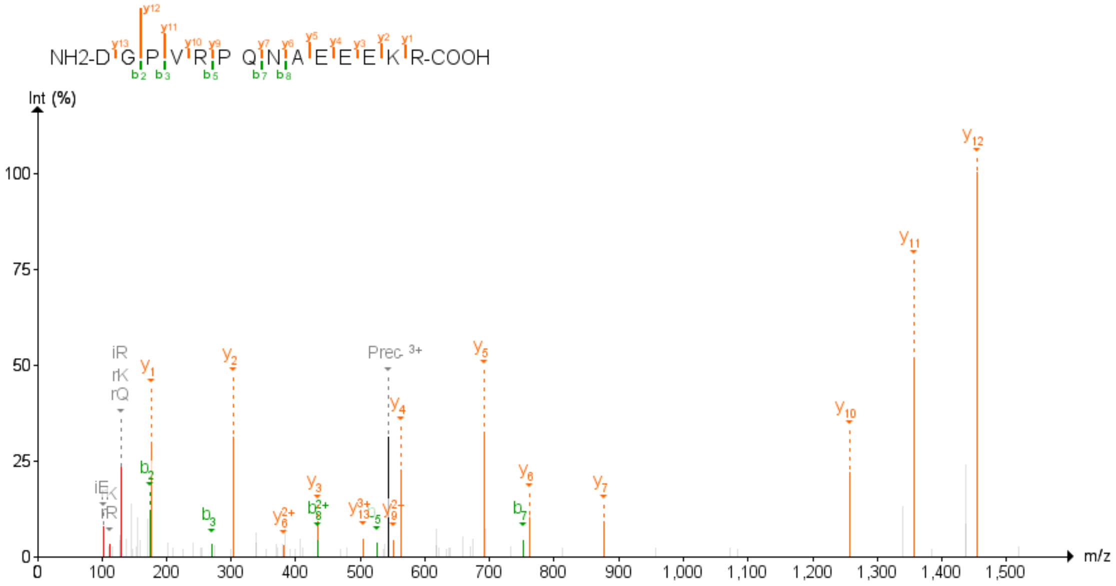

Figure 84: SLC39A7 First experiment

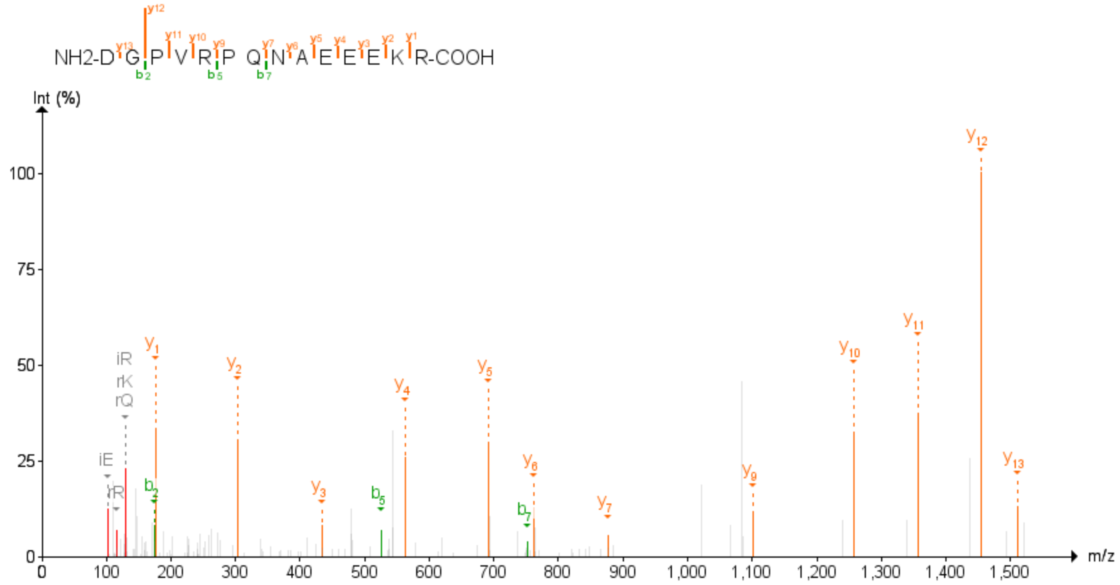

Figure 85: SLC39A7 Second experiment

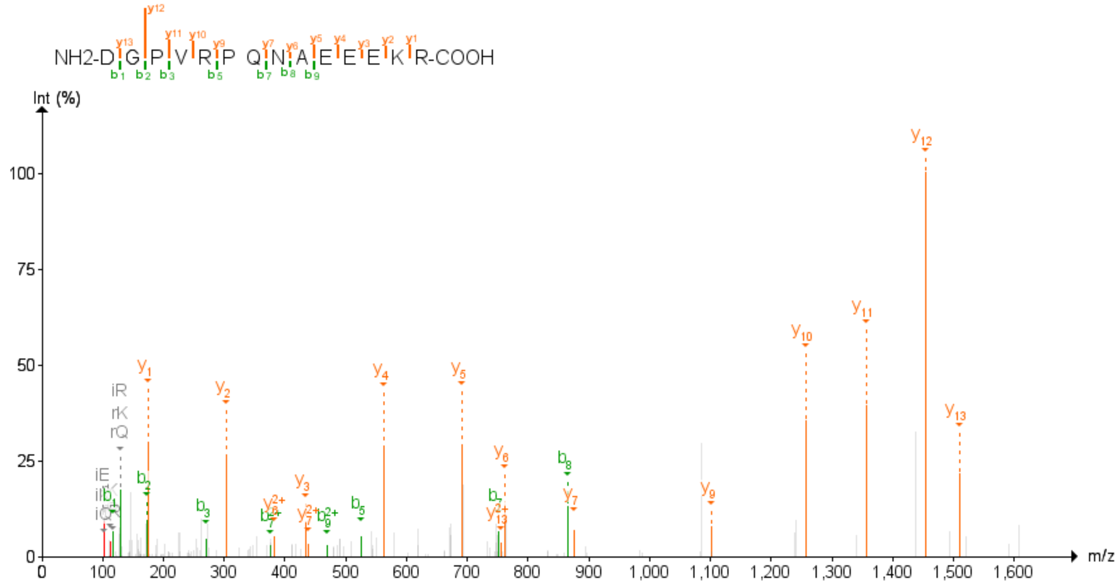

Figure 86: SLC39A7 Third experiment

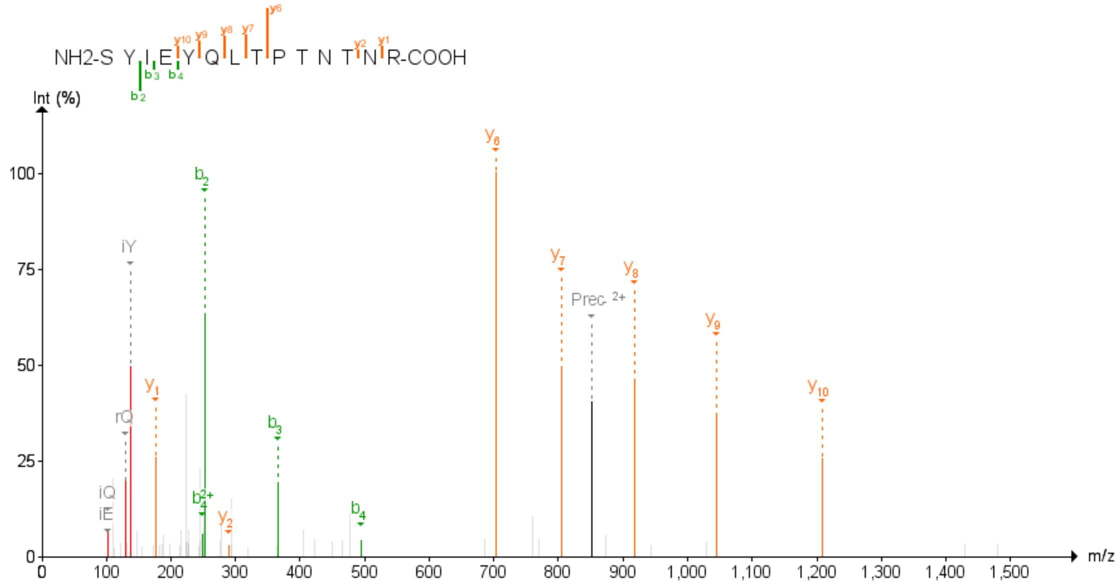

Figure 87: SNX9 First experiment

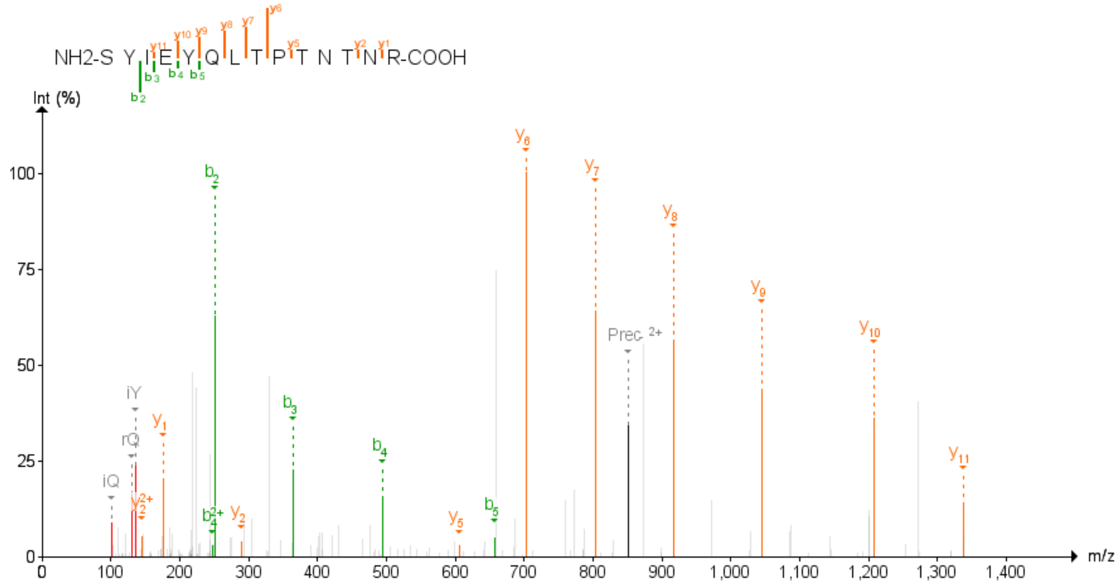

Figure 88: SNX9 Third experiment

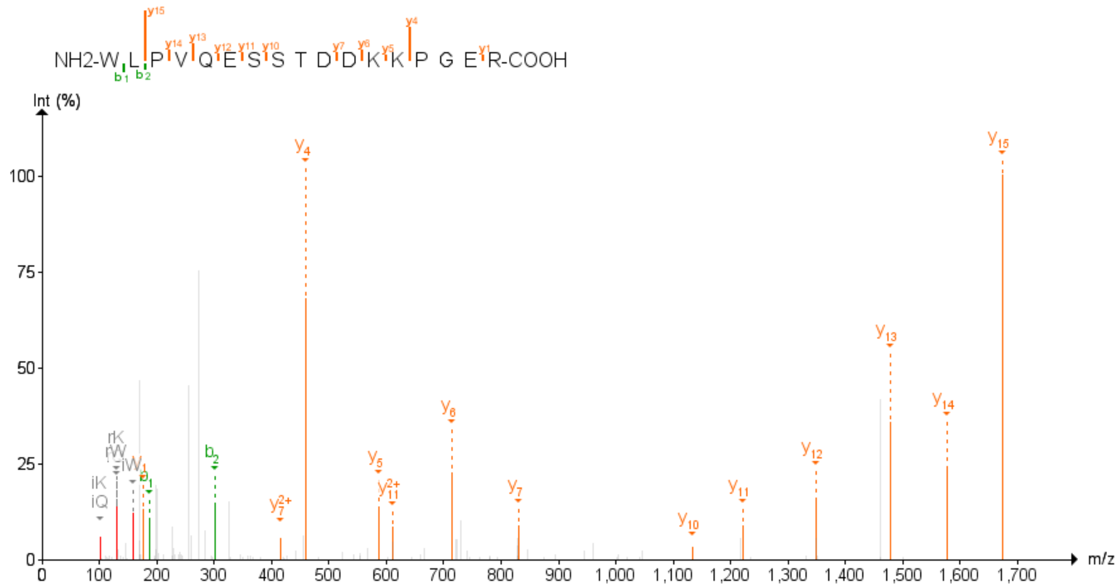

Figure 89: SPCS1 First experiment

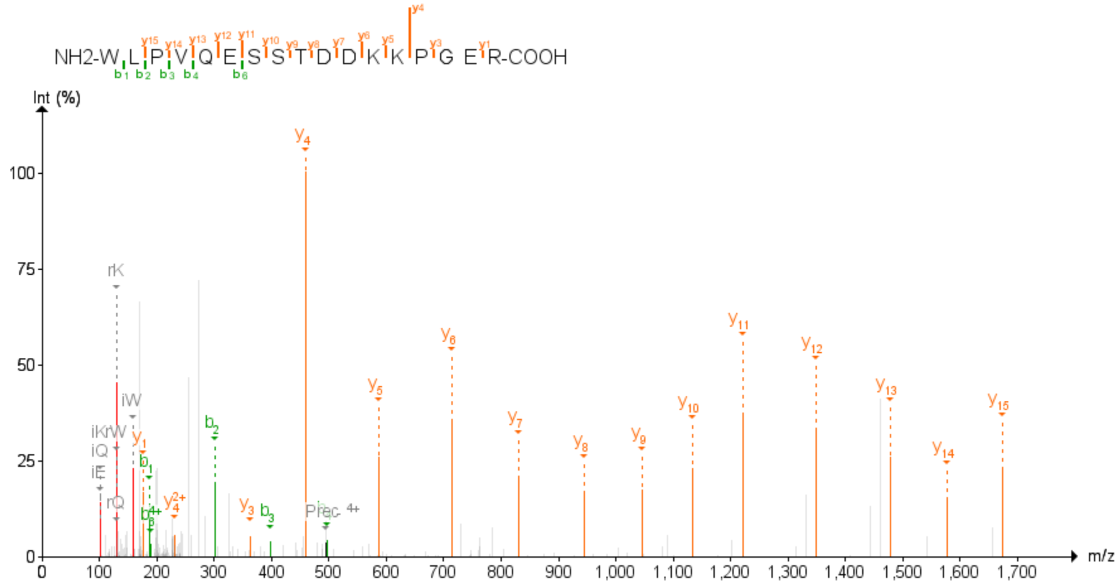

Figure 90: SPCS1 Second experiment

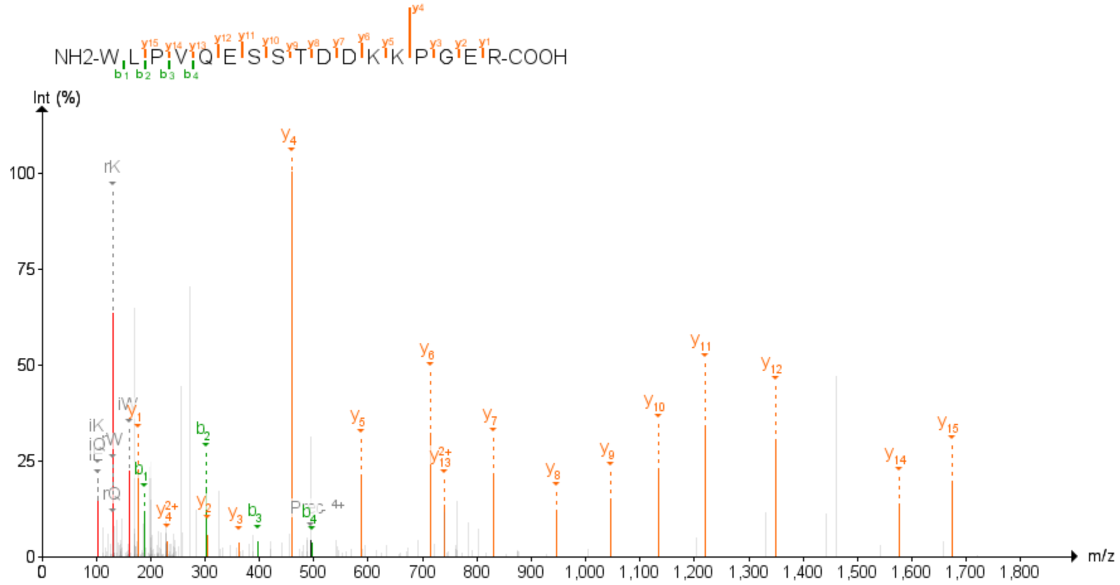

Figure 91: SPCS1 Third experiment

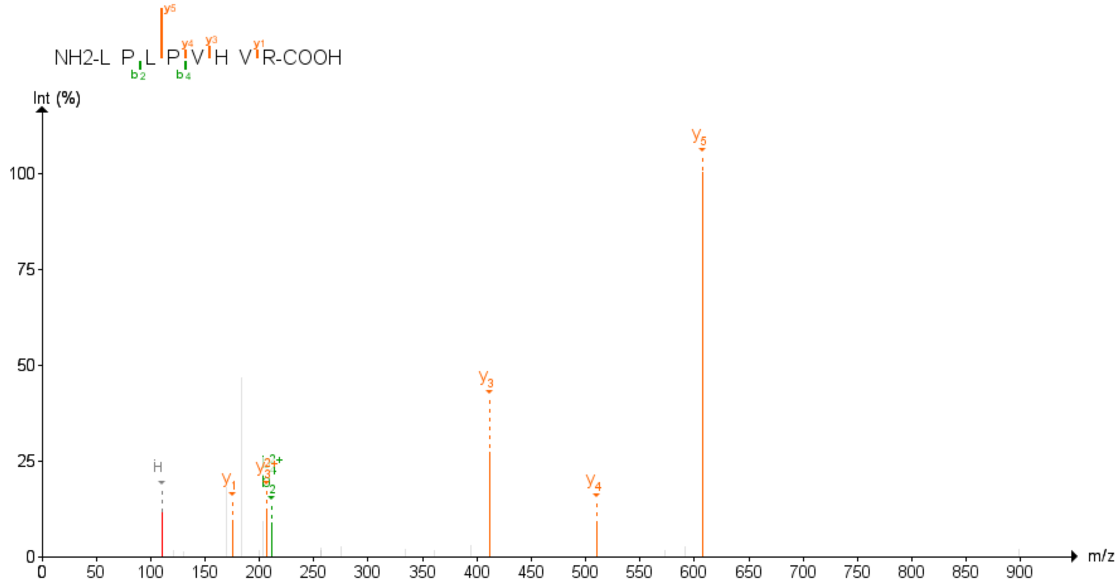

Figure 92: TLCD1 First experiment

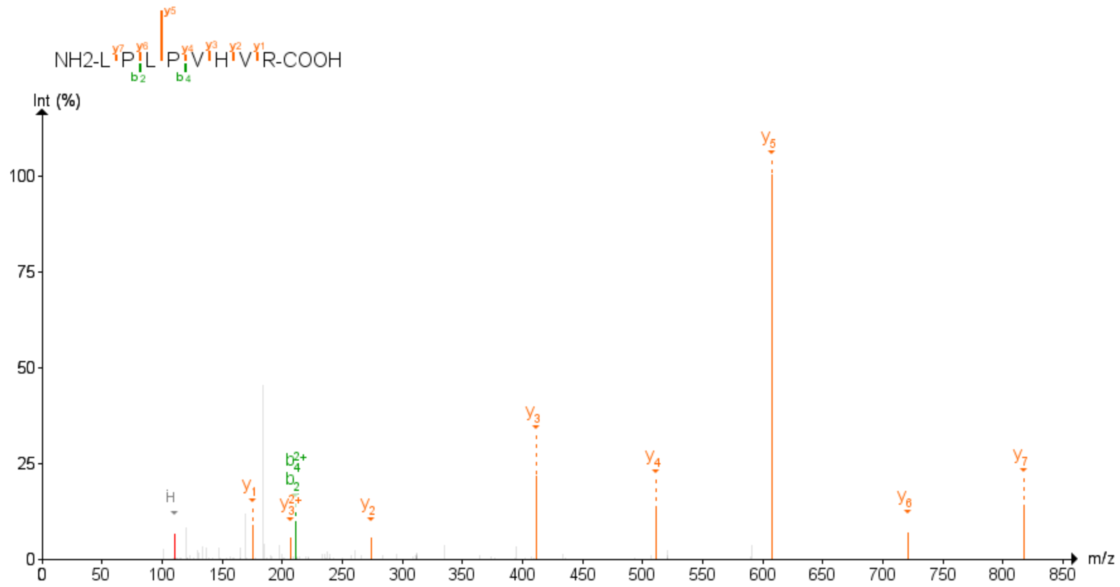

Figure 93: TLCD1 Third experiment

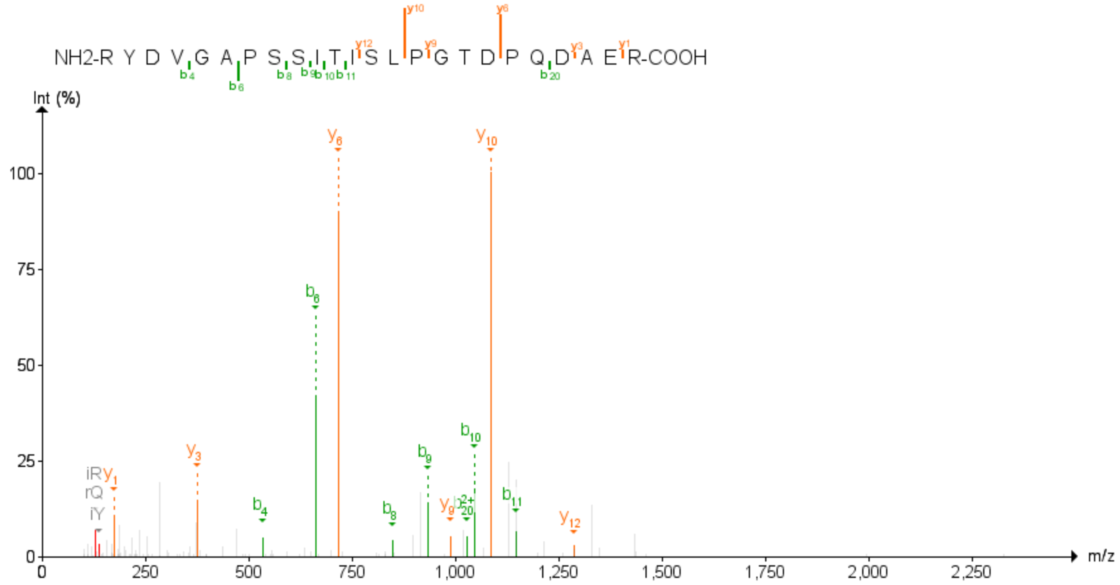

Figure 94: TMEM115 First experiment

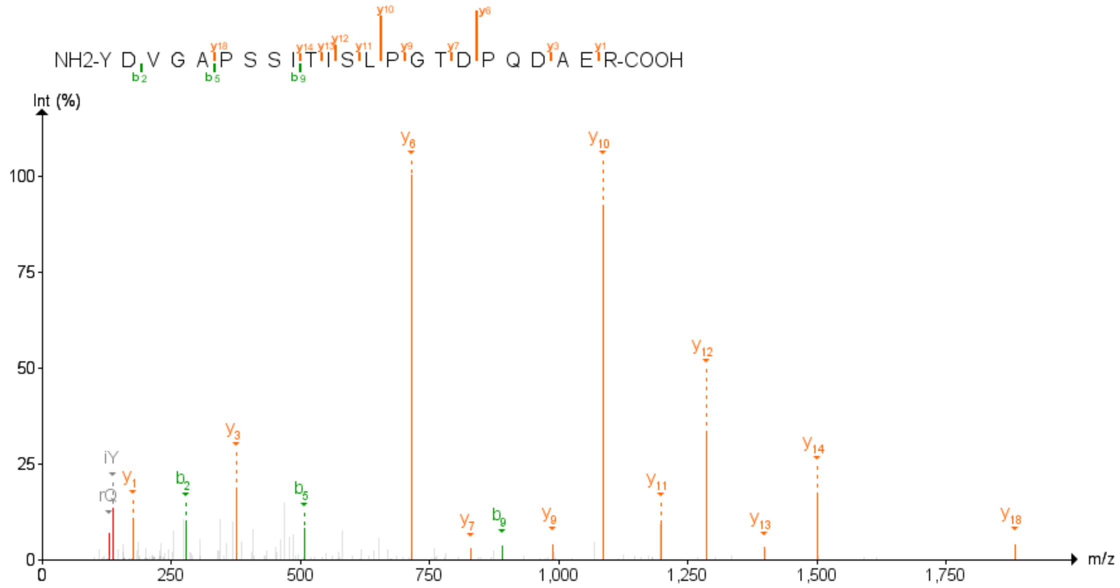

Figure 95: TMEM115 Second experiment

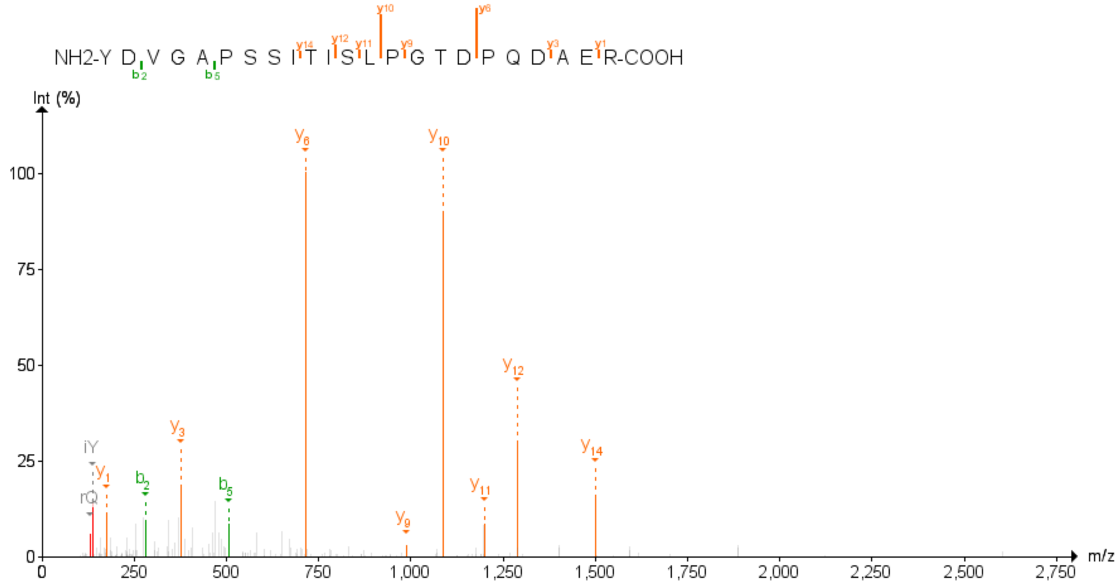

Figure 96: TMEM115 Third experiment

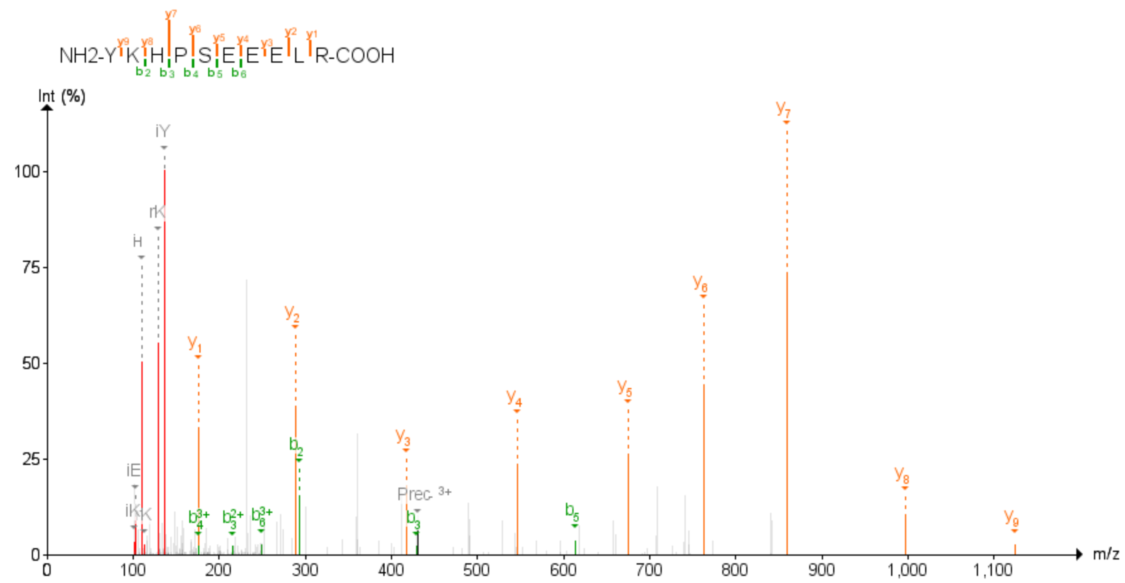

Figure 97: TMEM161A Second experiment

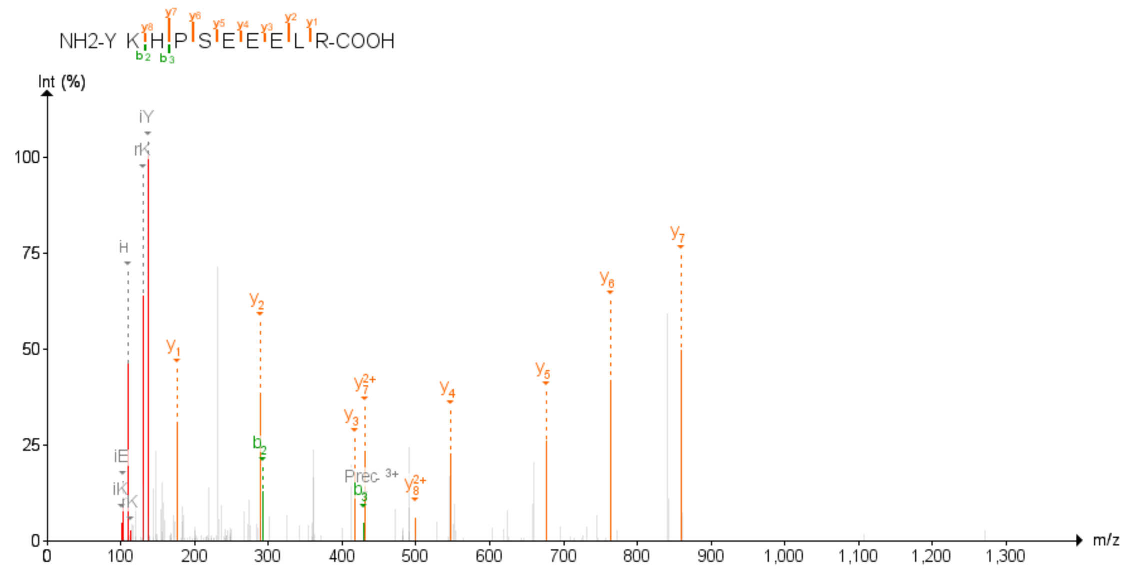

Figure 98: TMEM161A Third experiment

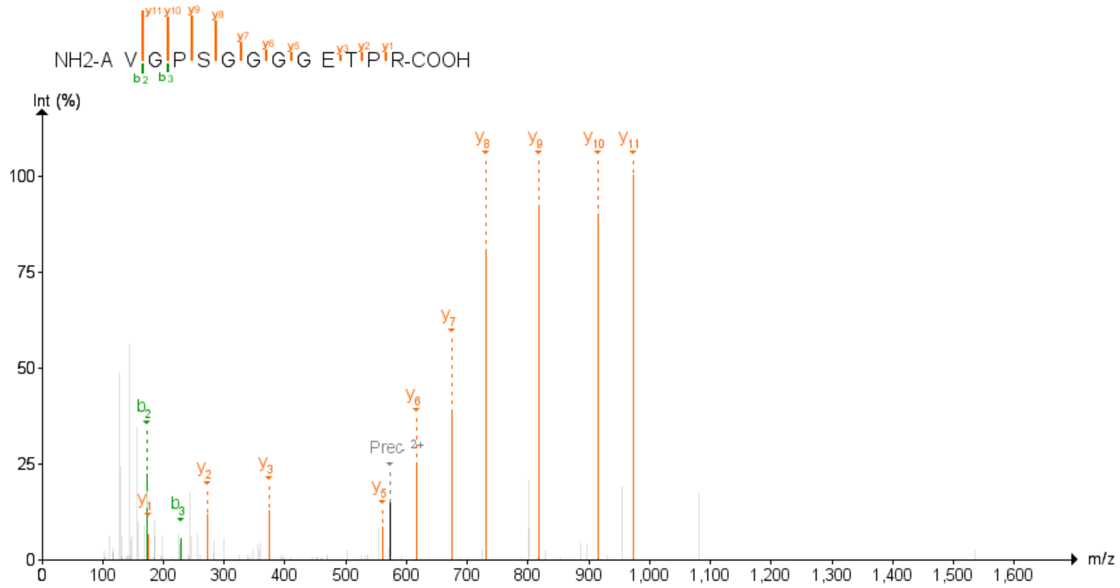

Figure 99: TMEM245 Second experiment

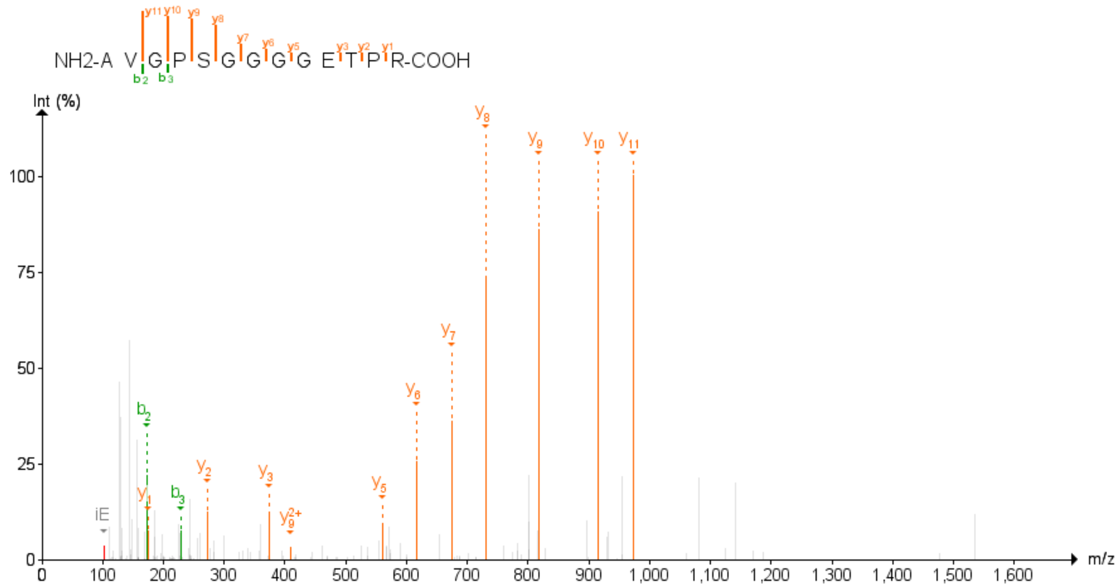

Figure 100: TMEM245 Third experiment

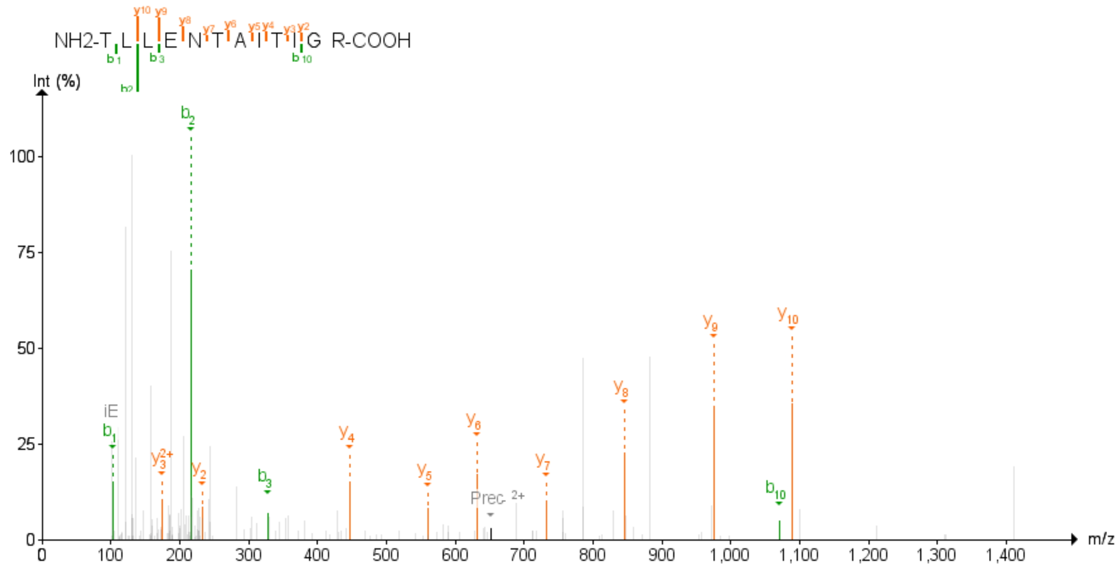

Figure 101: TNPO1 First experiment

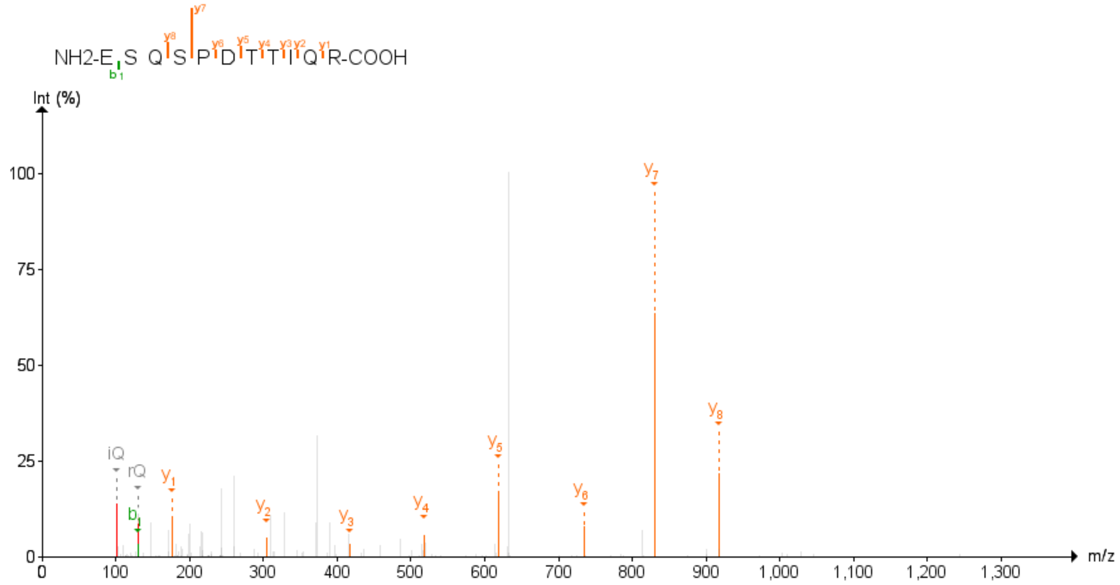

Figure 102: TNPO1 Second experiment

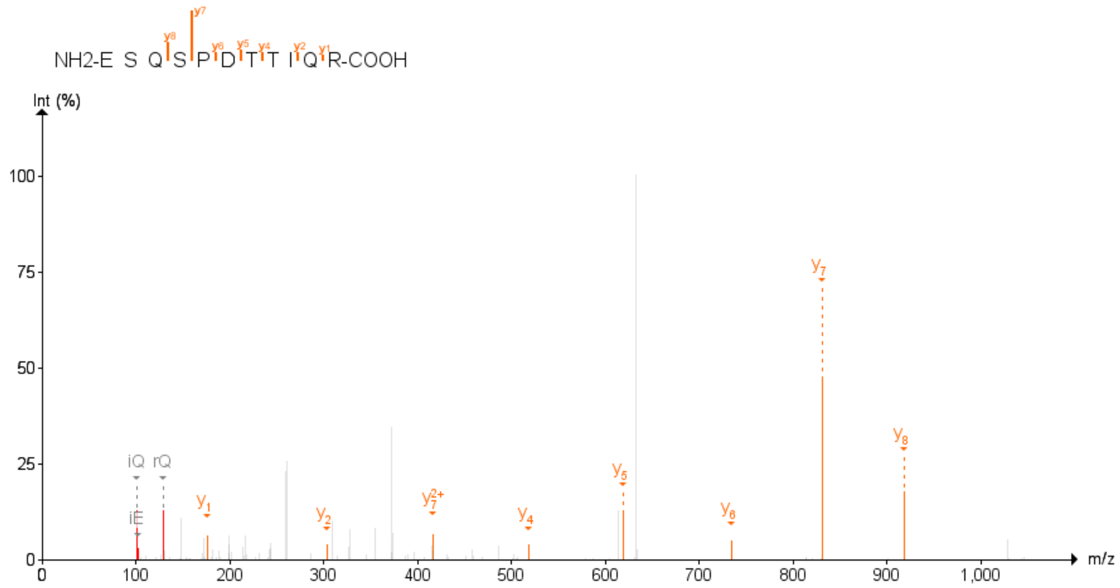

Figure 103: TNPO1 Third experiment

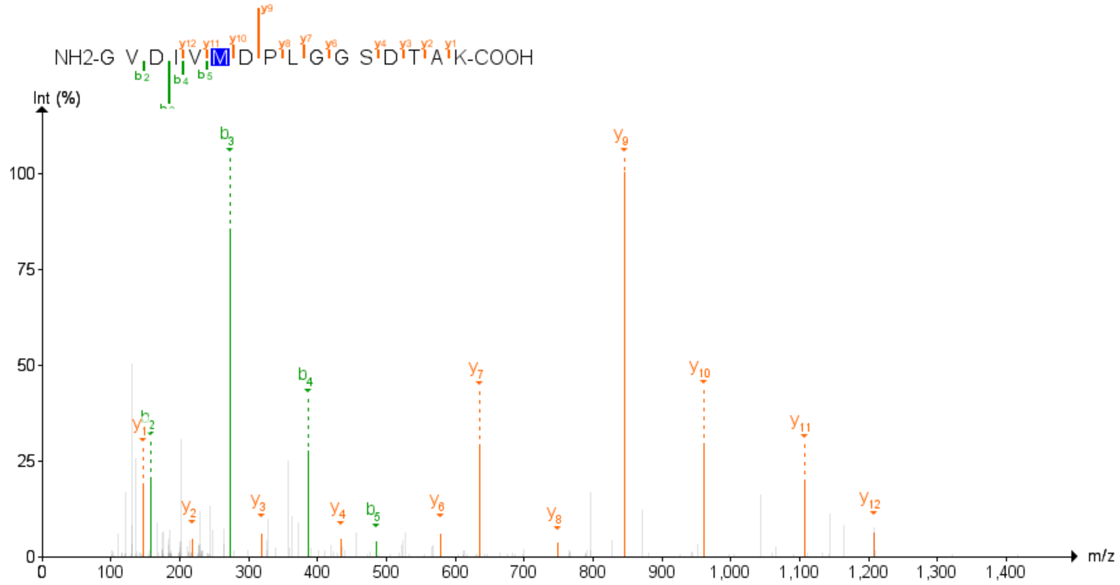

Figure 104: VAT1 Second experiment

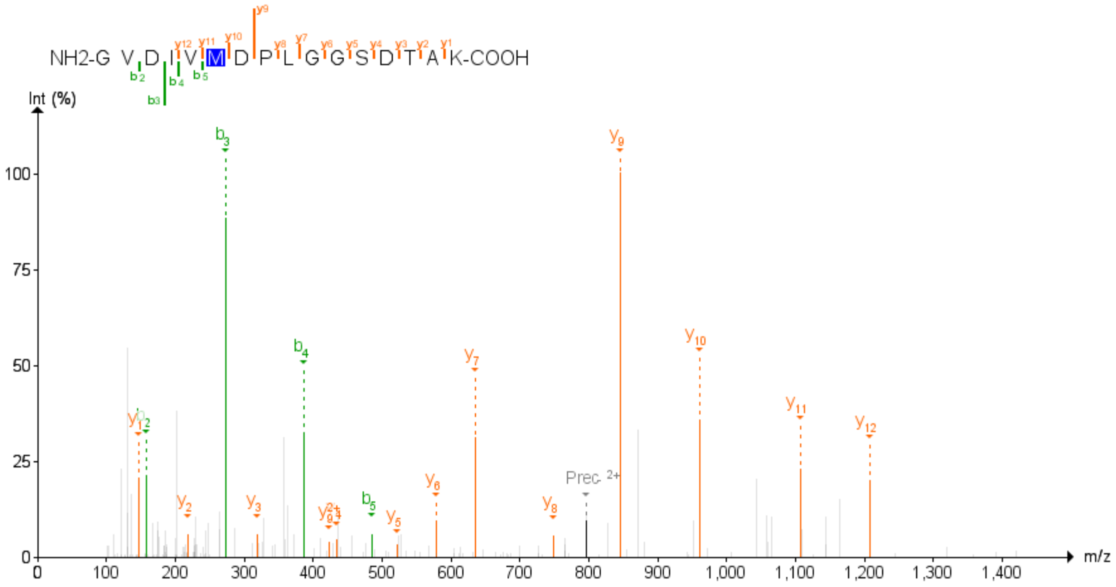

Figure 105: VAT1 Third experiment
